# Molecular survey of basidiomycetes and divergence time estimation: An Indian perspective

**DOI:** 10.1371/journal.pone.0197306

**Published:** 2018-05-17

**Authors:** Meghna Bhatt, Pankti Mistri, Ishita Joshi, Hemal Ram, Rinni Raval, Sruthi Thoota, Ankur Patel, Dhrupa Raval, Poonam Bhargava, Subhash Soni, Snehal Bagatharia, Madhvi Joshi

**Affiliations:** 1 Gujarat State Biotechnology Mission, Department of Science & Technology, Government of Gujarat, Gandhinagar, Gujarat, India; 2 Gujarat Biotechnology Research Center, Department of Science & Technology, Government of Gujarat, Gandhinagar, Gujarat, India; Oklahoma State University, UNITED STATES

## Abstract

This study outlines the biodiversity of mushrooms of India. It reveals the molecular biodiversity and divergence time estimation of basidiomycetes from Gujarat, India. A total of 267 mushrooms were collected from 10 locations across the state. 225 ITS sequences were generated belonging to 105 species, 59 genera and 29 families. Phylogenetic analysis of Agaricaceae reveals monophyletic clade of *Podaxis* differentiating it from *Coprinus*. Further, the ancient nature of *Podaxis* supports the hypothesis that gasteroid forms evolved from secotioid forms. Members of Polyporaceae appeared polyphyletic. Further, our results of a close phylogenetic relationship between *Trametes* and *Lenzites*lead us to propose that the genera *Trametes* may by enlarged to include *Lenzites*. The tricholomatoid clade shows a clear demarcation for Entolomataceae. However, Lyophyllaceae and Tricholomataceae could not be distinguished clearly. Distribution studies of the mushrooms showed omnipresence of *Ganoderma* and *Schizophyllum*. Further, divergence time estimation shows that Dacrymycetes evolved in the Neoproterozoic Era and Hymenochaetales diverged from Agaricomycetes during the Silurian period.

## Introduction

Fungi are ancient, diverse and abundant. Reported to have evolved 1800 million years ago [1], fungi have a wide range of morphologies. They may be unicellular (eg. yeast) or filamentous and possess a wide range of fruiting bodies (Sporocarps).With the estimated fungal diversity of more than 13 million, they are the largest biotic community after insects [2]. However, only 100,000 species of fungi are described of which the position of 25% of these is yet unresolved [3, 4]. Of these, more than 27000 species are reported from India [5, 6, 7].

Despite recent advances in molecular taxonomy the sequence information is far from complete in fungi [8, 9]. This information is of utmost importance in this group as fungi exhibits different morphological forms during the life cycle [10]. DNA barcoding has emerged to be an accepted technique for molecular identification of any class of organisms. The universally accepted locus for fungal identification is the ITS region of DNA [11] as it has interspecific variable but intraspecific conserved sequences, high copy number, short size (450 and 800 bp) and conserved priming sites [12]. Recently ITS2 has been reported to be sufficient for fungal identification specially during metabarcoding studies [12, 13]. Phylogenetic or molecular studies using nuclear ribosomal RNA genes (rDNA), such as the nuclear large subunit ribosomal RNA gene (nLSU) [14] and internal transcribed spacers 1 and 2 flanking the 5.8S region (nrITS) or combinations of the two [15] are lagging far behind in India. Moreover, there are no reports on the estimation of divergence time in the fungal taxa from India.

The evolutionary study can have a major advancement through the estimation of divergence time using the molecular data and existing fossil record. In case of fungi the fossil record is very poor, especially if one compares with plants and animals. This is largely attributed to the microscopic nature and the difficulty in recognizing them in the fossil record. Therefore, major lineages in fungi do not still have a fossil record. In this study we consider *Paleopyrenomycites devonicus*, the oldest unequivocal euascomycete fossil as the calibration point and estimate the divergence time using the molecular data generated [16–23].

In view of the above the present study deals with (i) identification and generation of barcodes for macrofungi from Gujarat, India, (ii) phylogenetic analysis for the identified species and (iii) estimation of the divergence time.

## Materials and methods

### Specimen sampling

**Fig 1:**
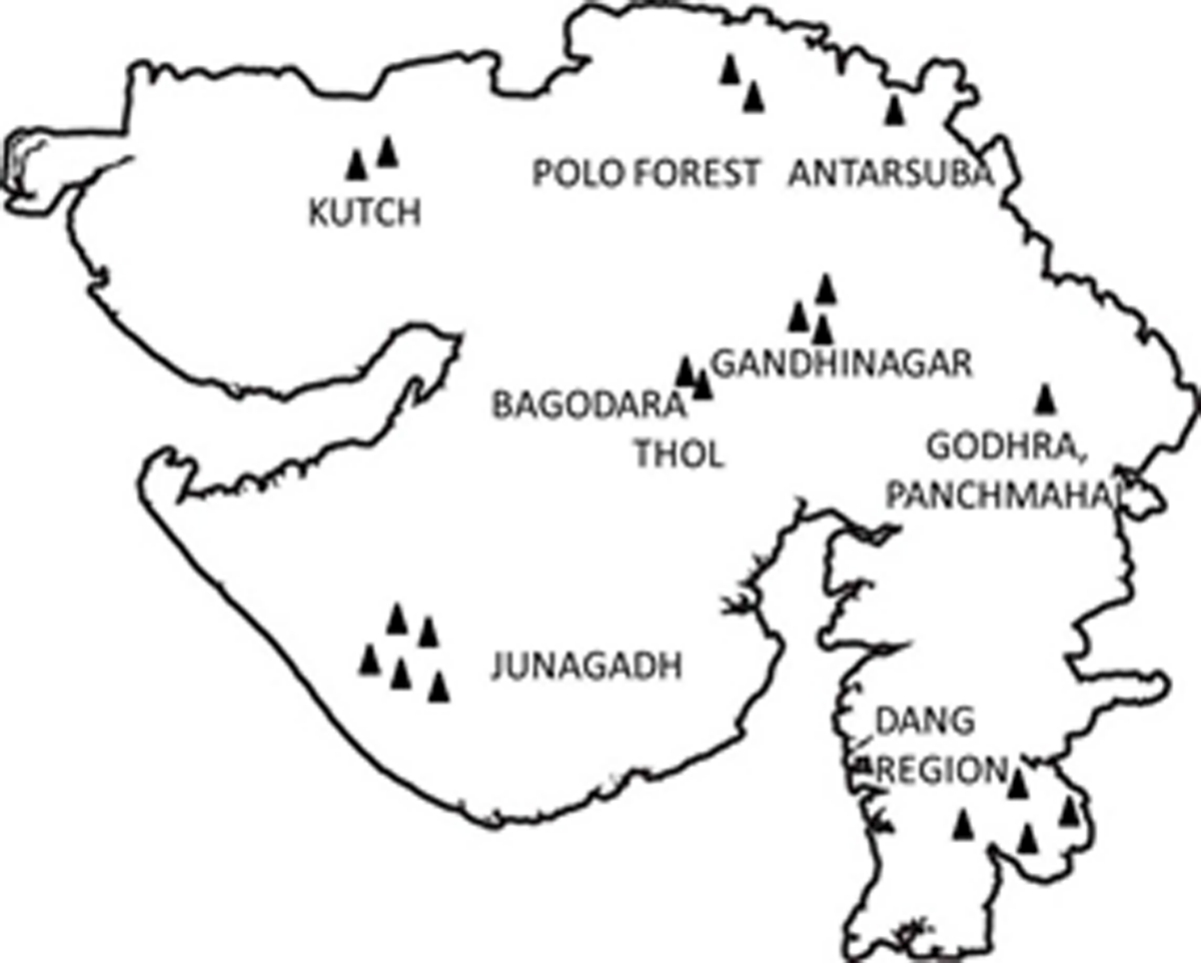
**Map of sampling localities to survey the mycotreasure–**Map showing the 10 locations of Gujarat from where macrofungi were collected.

In the present study, the samples were collected from ten different regions of Gujarat in an attempt to obtain barcode sequences for the largest possible proportion of various species in the collection. No specific permissions were required for these locations/activities and field studies did not involve endangered or protected species. The geographical locations of the sampled sites are shown in Fig 1 and the geographic coordinates of each site are presented in Table 1.

**Table 1:** Sampling sites of Gujarat used in this study.

Sr. No.	Sampling Sites	Latitude	Longitude	Elevation (m)
1	Gandhinagar	N 23 14' 09.8"	E 072 40' 46.4"	80
2	Thol, Ahmedabad	N 23 08' 37.7"	E 072 23' 33.7"	51
3	Polo forest, Sabarkantha	N 24 00' 00.8"	E 073 16' 51.6"	309
4	Antarsuba, Sabarkantha	N 23 59' 08.3"	E 073 12' 35.3"	276
5	Waghai, Dang	N 20 45' 09.4"	E 073 29' 55.2"	150
6	Ahwa forest, Dang	N 20 72' 96.0	E 073 68' 34.0	472
7	Saputara, Dang	N 20 34' 35.1"	E 073 44' 21.5"	957
8	Godhra, Panchmahal	N 22 54' 05.7"	E 073 03' 20.3"	109
9	Junagadh	N 21 28' 14.9"	E 070 33' 98.2"	107
10	Kutch	N 23° 56' 4.1''	E 069° 48' 52.1''	110

Although the collection techniques employed for collecting mushrooms were not complex, various precautions were taken while collecting. Before collecting a mushroom from the site, its habitat and substrate were noted down. In addition, photographs of mushrooms in their natural habitat and in natural daylight were taken. The mushrooms were collected with sterile forceps. The individual mushroom was packed in wax papers and then transferred in bags. Smaller mushrooms were directly collected in sterile sample containers and were sealed tightly to keep them moist and undamaged. All the samples were properly labeled and carried to the laboratory for further processing.

### DNA extraction, PCR and Sequencing

Genomic DNA was extracted using NucleoSpin® 96 well plant II vacuum processing (Macherey-Nagel) Kit, for fungi according to manufacturer’s protocol. The ITS region was amplified via PCR using ReadyMix™ Taq PCR Reaction Mix (Sigma) with the primers ITS2-S2F (5’-ATGCGATACTTGGTGTGAAT-3’) and ITS4 (5’-CCTCCGCTTATTGATATGC-3’). A subset of samples not yielding ITS2-S2F/ITS4 amplicons were amplified using the primer pairs ITS8-F (5’-AGTCGTAACAAGGTTTCCGTAGGTG-3’) and ITS6-R (5’-TTCCCGCTTCACTCGCAGT-3’) [18] and ITS1 (5’-3’) and ITS4 (5’-CCTCCGCTTATTGATATGC-3’) [24–25]. PCR reaction mixtures were prepared in 20 μL volumes including 10 μL (2X) ready mix (Sigma–Aldrich), 1μL each primer (10pM), 50 ng/ μL template DNA, 1.2 μL MgCl_2_ (25 mM), and sterile MilliQ water to reach 20 μL total. Amplification conditions were as follows: 95°C for 5 minutes, 35 cycles of 95°C for 30 seconds, 56°C for 45 seconds, 72°C for 1.5 minutes and 72°C for 10 minutes. PCR products were purified using GenElute PCR purification kit (Sigma Life science). Sequencing reactions were conducted using BigDye v3.1 dye terminator chemistry with the primer pairs ITS2-S2F/ITS4, ITS1/ITS4 and ITS8F/ITS6R and analyzed using ABI 3500XL sequencer (Applied Biosystems).

#### Data analysis and sequence submission

The raw DNA sequences were assembled with BioEdit 7.2.5 [26]. The resulting alignment of 350–450 bp and 650–700 bp contained no gaps and all sequences were analyzed by performing BLAST to verify the identity. To infer phylogenetic relationships among these species, sequences were aligned using Muscle [27] with UPGMA as the clustering method using Mega6 [28]. The ITS1-5.8S-ITS2 regions were successfully sequenced for 225 species forming the dataset. Of these, 68 species were singleton species. Phylogenetic reconstruction was carried out using UPGMA as a statistical method with 1000 Bootstrap. Nucleotide substitution type and maximum composite likelihood model were used. Specimen data, trace files and sequences were deposited in BOLD (Barcode of Life Data Systems) in project Barcoding Fungal Biodiversity of Gujarat, India (project code: MGEN). All DNA sequences were also deposited in GenBank.

### Sequence analysis

The resulting225 sequences were analyzed using analytical functions available on Barcode of Life Data Systems (BOLD) including (a) Sequence composition (frequency of nucleotides) (Table 2), (b) Diagnostic characters–(identification of consensus bases from each group) and (c) Barcode gap analysis. A large DNA barcode gap makes it easy to distinguish among species, whereas small or negative barcoding gaps tend to hamper species assignation. The genetic distances were assessed by calculating pairwise nucleotide differences across the dataset and categorizing each comparison as either intraspecific or interspecific based on the specimen identification.

**Table 2:** Summary statistics for nucleotide distribution frequency for input sequences.

	Min	Mean	Max	SE
G%	17.24	22.05	29.42	0.13
C%	17.81	23.97	28.01	0.1
A%	20.56	29.41	35.43	0.17
T%	18.88	24.57	32.64	0.16
GC%	35.72	46.02	57.43	0.2
GC % Codon Pos 1	32.12	45.99	60.56	0.29
GC % Codon Pos 2	32.09	45.97	57.02	0.28
GC % Codon Pos 3	33.84	46.09	59.62	0.28

### Phylogenetic reconstructions

Phylogenetic trees were constructed using MEGA version 7 using UPGMA, maximum likelihood and maximum parsimony programs. All positions containing gaps and missing data were eliminated. For the maximum parsimony program the evolutionary distance was calculated using Tamura-Nei model [29]. The tree with the highest log likelihood was taken. Initial tree(s) for the heuristic search were obtained automatically by applying Neighbor-Join and BioNJ algorithms to a matrix of pairwise distances estimated using the Maximum Composite Likelihood (MCL) approach, and then selecting the topology with superior log likelihood value. The Maximum Parsimony tree was obtained using the Subtree-Pruning-Regrafting (SPR) algorithm with search level 1 in which the initial trees were obtained by the random addition of sequences (10 replicates). UPGMA tree was constructed which assumes that the rate of evolution has remained constant throughout the evolutionary history of the included taxa thereby producing a rooted tree. The support of monophyletic groups was assessed by the bootstrap method with 500 replicates.

### Divergence time estimation

Due to limited and sporadic fossil records in fungi, it has been difficult to choose a reliable calibration point for the divergence time estimation of any fungal group. Based on the previous molecular studies carried out on different fungal groups have stated the divergence between Ascomycota and Basidiomycota might have occurred in the Cambrian period. In this study, we have taken the divergence between Ascomycota and Basidiomycota (i.e. 582 mya) as the calibration point which is supported by recent molecular studies on basidiomycetous fungi [30–31], which is based on a 400 million-year-old fossil, *Paleopyrenomycites devonicus*. The BEAST 1.8.0 [32] software package was used to estimate divergence time. We first generated XML files executable in BEAST using BEAUti. The ITS dataset was then set for two partitions with substitution and molecular clock models unlinked while linking the trees. The strict molecular clock model and the constant size coalescent prior set were used to estimate the divergence times and the corresponding credibility intervals. The posterior distributions of parameters were obtained using MCMC analysis for 50 million generations with a burn-in percentage of 10%. Samples from the posterior distributions were summarized on maximum clade credibility (MCC) tree with the maximum sum of posterior probabilities on its internal nodes using TreeAnnotator v1.8.0. The posterior probabilities limit was set to 0.5 to summarize the mean node heights. FigTree v1.4.2 was used to visualize the resulting tree and to obtain the means and 95% higher posterior densities (HPD). A 95% HPD marks the shortest interval that contains 95% of the values sampled.

## Results

### Taxon diversity

Nearly 267 mushroom samples belonging to Agaricales, Polyporales order of Basidiomycota along with additional orders of Basidiomycota as well as Ascomycota were collected, out of which 225 mushrooms comprised of 105 species, 59 genera and 29 families. S1 Fig represents photographs for all the collected mushroom specimens, in the case of multiple specimens, representative images are shown. Of the 105 species identified, 37 species were represented by multiple samples with a maximum of 19 and 17 individuals of *Ganoderma multipileum* Ding Hou and *Schizophyllum commune* Fries respectively. The remaining 68 species had a single ITS barcode. The collection included 93% of mushroom species that were being first time submitted in BOLD systems. In addition, there are six species namely *Clitopilus prunulus* (Scop. ex Fr.) P. Kumm., *Pleurotus ostreatus* (Jacq. ex Fr.) P. Kumm, *Psathyrella candolleana* (Fr.) Maire, *Schizophyllum commune*, *Auricularia polytricha* (Mont.) Sacc. and *Daedaleopsis* confragosa (Bolton) J.Schröt. being submitted for the first time from India to BOLD systems. Process IDs of specimen collected and accession numbers are provided in S1 Table.

### Assessment of ITS sequence at family level

The ITS1-5.8S-ITS2 regions were successfully sequenced for 225 specimens forming the dataset. All the 225 specimens belonged to 29 families. Of these, 189 individuals were recorded from eleven families namely Agaricaceae, Polyporaceae, Ganodermataceae, Schizophyllaceae, Tricholomataceae, Entolomataceae, Lyophyllaceae, Fomitopsidaceae, Xylariaceae, Psathyrellaceae and Meruliaceae and the remaining 36 individuals were recorded from 18 other families. The phylogenetic tree was reconstructed using Mega6 for all these eleven families to infer the evolutionary relationships among the species of Gujarat. Although there was some difference in the degree of resolution when UPGMA, Maximum Likelihood and Maximum Parsimony were used, the topologies obtained were consistent. Moreover UPGMA method gave a better bootstrap support as compared to the Maximum parsimony and Maximum Likelihood. The Maximum Likelihood and Maximum Parsimony trees are shown in S2A–S2N Fig.

**Fig 2:**
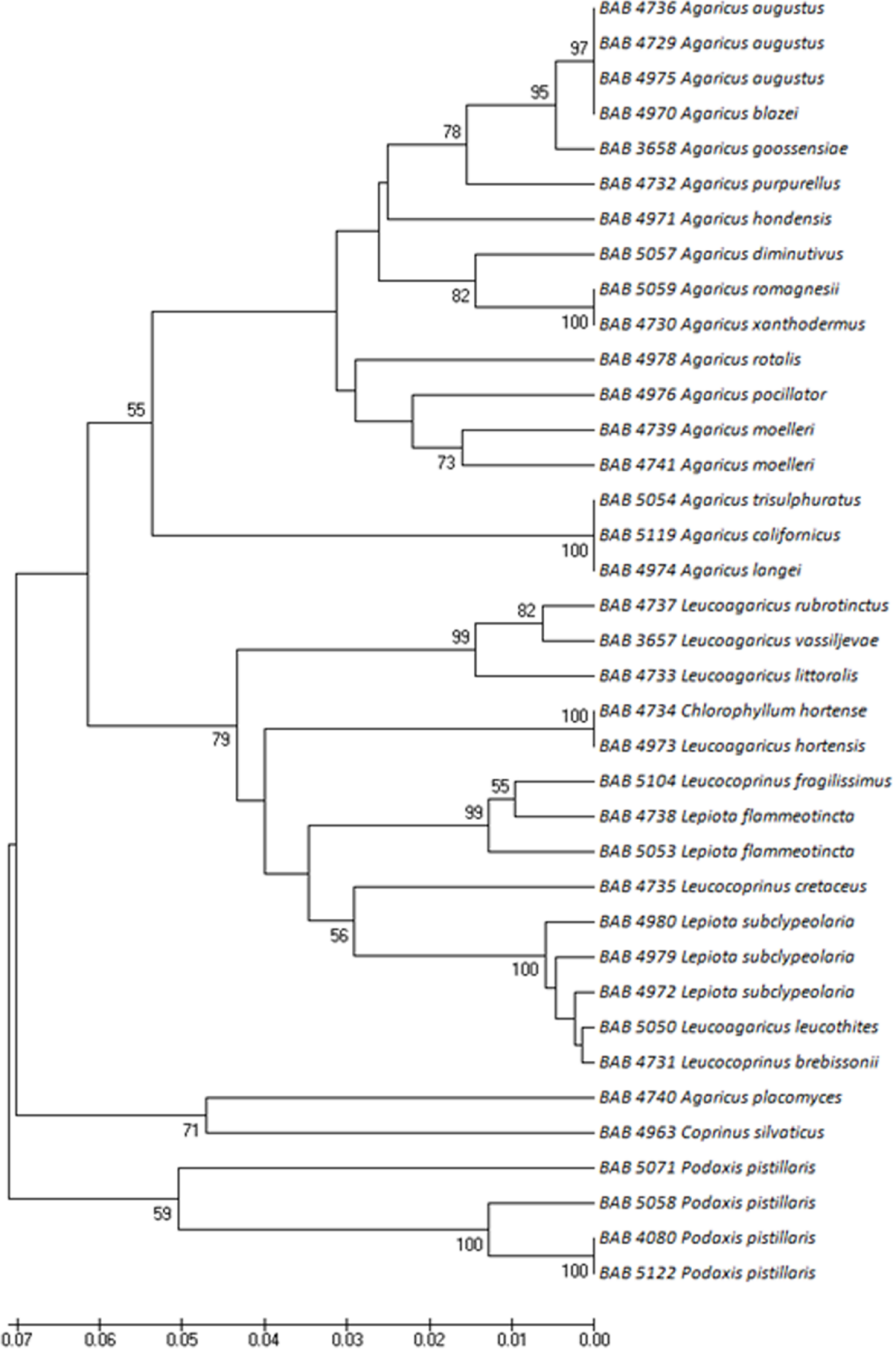
**ITS phylogeny of the Agaricaceae family inferred by UPGMA analysis—**Numbers at internodes refer to confidence estimates based on 100 bootstraps (only those > 50 are given).

37 individuals of Agaricaceae family including 23 unique species were identified. As shown in Fig 2, among the Agaricaceae family, *Podaxis pistillaris* (L.) Fr. forms the early rising monophyletic clade. The other genera including *Agaricus*, *Leucoagaricus*, *Leucocoprinus*, *Lepiota* and *Chlorophyllum* appear polyphyletic.

**Fig 3:**
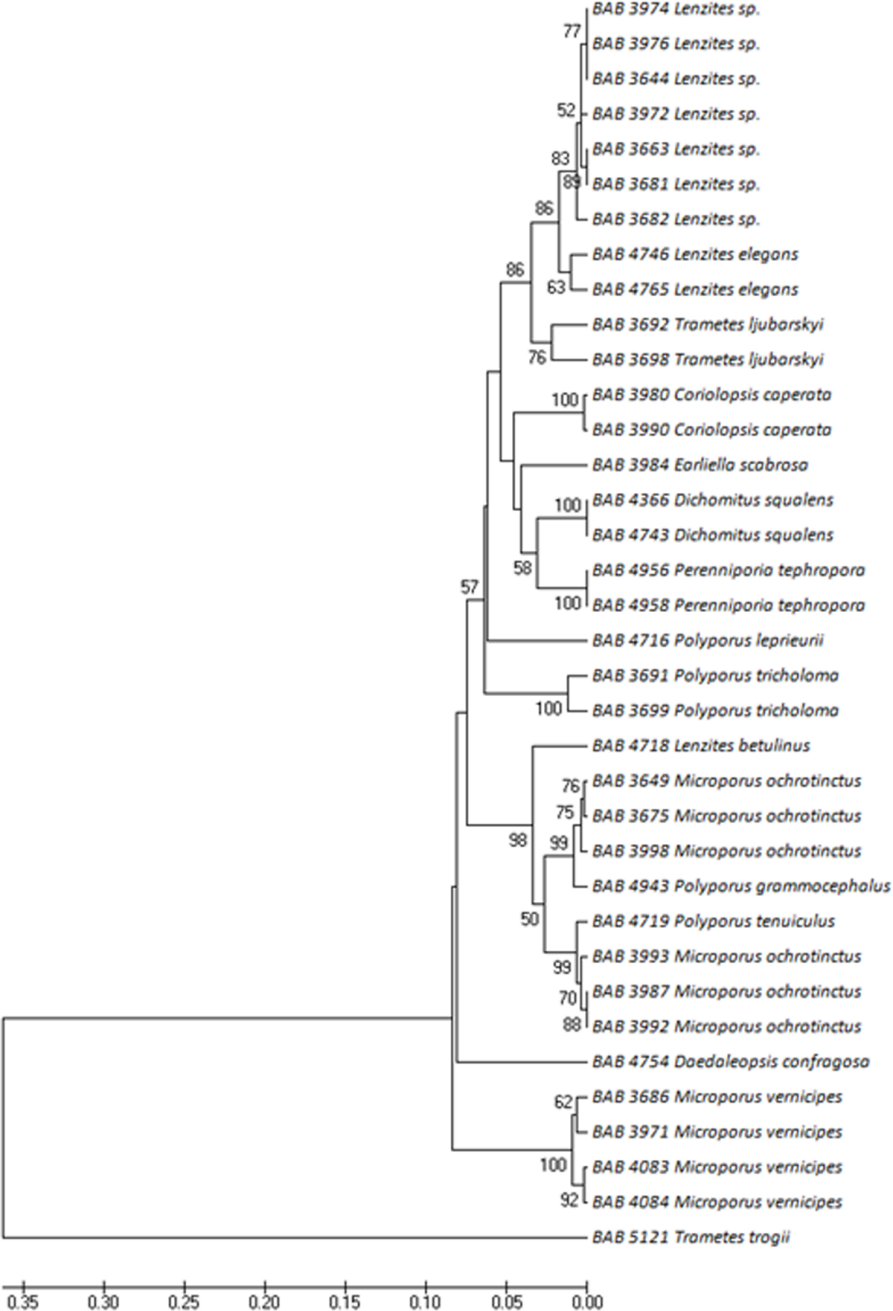
Phylogenetic tree of Polyporaceae–The phylogenetic tree produced from ITS sequence data with *Trametes trogii* forming the ancestor of the family. Bootstrap values greater than or equal to 50% are shown above their respective branches.

The other maximum number of 36 individuals was recorded from Polyporaceae family comprising of nine genera and 16 species. Fig 3 represents the phylogenetic relationship of the individuals from Polyporaceae family. Among the collected individuals, *Trametes trogii* Berk., Mitt. naturf. Ges. Bern is the early arising node forming the ancestor of this family from the present dataset. *Coriolopsis caperata* (Berk.) Murrill, *Dichomitus squalens* (P. Karst.) D.A. Reid *Perenniporia tephropora* (Mont.) Ryvarden, *Polyporus tricholoma* Mont. and *Microporus vernicipes* (Berk.) Imazeki having more than one individual collected from different locations form the monoclades individually with 100% bootstrap values.

**Fig 4:**
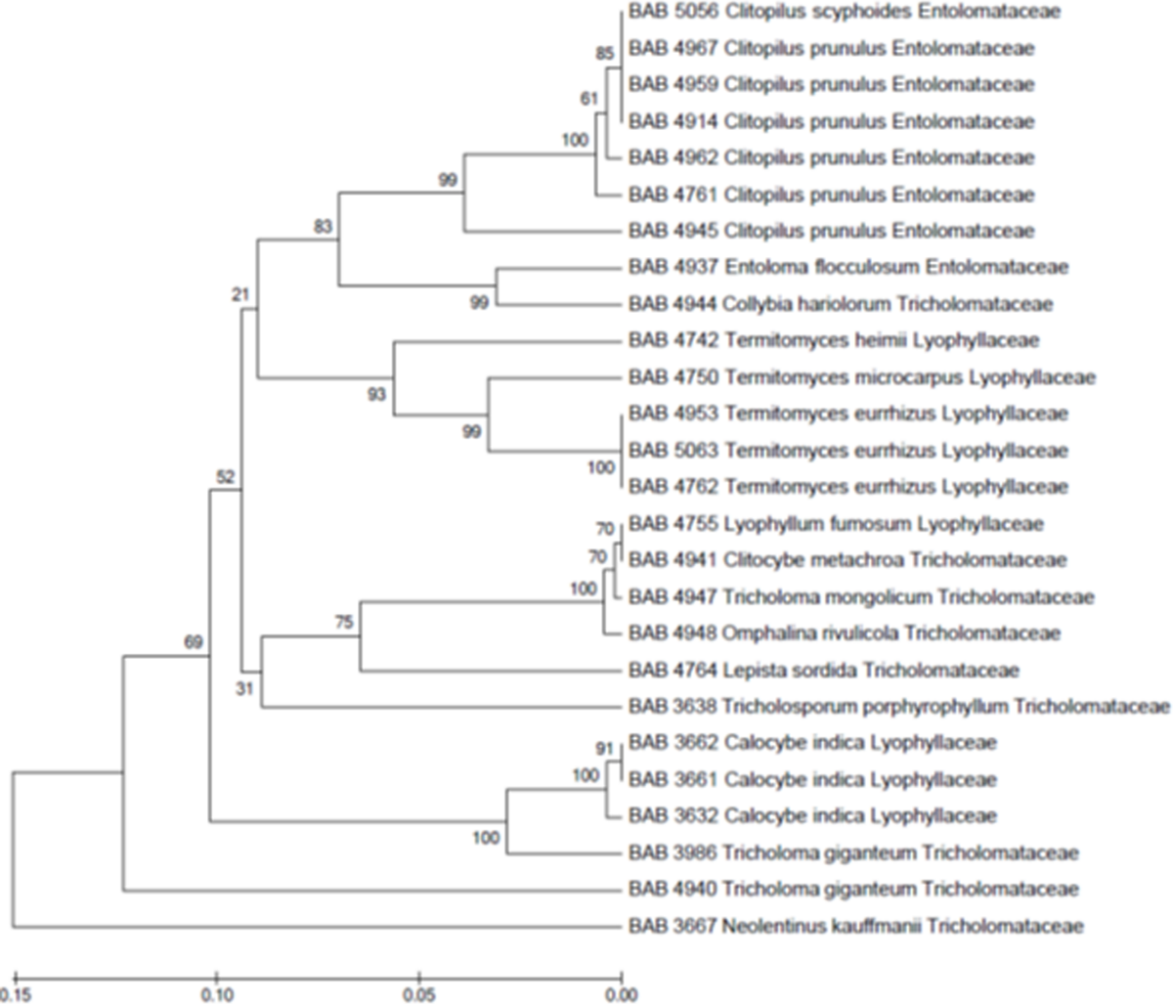
UPGMA phylogeny (ITS) of the Tricholomatoid clade–Phylogenetic tree showing relationships of different species belonging to a Tricholomatoid clade (Clade including Entolomataceae, Tricholomataceae and Lyophyllaceae families). Bootstrap values > 50 are shown at the branches.

In the present study nine mushrooms, each from Tricholomataceae and Lyophyllaceae families as well as 8 from Entolomataceae family were recorded. Fig 4 shows their evolutionary relationship in form of a phylogenetic tree. The phylogenetic analysis of the Tricholomatoid dataset revealed three monophyletic clades (*Clitopilus*, *Termitomyces* and *Calocybe*) that share a common ancestor from the Tricholomataceae family.

**Fig 5:**
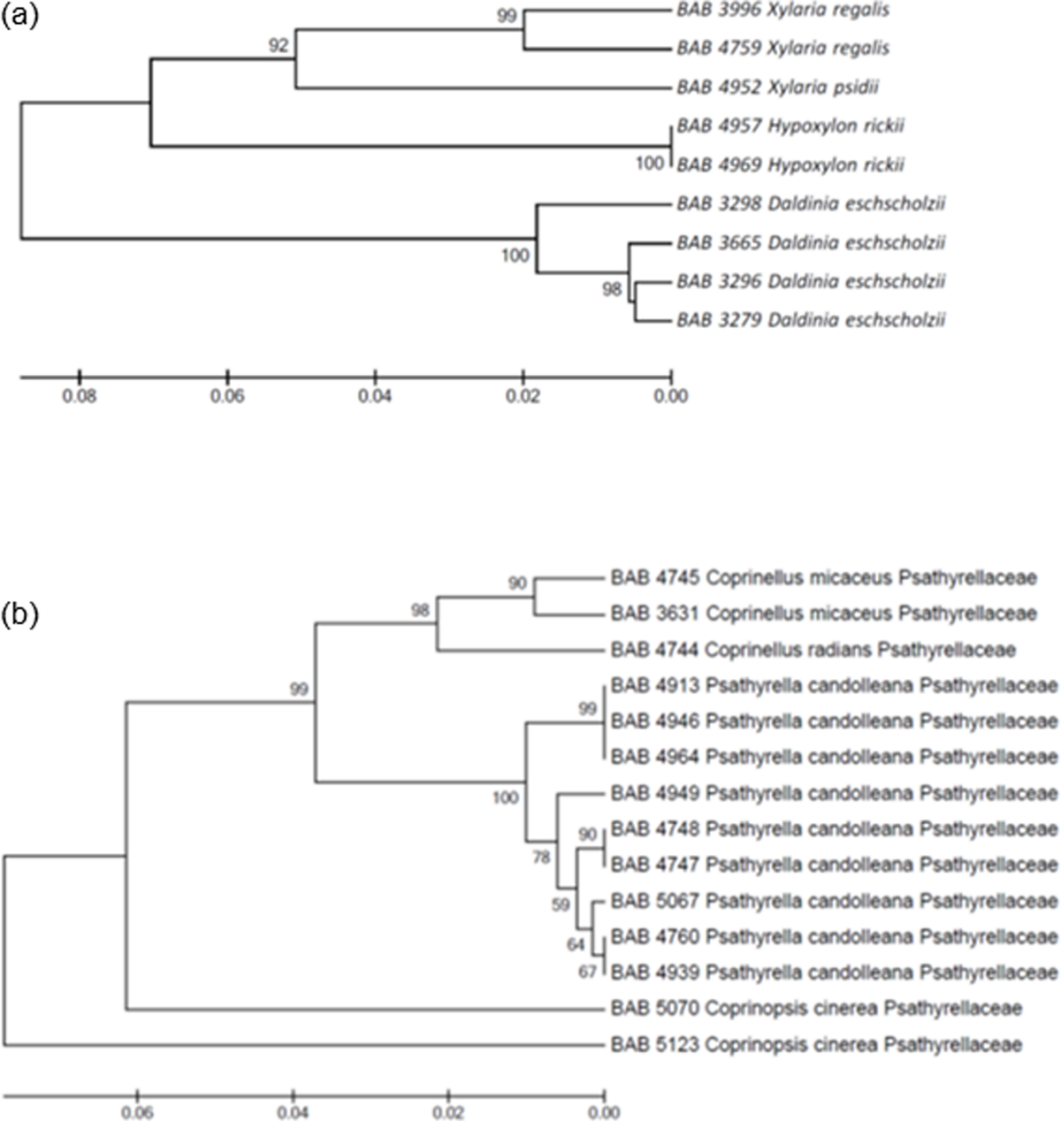
**Phylogenetic trees generated by UPGMA analysis from the ITS barcode sequences for (a) Xylariaceae and (b) Psathyrellaceae**.

The Xylariaceae family includes nine specimens belonging to three major genera; *Xylaria*, *Daldinia* and *Hypoxylon* and four species (Fig 5A). These genera clustered into two clades where Clade A consists of *Daldinia eschscholzii* (Ehrenb.) Rehm all collected from different locations of Gujarat. While clade B consists of *Hypoxylon rickii* Y.M. Ju & J.D. Rogers and *Xylaria psidii* Rogers et Hemmes and *Xylaria regalis* Cooke. Within the Psathyrellaceae family, *Coprinopsis cineria* (Schaeff.) Redhead, Vilgalys & Moncalvo collected from Kutch forms the early node (Fig 5B). With a variation in sequence, the same species collected from Gandhinagar appears on the other clade, giving rise to two subclades with 99% bootstrap value, one of genus *Psathyrella* and other of *Coprinellus*.

**Fig 6:**
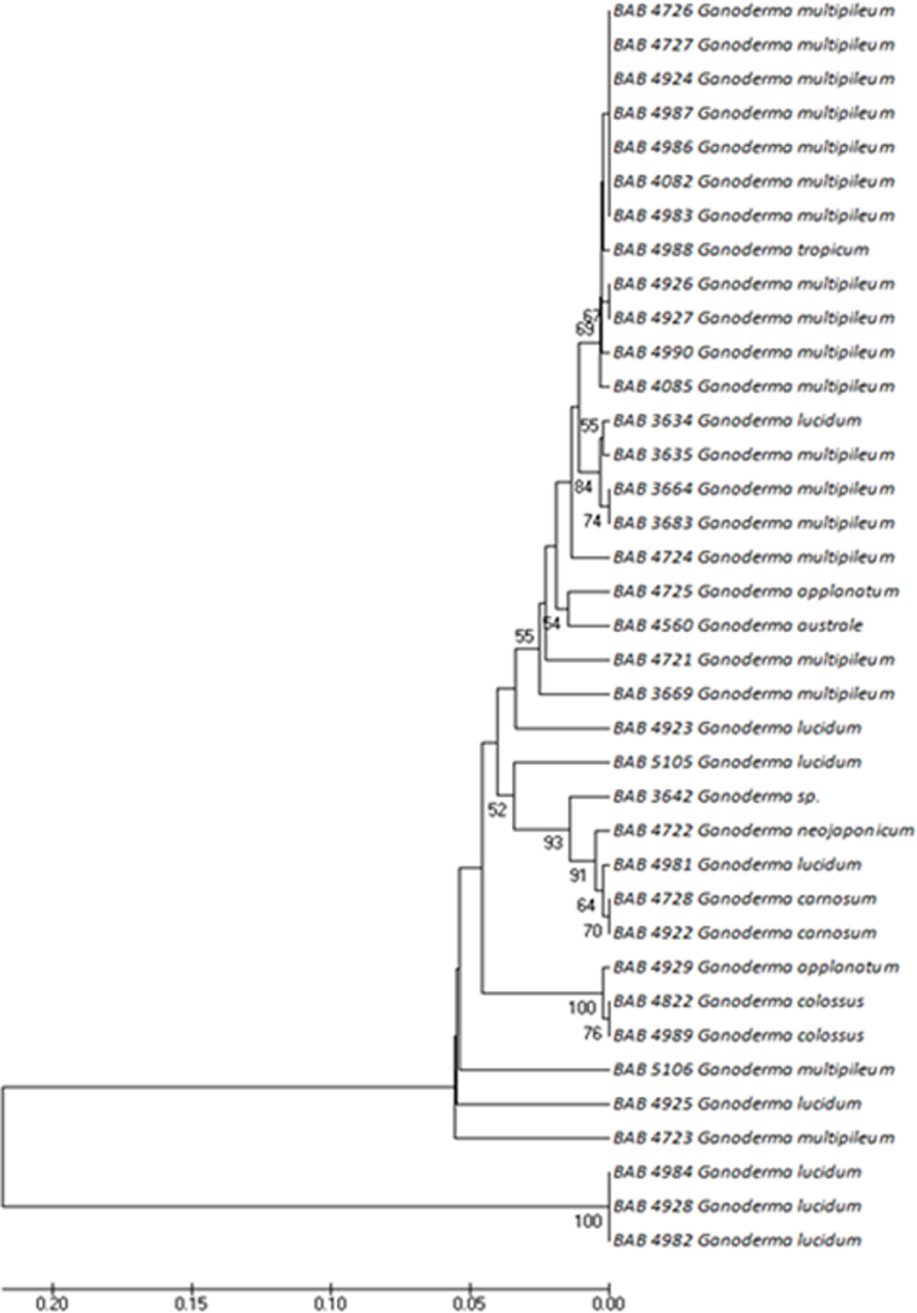
Phylogenetic tree generated by UPGMA analysis from the ITS barcode sequences for Ganodermataceae.

**Fig 7:**
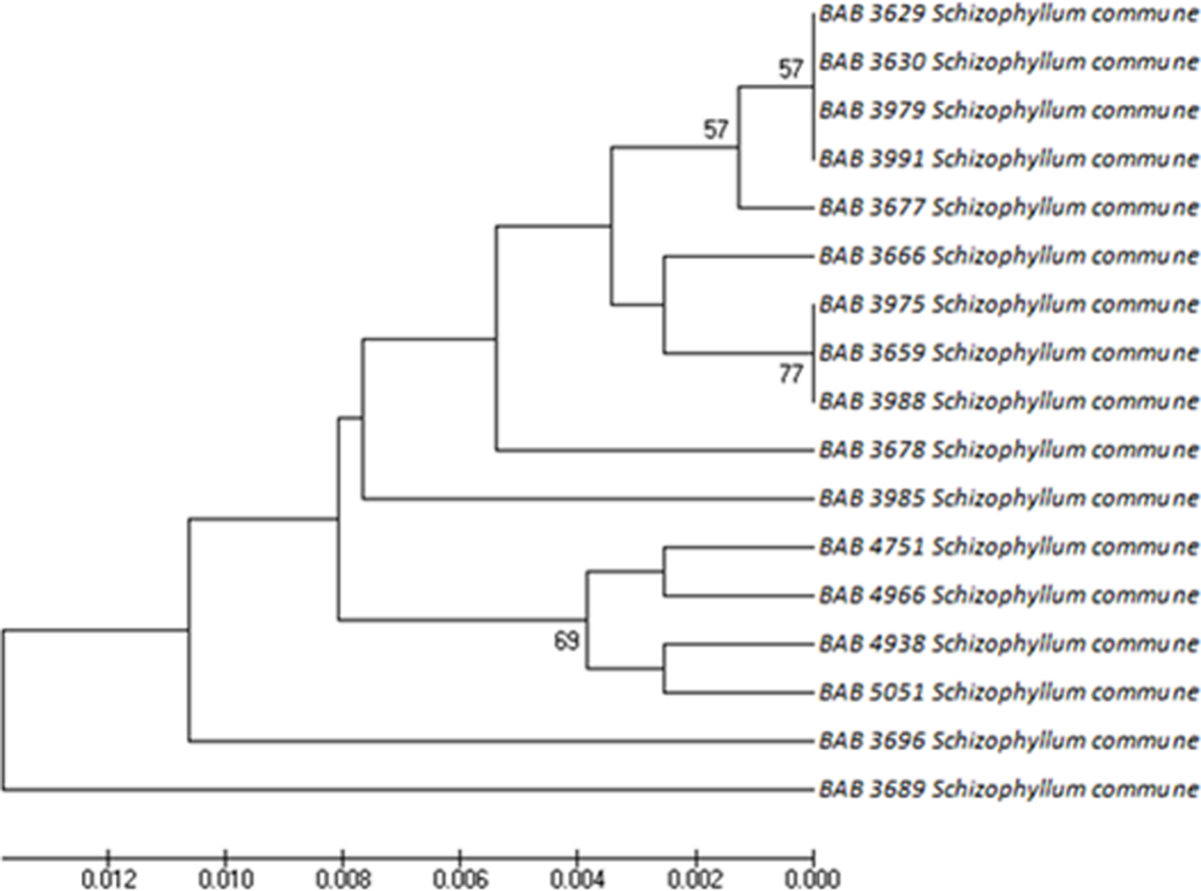
Phylogenetic trees generated by UPGMA analysis from the ITS barcode sequences for Schizophyllaceae.

**Fig 8:**
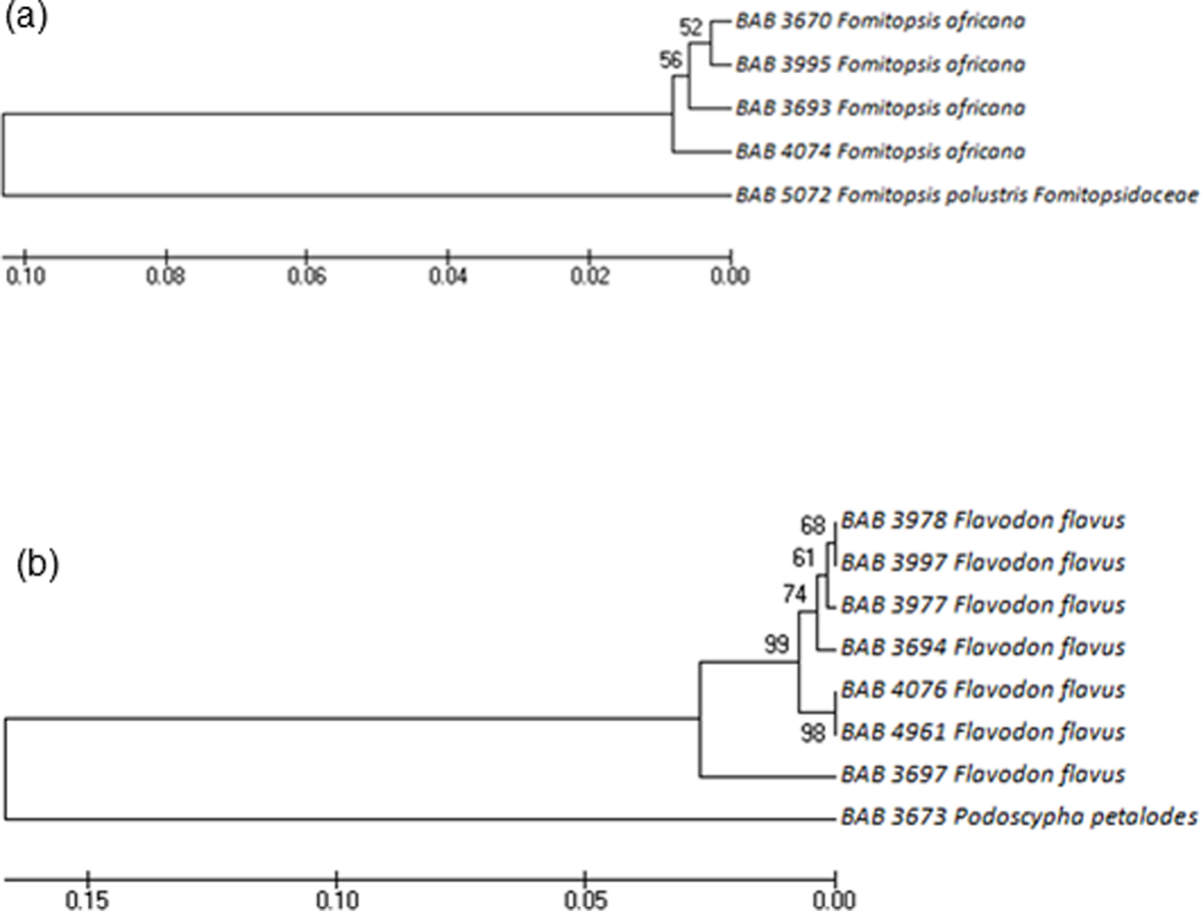
**Phylogenetic trees generated by UPGMA analysis from the ITS barcode sequences for (a) Fomitopsidaceae and (b) Meruliaceae**.

Figs 6–8 represents the evolutionary pattern of similar species with multiple specimens that belonged to (a) Ganodermataceae, (b) Schizophyllaceae, (c) Fomitopsidaceae and (d) Meruliaceae families collected from different locations of Gujarat. The Ganodermataceae family was recorded with 37 individuals represented by multiple specimens of *G*. *multipileum* and *G*. *lucidum*spread all over Gujarat. The collection also included species like *G*. *colossus*, *G*. *applanatum*, *G*. *carnosum*, *G*. *neojaponicum*, *G*. *austral* and *G*. *tropicum*.

*Schizophyllum commune* of Schizophyllaceae family can be considered as one of the common species of Gujarat as it was the only species of the family recorded from 8 out of 10 regions of Gujarat. The phylogenetic study of *Schizophyllum commune* showed the variation among the sequences recorded from Thol, Godhra and Anatarsuba be the newly evolved species among all the other geographic locations surveyed in this study.

Within Fomitopsidaceae, two species *Fomitopsis africana* Mossebo & Ryvarden and *Fomitopsis palustris* (Berk. & M. A. Curtis) Gilb. & Ryvarden were recorded. The evolutionary study revealed that *F*. *africana* collected from 4 different forest regions showed variations in the sequences collected from Godhra region to Polo forest resulting sp. collected from Godhra forms the ancestor and one collected from Polo forest the newly evolved sp.

Meruliaceae family was recorded with 8 individuals comprising of a single specimen of *Podoscypha petalodes* (Berk.) Pat. and multiple specimens of *Flavodon* flavus (Klotzsch) Ryvarden, *Podoscypha petalodes* (Berk.) Pat. forms the ancestor of the Meruliaceae family giving rise to *Flavodon flavus*. Among the multiple collections of *Flavodon flavus*, the one collected from Gandhinagar forms the early node. While the same species collected from Godhra and Junagadh appear to be newly evolved species.

### Barcode gap analysis and data assessment

**Fig 9:**
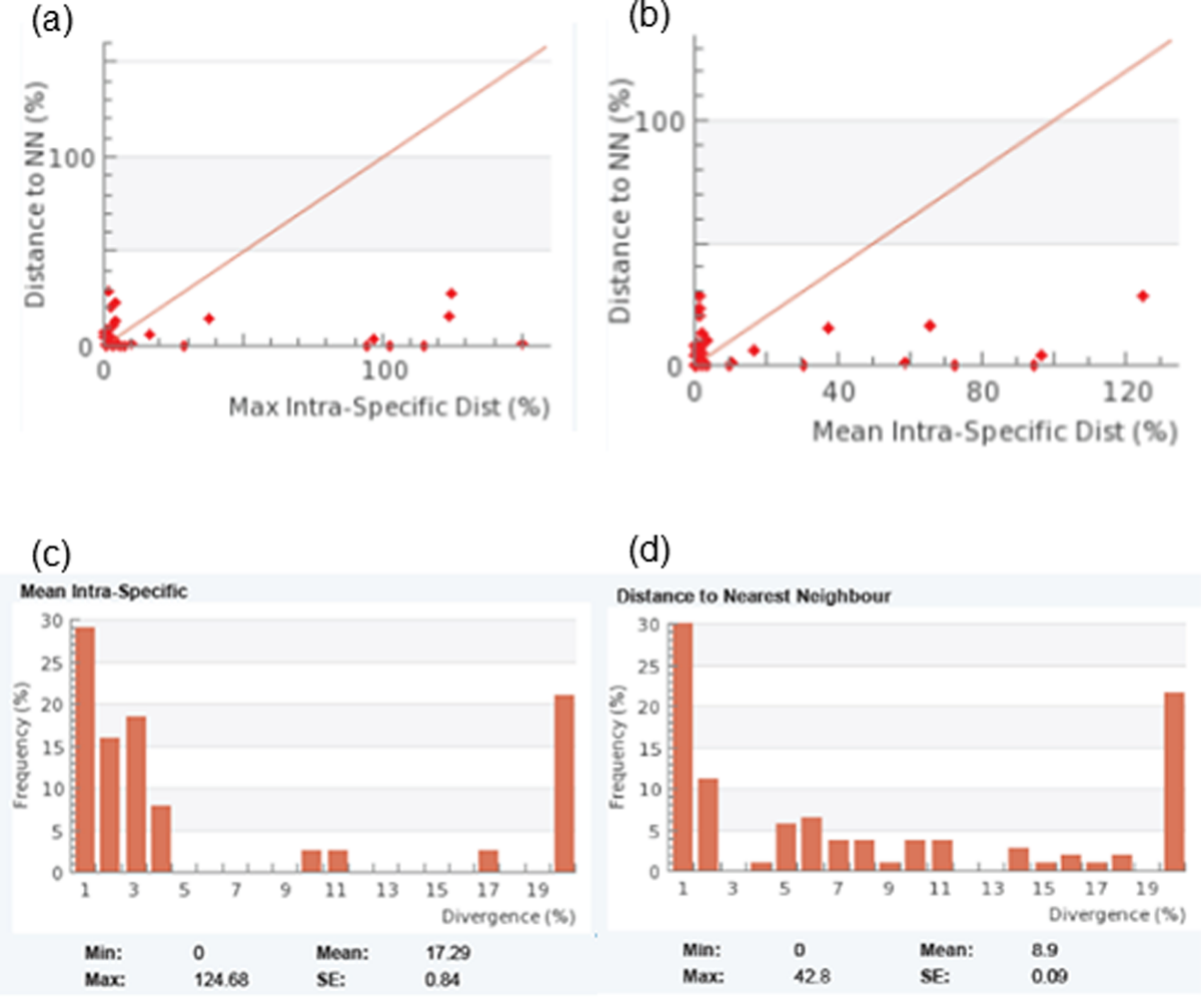
**Barcode gap analysis–scatterplots showing overlapping of (a) max Intra-specific vs nearest neighbor (b) mean Intra-specific vs nearest neighbor; histogram plots showing distribution of (c) mean Intra-specific distances for each species and (d) frequency histogram of distance to nearest neighbor**.

The Barcode gap analysis revealed several species having considerable (>2%) intraspecific variation. The existence and magnitude of the Barcode gap are confirmed in Fig 9 of two scatterplots showing the overlapping of the maximum and mean intra-specific distances against the nearest neighbor distances. The distribution of mean intra-specific distances for each species ranged from 0 to 124.64% with a mean of 17.29% (SE = 0.84) while the nearest neighbor distances ranged from 0 to 42.8% with a mean of 8.9% (SE = 0.09). The maximum intraspecific variation exceeded 2% in 22 species and 44 species showed less than 2% distance to the nearest neighbor. The overall GC content was 46.02% (SE = 0.2). The mean GC content at codon positions 1, 2 and 3 were 5.99% (SE = 0.29), 45.97% (SE = 0.28) and 46.09% (SE = 0.28) respectively (Table 3).

**Table 3:** Number of partial characters for 225 sequences grouped by families.

Grouping by Family		
Group name (# Sequences)	# Diagnostic characters	# Partial characters
Agaricaceae (37)	0	0
Lyophyllaceae (9)	0	1
Tricholomataceae (9)	0	0
Entolomataceae (8)	0	126
Psathyrellaceae (14)	0	2
Polyporaceae (36)	0	0
Xylariaceae (9)	4	12
Meruliaceae (8)	0	13
Fomitopsidaceae (5)	0	13
Hymenochaetaceae (14)	0	0
Ganodermataceae (37)	0	0
Phallaceae (3)	14	91
Schizophyllaceae (17)	15	58

Table 3 shows the study of all the sequences for consensus bases with the distribution of diagnostic and partial characters for 225 sequences grouped according to their families. Fifteen diagnostic characters i.e. the nucleotides found only in that particular group were recognized for Schizophyllaceae family that included 17 sequences. In addition, 126 partial characters were recognized for Entolomataceae family that included 8 sequences.

### Bayesian estimation of divergence time

**Fig 10:**
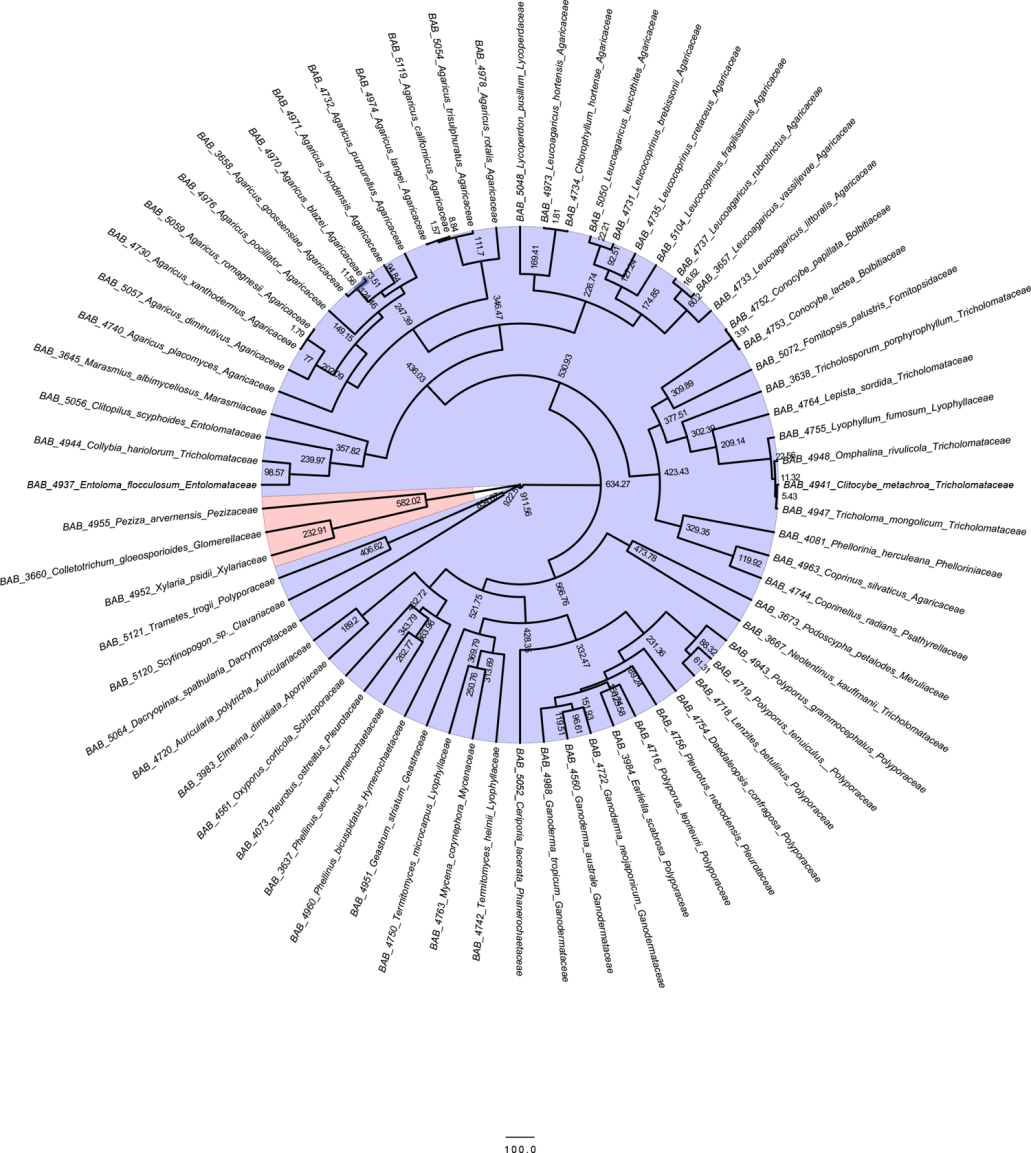
**Chronogram and estimated divergence times of 68 unique mushrooms collected from Gujarat using ITS dataset–**The chronogram is obtained by using the Ascomycota–Basidiomycota divergence time of 582 Mya as the calibration point.

In our study, 68 species having single ITS barcodes were taken for Divergence time estimation. Chronographic analysis using 582 Mya as the calibration point for the divergence between Ascomycota and Basidiomycota is shown in Fig 10. The estimated divergence times for different singleton species are summarized in Table 4. The study showed that *Dacryopinax spathularia* (Schwein.) G. W. Martin is the early ancestor, which evolved some 854.67 million years ago. Our analyses suggested that the ancient divergence of the Hymenochaetales from the Agaricomycetes was during the Silurian period (416.0–443.7 Mya) and the family Schizoporaceae diverged from Hymenochaetales before 343.79 million years. Ganodermataceae being one of the important families of mushrooms evolved from Polyporales during the Jurassic period (145.5–199.6 Mya) while another important family Agaricaceae evolved in the Silurian period. In addition, *Agaricus langei* (F.H.Møller) F.H.Møller and *Agaricus californicus* Peck evolved from Agaricaceae family with least divergence time of 1.56 Mya. Divergence time at genus level for *Leucoagaricus hortensis* (Murrill) Pegler and *Chlorophyllum hortense* (Murrill) Vellinga from the family Agaricaceae was observed at 1.81 million years ago. *Xylaria psidii* Rogers et Hemmes and *Colletotrichum gloeosporioides* (Penz.) Penz. & Sacc. diverged from *Peziza arvernensis* Boud. before 232.91 million years within Ascomycota phylum.

**Table 4:** Estimated divergence time of main nodes.

Main nodes	Mean (Mya)	95% HPD (Mya)
Sordariomycetes/Pezizomycetes	582.021	580.06–583.93
Hymenochaetales/Agaricomycetes	432.724	329.55–536.39
Ganodermataceae/Polyporales	151.928	109.02–196.23
Bolbitiaceae/Fomitopsideaceae	309.892	229.81–396.82
*Agaricus*sp./Agaricaceae	346.466	260.57–442.18
*Dacryopinax spathularia*	854.671	677.72–1057.38
*Trametes trogii*	406.624	295–541.64

## Discussion

The present study clearly attests the efficiency of ITS to discriminate various fungal taxa. The study further estimates the divergence times of the taxa taken under study. Among the phylogenetic reconstruction methods used distance based methods (those that proceed algorithmically through distances), UPGMA gave the best resolution in tree topology. This result is supported by the reports of Wang et al. [33], Yokoyama et al. [34].

The phylogenetic tree of Agaricaceae reveals monophyletic clades for *Agaricus and Podaxis* with *Podaxis* forming the ancestral clade (Fig 2).Monophyletic clade of *Podaxis* indicates a clear advantage of using ITS as a marker as *Podaxis* has been rather difficult to differentiate from *Coprinus* due to morphological similarities [35]. Further, *Podaxis* is a secotioid fungus and the results support the hypothesis that gasteroid forms evolved from secotioid forms [36]. The gasteroid forms have an advantage of spore dispersal in air as against a requirement of rodents/ insects for spore dispersal in secotioid form.

As expected the phylogenetic analysis of Polyporaceae family revealed a close relationship between *Lenzites*, *Trametes*, *Earliella*, *Dichotomus* and *Pereniporia* as previously described by Ko [37], Garcia-Sandoval *et al*. [38] and Rajchenberg [39] reconfirming the polyphyletic nature of Polyporaceae members. It thus appears that simple ITS based phylogenetic analysis may not clearly differentiate the taxa of Polyporaceae members. The present study also indicates that the morphological features, such as lamellate or pored hymenophore and colour of the hyphae should not be neglected during taxonomic identification of the Polyporaceae members. Further the genera *Trametes* and *Lenzites* showed a very close association. Infact there are reports where *Trametes* and *Lenzites* have been used as synonyms for example: *Lenzites elegans* is synonymous with *Trametes elegans* and *Lenzites betulinus* with *Trametes betulina*. Welti *et al* [40] suggests that *Trametes* may be enlarged to keep *Lenzites*. Thus the study supports the enlarged *Trametes* concept of Kavina and Pilat [41].

The tricholomatoid clade shows a clear demarcation for Entolomataceae. However, Lyophyllaceae and Tricholomataceae could not be distinguished clearly. *Calocybe indica* appear closer to *Tricholoma giganteum (Macrocybe gigantea*) where both these mushrooms are generally considered very close together. However, the *Tricholoma giganteum* clade is different from *Calocybe* clade and finds support from the work of Razaq *et al* [42]. Clearly defined clades of *Xylaria* and *Hypoxylon*find support from the reports of Tang *et al*. [43]. Phylogenetically, *Daldinia* is clearly distinct from *Xylaria* and *Hypoxylon* which is supported by morphological distinction as well.

Among all the fungal taxa studied, *Ganoderma* and *Schizophyllum* were most widely distributed. Moreover, *Ganoderma* could not be clearly differentiated from the phylogenetic studies and are not monophyletic. It appears that distribution studies of the mushrooms in Gujarat, India showed omnipresence of *Ganoderma* and *Schizophyllum* where both of them do not appear monophyletic as per the present study. *Ganoderma lucidum* and *Ganoderma multipileum* which are used as synonyms [44, 45] do not appear monophyletic species as reported by Monclavo *et al* [46]. Both these species of *Ganoderma* differed in morphology, which varied from red cap, to different shades of brown color and a dirty white cap as well. Thus it appears that ITS may not be alone efficient in discriminating the species.

Several studies have been undertaken to determine the divergence time of fungal taxa [16 – 23] using different strategies. In the present study calibration point (i.e., 582 million yr ago, Mya) based on a 400 million year old fossil *Paleopyrenomycites devonicus* is considered [30–31]. Dacrymycetes, one of the early-diverging wood decomposers in Basidiomycota, sister to Agaricomycetes appeared to have evolved in the Neoproterozoic Era, the last era of the Precambrian Supereon and the Proterozoic Eon. It is believed that eukaryotic multicellularity evolved during this era. Another important result during divergence time estimation is the divergence of the Hymenochaetales from the Agaricomycetes during the Silurian period. Hymenochaetales is among one of the three orders Russulales, Boletales, and Hymenochaetales that evolved independently from Agaricomycetes. It is believed that wood decay evolved during this period [33] with the diversification of Agaricomycetes.

Our study confirms that increasing the use of DNA barcoding can overcome the limitations of morphology based identifications and help identify previously unidentified species by documenting the diversity of ITS sequences within currently recognized species. This use of molecular data should be complementary to morphological analysis in such endeavors, and the establishment of reliable global ITS barcode databases for mushrooms will help to be able to accurately identify any mushroom at any stage of the life cycle or even from small pieces of tissue.

Despite increasing interest in dating the origin and diversification of different groups of fungi, we still lack detail information on the evolutionary history of a major phylum of fungi from Gujarat region. Our findings suggest that *Dacryopinax spathularia* is the early evolved diverged species among the unique species of Gujarat. We also provide estimates for the origin and diversification dates for many clades in the Basidiomycota using ITS dataset, which previously had not been dated from India, including major orders, families as well as certain species.

## Supporting information

S1 Fig Photographs for all the collected mushroom specimens, in the case of multiple specimens, representative images are shown. (PDF)

S2 Fig Phylogenetic trees generated using Maximum likelihood and Maximum parsimony analysis for different families in the study. (PDF)

S1 Table Process IDs of specimen collected and accession numbers. (DOCX)
