# Supplementary material for: Molecular survey of basidiomycetes and divergence time estimation: An Indian perspective
Source: PLoS One. 2018 May 17;13(5):e0197306. doi: 10.1371/journal.pone.0197306 (PMC5957343; doi:10.1371/journal.pone.0197306)
Supplement: S1 Fig — (PDF) [file pone.0197306.s001.pdf]

Fig S1: Photographs for all the collected mushroom specimens, in the case of multiple specimens, representative images are shown

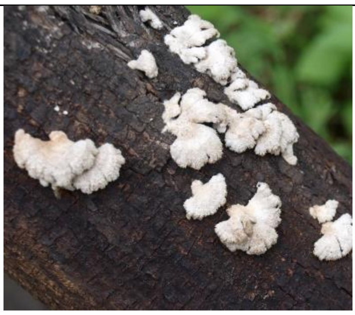<br>BAB 3629<br>*Schizophyllum commune*	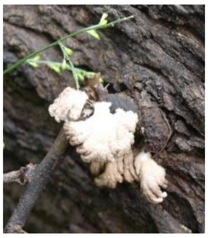<br>BAB 3630<br>*Schizophyllum commune*	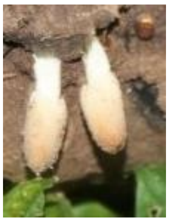<br>BAB 3631<br>*Coprinellus micaceus*
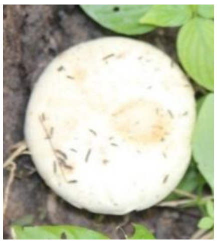<br>BAB 3632<br>*Calocybe indica*	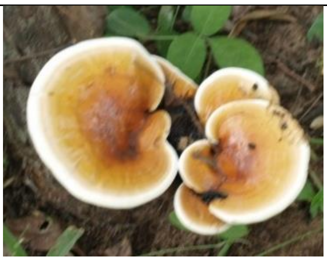<br>BAB 3634<br>*Ganoderma lucidum*	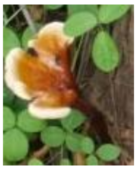<br>BAB 3635<br>*Ganoderma multipileum*
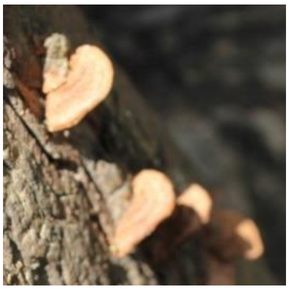<br>BAB 3637<br>*Phellinus senex*	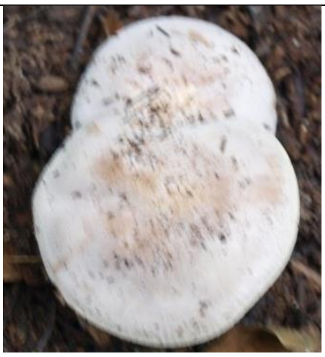<br>BAB 3638<br>*Tricholosporum porphyrophyllum*	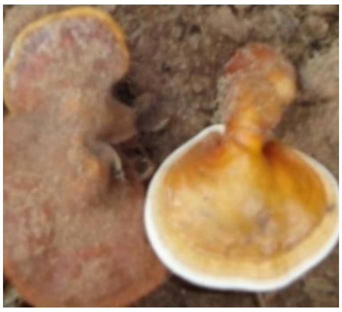<br>BAB 3642<br>*Ganoderma sp.*
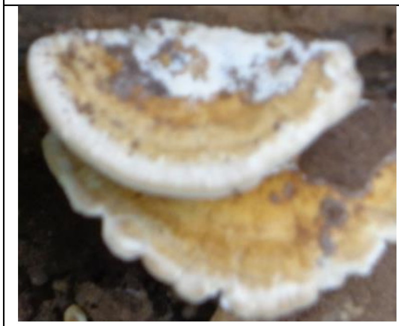<br>BAB 3644<br>*Lenzites sp.*	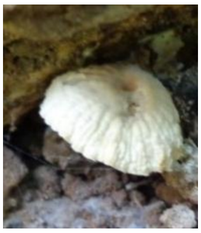<br>BAB 3645<br>*Marasmius albimyceliosus*	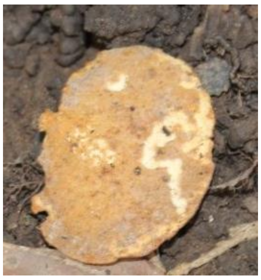<br>BAB 3649<br>*Microporus ochrotinctus*

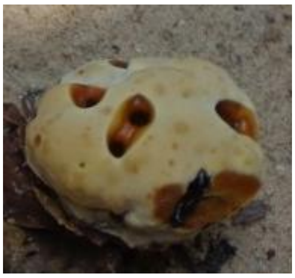<br>BAB 3652<br>*Inonotus porrectus*	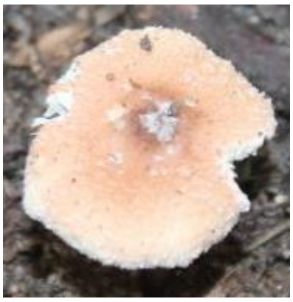<br>BAB 3657<br>*Leucoagaricus vassiljevae*	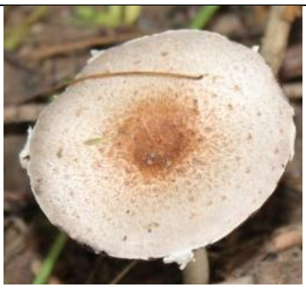<br>BAB 3658<br>*Agaricus goossensiae*
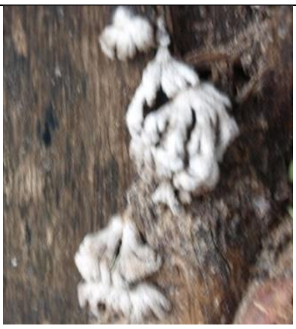<br>BAB 3659<br>*Schizophyllum commune*	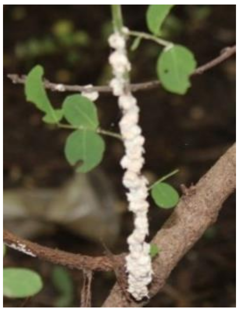<br>BAB 3660<br>*Colletotrichum gloeosporioides*	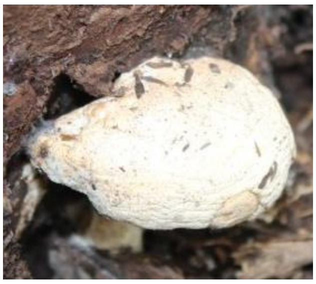<br>BAB 3661<br>*Calocybe indica*
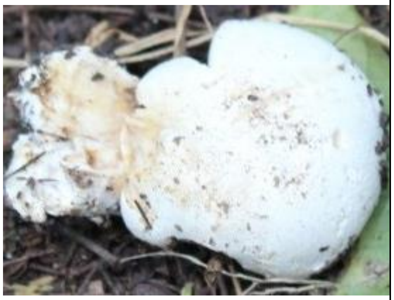<br>BAB 3662<br>*Calocybe indica*	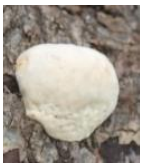<br>BAB 3663<br>*Lenzites sp.*	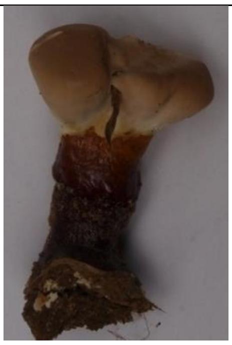<br>BAB 3664<br>*Ganoderma multipileum*
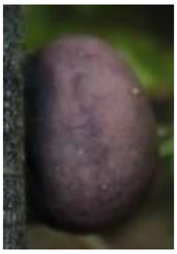<br>BAB 3665<br>*Daldinia eschscholzii*	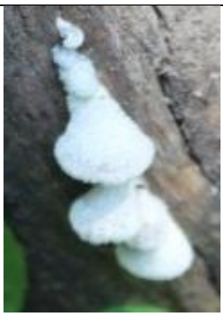<br>BAB 3666<br>*Schizophyllum commune*	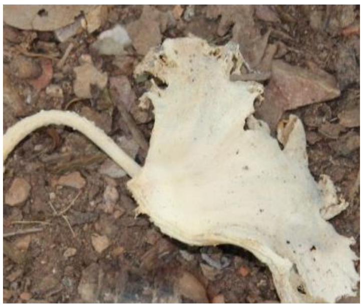<br>BAB 3667<br>*Neolentinus kauffmanii*

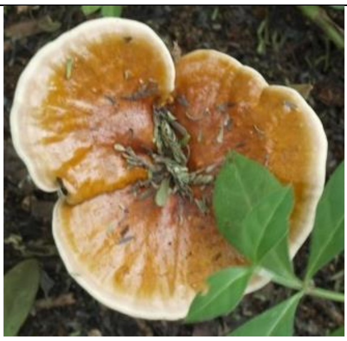<br>BAB 3669<br>*Ganoderma multipileum*	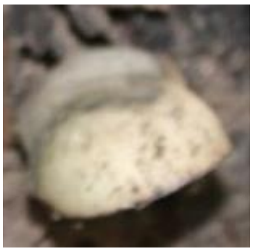<br>BAB 3670<br>*Fomitopsis africana*	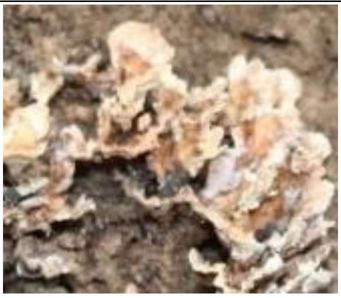<br>BAB 3673<br>*Podoscypha petalodes*
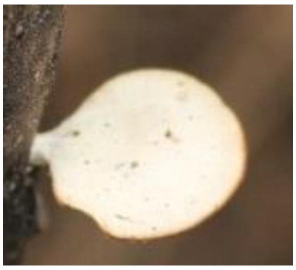<br>BAB 3675<br>*Microporus ochrotinctus*	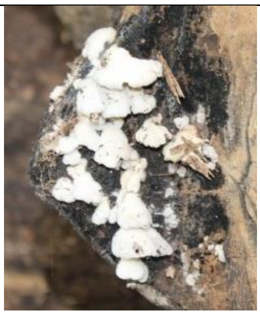<br>BAB 3677<br>*Schizophyllum commune*	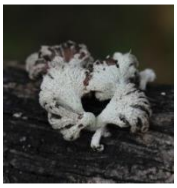<br>BAB 3678<br>*Schizophyllum commune*
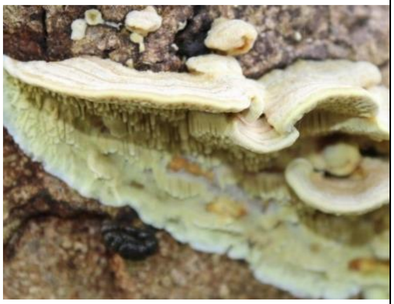<br>BAB 3681<br>*Lenzites sp.*	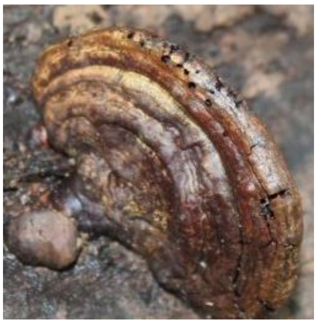<br>BAB 3682<br>*Lenzites sp.*	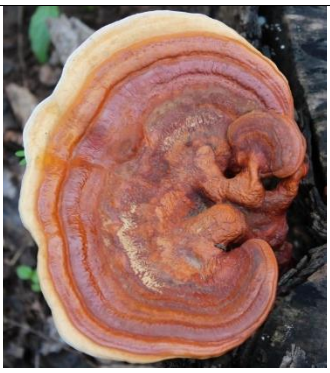<br>BAB 3683<br>*Ganoderma multipileum*
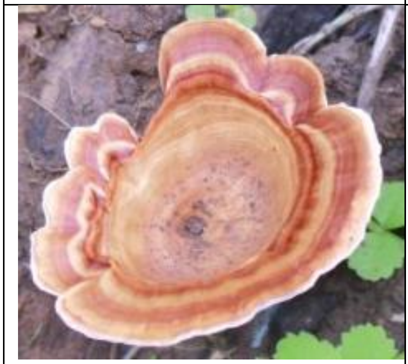<br>BAB 3686<br>*Microporus vernicipes*	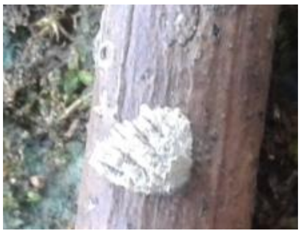<br>BAB 3689<br>*Schizophyllum commune*	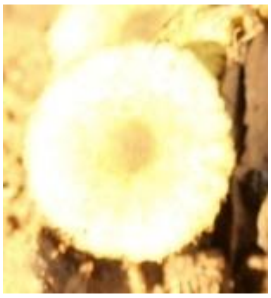<br>BAB 3691<br>*Polyporus tricholoma*

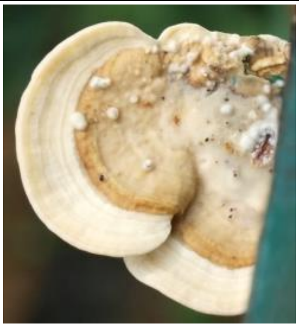 BAB 3692 *Trametes ljubarskyi*	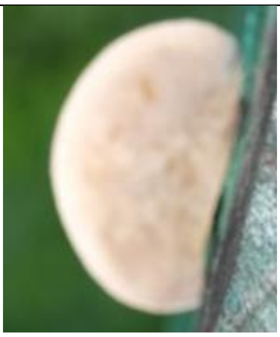 BAB 3693 *Fomitopsis africana*	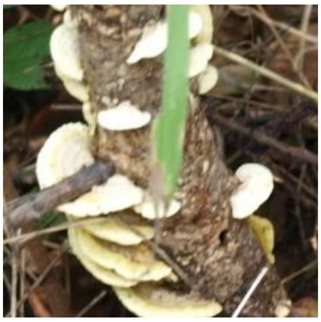 BAB 3694 *Flavodon flavus*
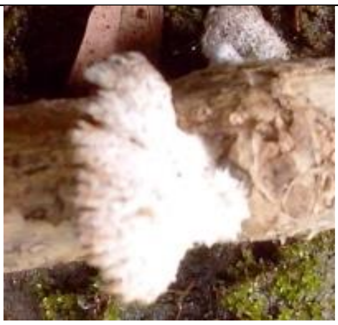 BAB 3696 *Schizophyllum commune*	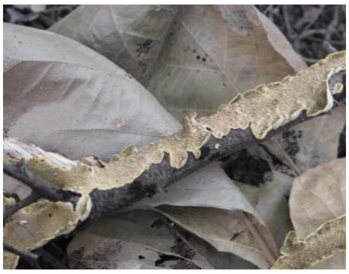 BAB 3697 *Flavodon flavus*	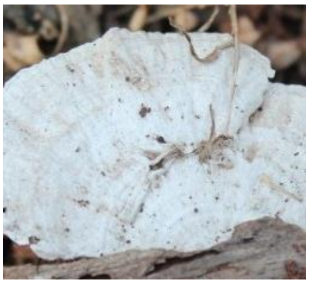 BAB 3698 *Trametes ljubarskyi*
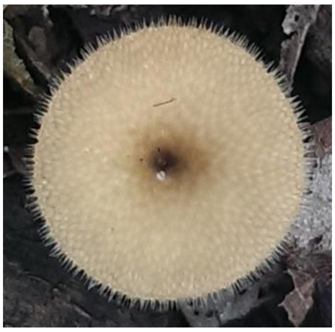 BAB 3699 *Polyporus tricholoma*	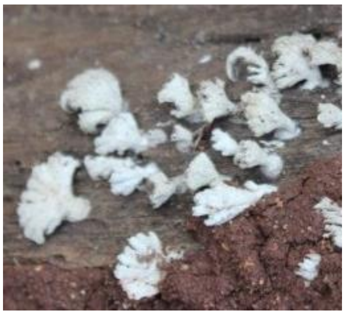 BAB 3975 *Schizophyllum commune*	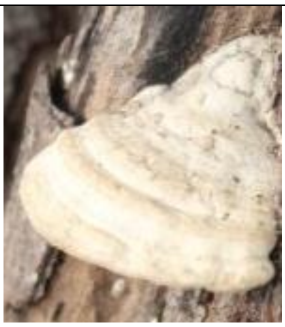 BAB 3976 *Lenzites sp.*
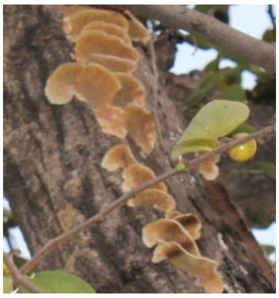 BAB 3977 *Flavodon flavus*	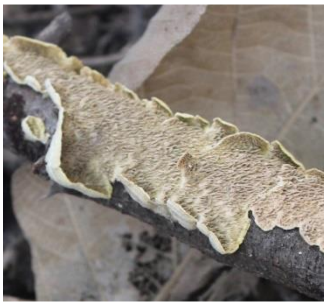 BAB 3978 *Flavodon flavus*	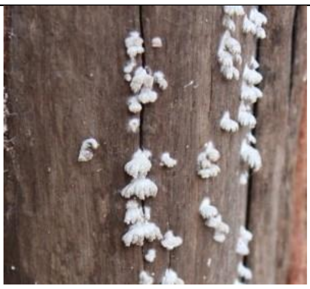 BAB 3979 *Schizophyllum commune*

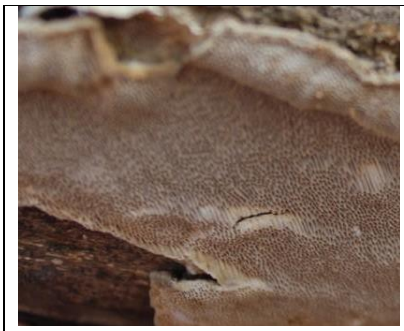

BAB 3980
Coriolopsis caperata

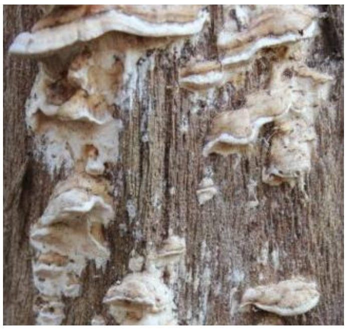

BAB 3983
Elmerina dimidiata

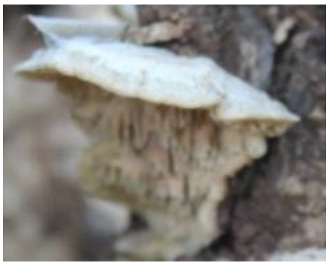

BAB 3974
Lenzites sp.

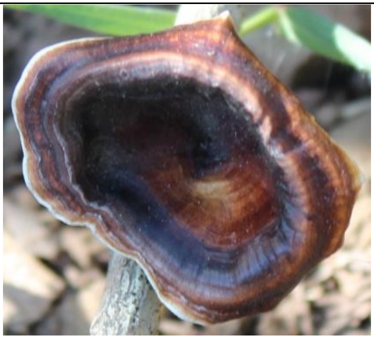

BAB 3971
Microporus vernicipes

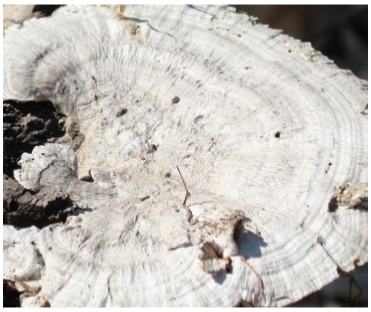

BAB 3972
Lenzites sp.

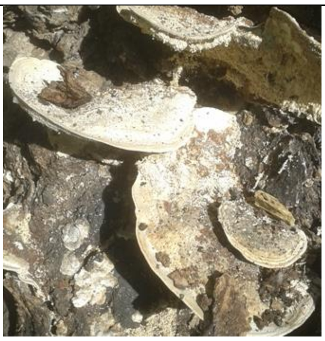

BAB 3984
Earliella scabrosa

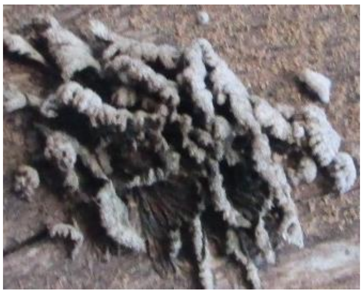

BAB 3985
Schizophyllum commune

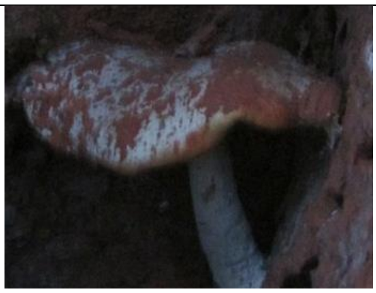

BAB 3986
Tricholoma giganteum

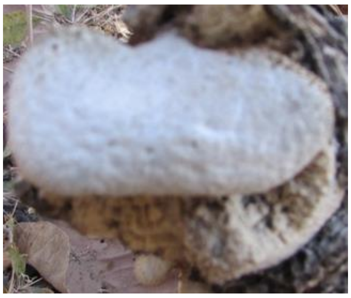

BAB 3987
Microporus ochrotinctus

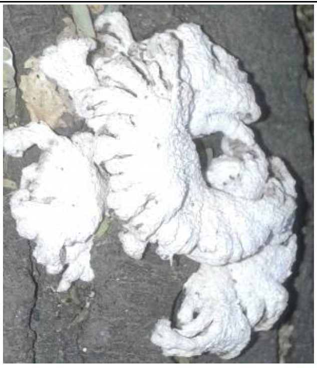

BAB 3988
Schizophyllum commune

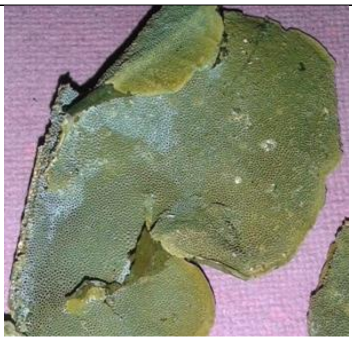

BAB 3990
Coriolopsis caperata

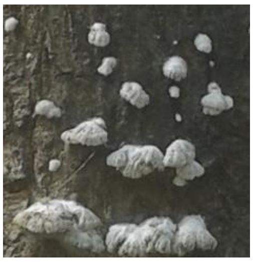

BAB 3991
Schizophyllum commune

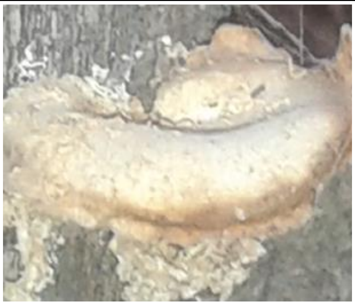<br>BAB 3992<br>*Microporus ochrotinctus*	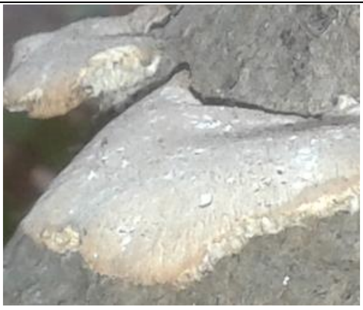<br>BAB 3993<br>*Microporus ochrotinctus*	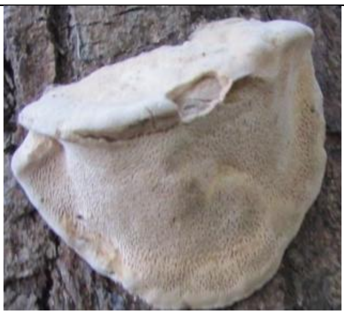<br>BAB 3995<br>*Fomitopsis africana*
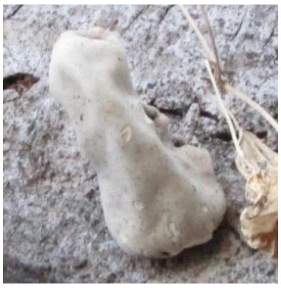<br>BAB 3996<br>*Xylaria regalis*	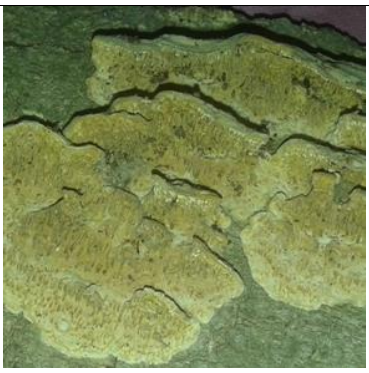<br>BAB 3997<br>*Flavodon flavus*	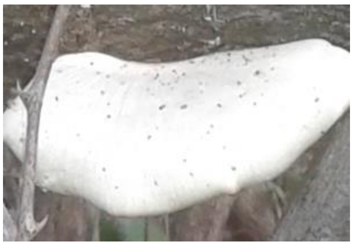<br>BAB 3998<br>*Microporus ochrotinctus*
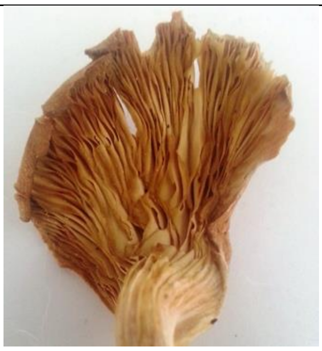<br>BAB 4073<br>*Pleurotus ostreatus*	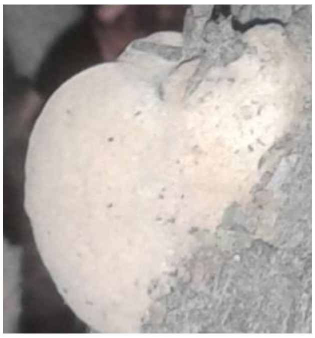<br>BAB 4074<br>*Fomitopsis africana*	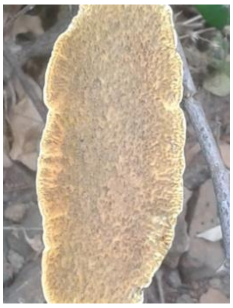<br>BAB 4076<br>*Flavodon flavus*
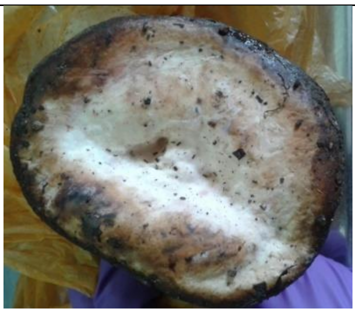<br>BAB 4077<br>*Amylosporus campbellii*	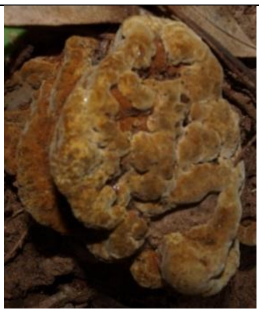<br>BAB 4079<br>*Fulvifomes fastuosus*	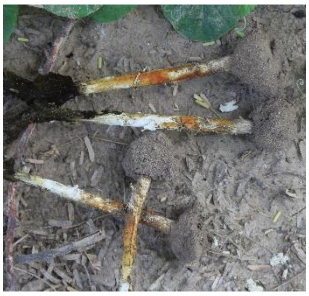<br>BAB 4080<br>*Podaxis pistillaris*

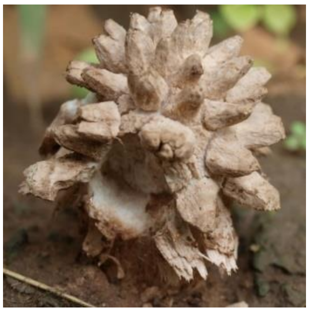<br>BAB 4081<br>*Phellorinia herculeana*	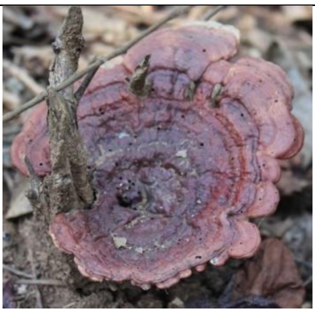<br>BAB 4082<br>*Ganoderma multipileum*	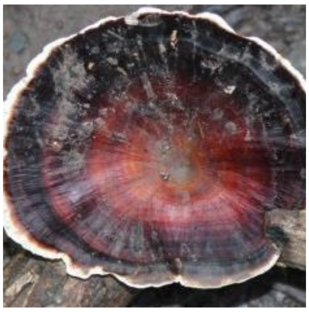<br>BAB 4083<br>*Microporus vernicipes*
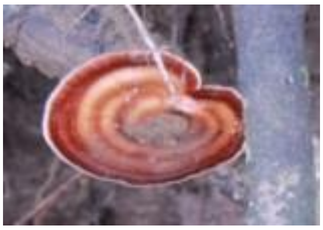<br>BAB 4084<br>*Microporus vernicipes*	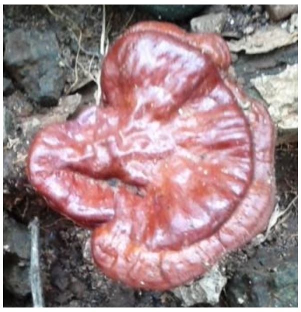<br>BAB 4085<br>*Ganoderma multipileum*	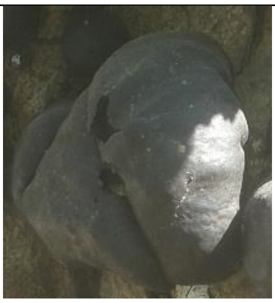<br>BAB 3279<br>*Daldinia eschscholzii*
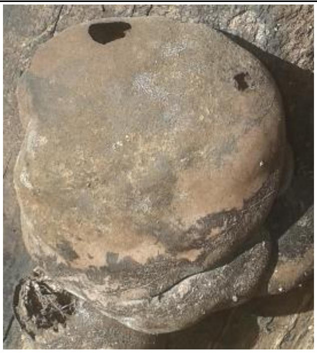<br>BAB 3296<br>*Daldinia eschscholzii*	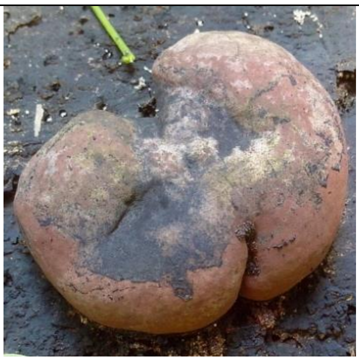<br>BAB 3298<br>*Daldinia eschscholzii*	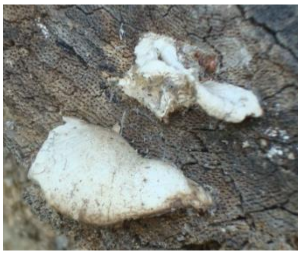<br>BAB 4366<br>*Dichomitus squalens*
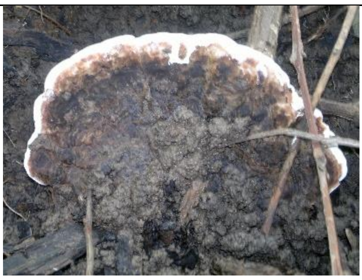<br>BAB 4560<br>*Ganoderma australe*	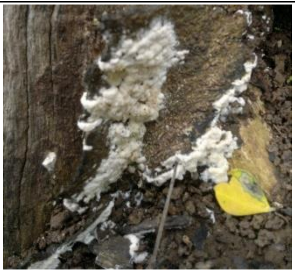<br>BAB 4561<br>*Oxyporus corticola*	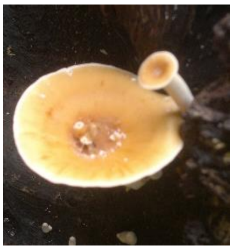<br>BAB 4716<br>*Polyporus leprieurii*

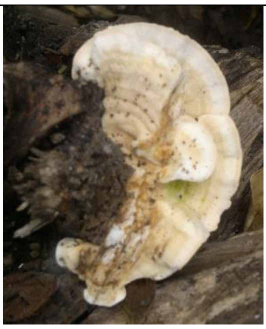<br>BAB 4718<br>*Lenzites betulinus*	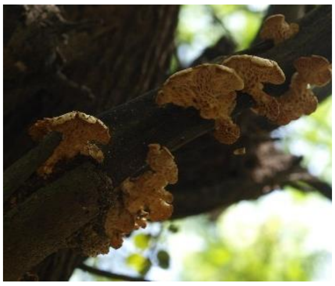<br>BAB 4719<br>*Polyporus tenuiculus*	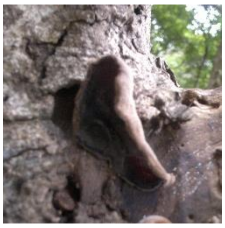<br>BAB 4720<br>*Auricularia polytricha*
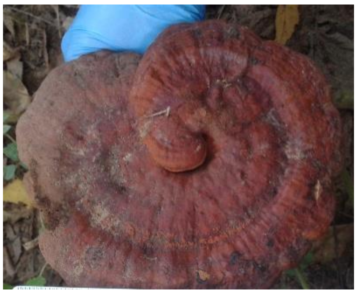<br>BAB 4721<br>*Ganoderma multipileum*	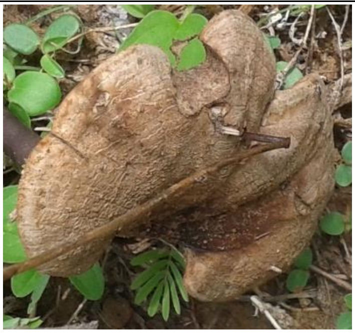<br>BAB 4722<br>*Ganoderma neojaponicum*	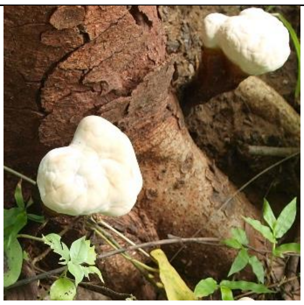<br>BAB 4723<br>*Ganoderma multipileum*
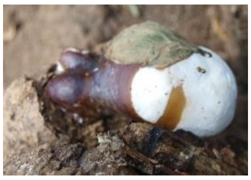<br>BAB 4724<br>*Ganoderma multipileum*	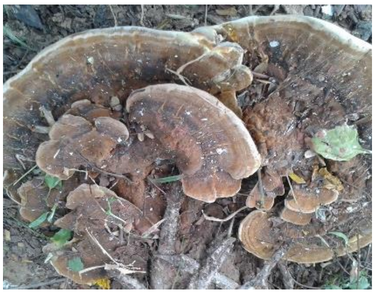<br>BAB 4725<br>*Ganoderma applanatum*	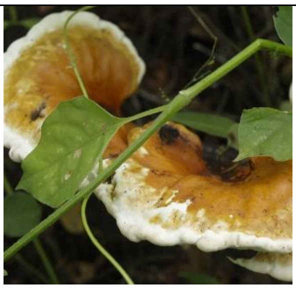<br>BAB 4726<br>*Ganoderma multipileum*
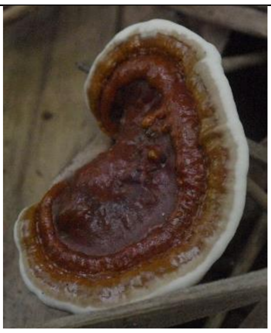<br>BAB 4727<br>*Ganoderma multipileum*	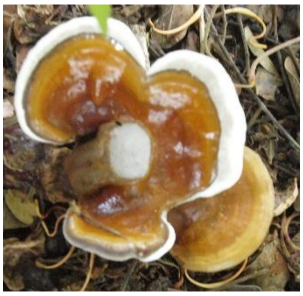<br>BAB 4728<br>*Ganoderma carnosum*	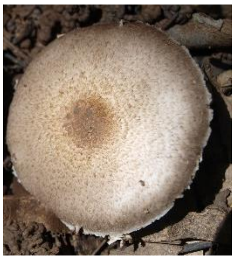<br>BAB 4729<br>*Agaricus augustus*

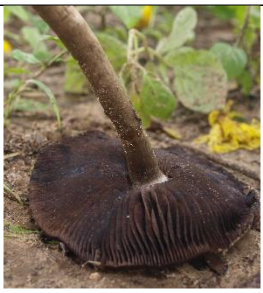

BAB 4730
Agaricus xanthodermus

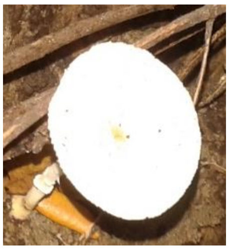

BAB 4731
Leucocoprinus brebissonii

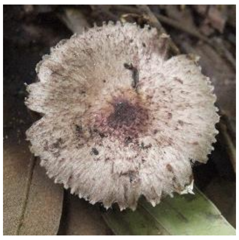

BAB 4732
Agaricus purpurellus

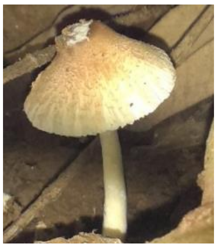

BAB 4733
Leucoagaricus littoralis

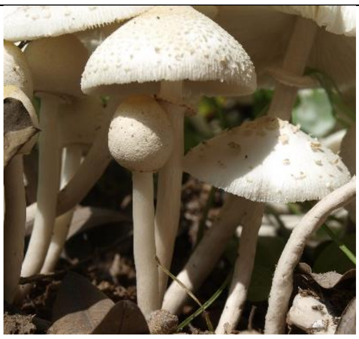

BAB 4734
Chlorophyllum hortense

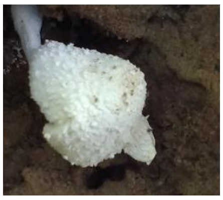

BAB 4735
Leucocoprinus cretaceus

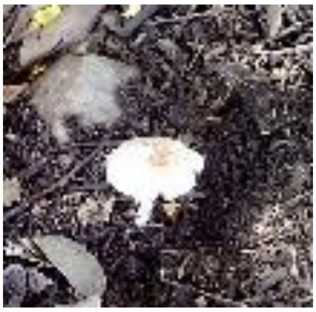

BAB 4736
Agaricus augustus

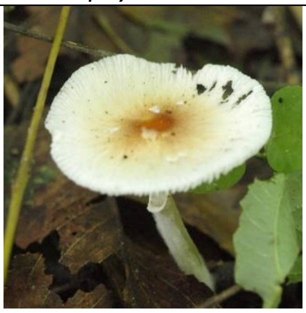

BAB 4737
Leucoagaricus rubrotinctus

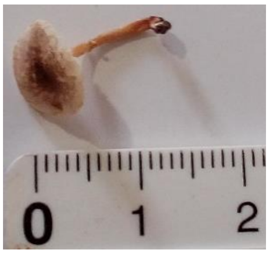

BAB 4738
Lepiota flammeotincta

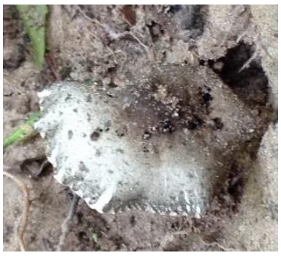

BAB 4739
Agaricus moelleri

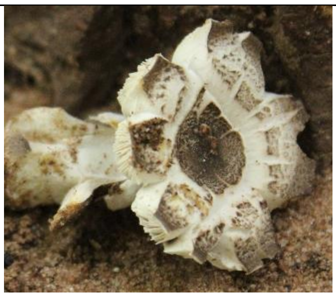

BAB 4740
Agaricus placomyces

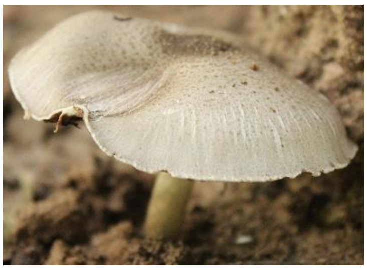

BAB 4741
Agaricus moelleri

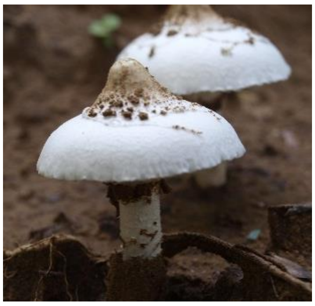<br>BAB 4742<br>*Termitomyces heimii*	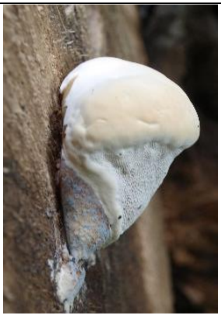<br>BAB 4743<br>*Dichomitus squalens*	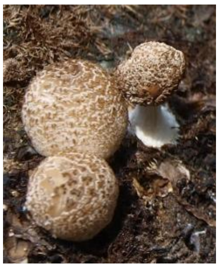<br>BAB 4744<br>*Coprinellus radians*
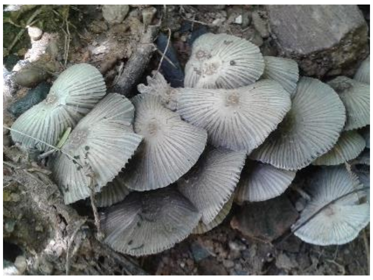<br>BAB 4745<br>*Coprinellus micaceus*	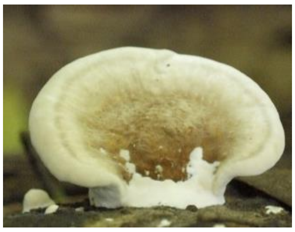<br>BAB 4746<br>*Lenzites elegans*	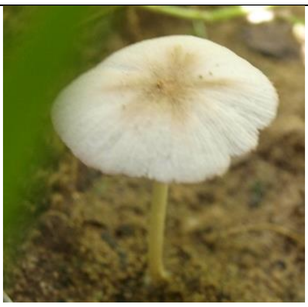<br>BAB 4747<br>*Psathyrella candolleana*
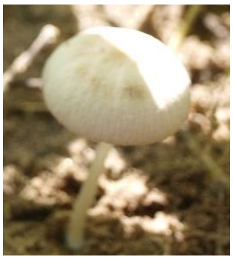<br>BAB 4748<br>*Psathyrella candolleana*	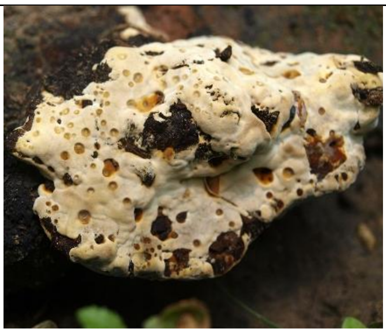<br>BAB 4749<br>*Inonotus porrectus*	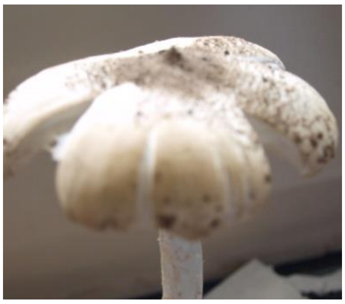<br>BAB 4750<br>*Termitomyces microcarpus*
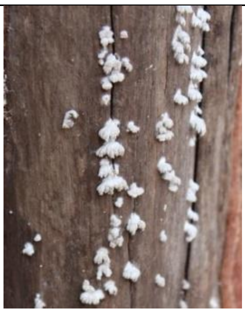<br>BAB 4751<br>*Schizophyllum commune*	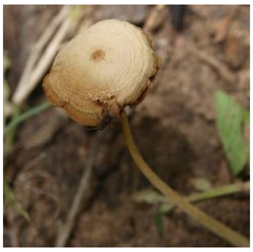<br>BAB 4752<br>*Conocybe papillata*	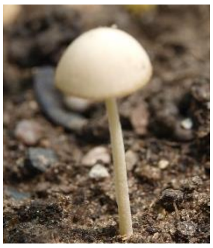<br>BAB 4753<br>*Conocybe lactea*

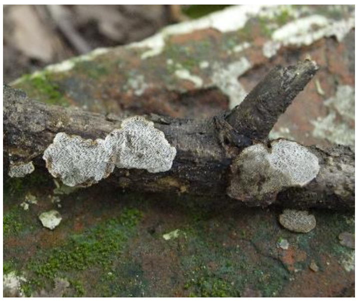

BAB 4754
Daedaleopsis confragosa

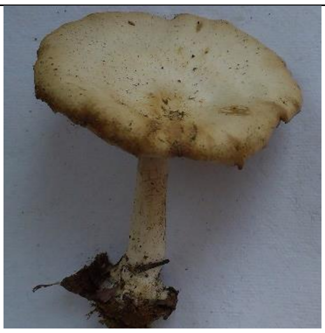

BAB 4755
Lyophyllum fumosum

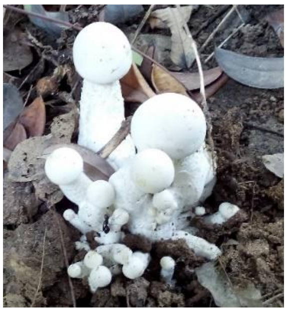

BAB 4756
Pleurotus nebrodensis

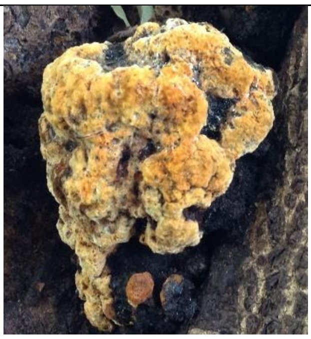

BAB 4757
Inonotus porrectus

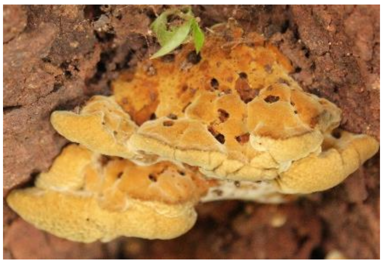

BAB 4758
Inonotus porrectus

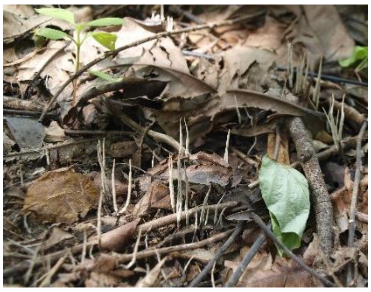

BAB 4759
Xylaria regalis

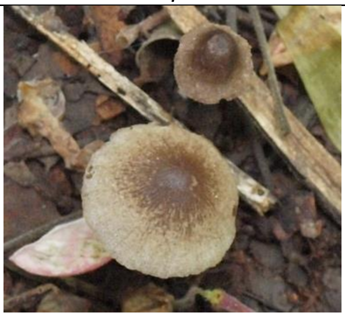

BAB 4760
Psathyrella candolleana

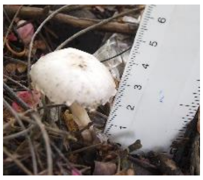

BAB 4761
Clitopilus prunulus

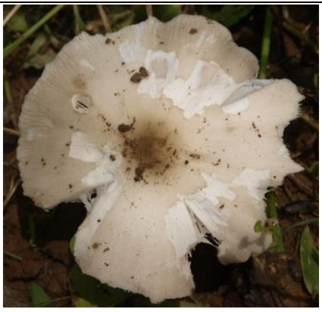

BAB 4762
Termitomyces eurrhizus

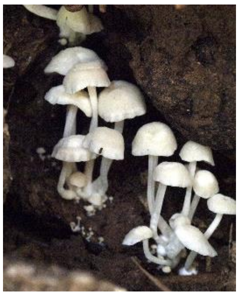

BAB 4763
Mycena corynephora

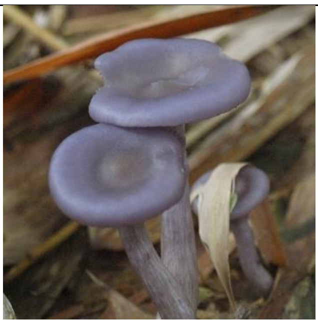

BAB 4764
Lepista sordida

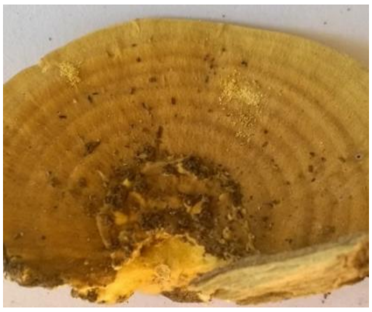

BAB 4765
Lenzites elegans

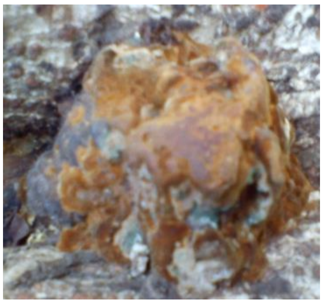

BAB 4818
Fulvifomes fastuosus

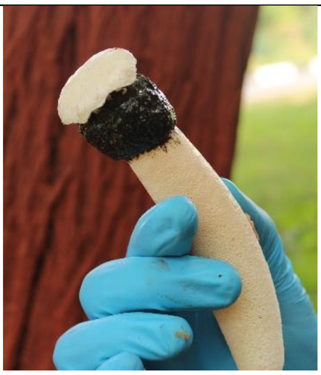

BAB 4821
Itajahya rosea

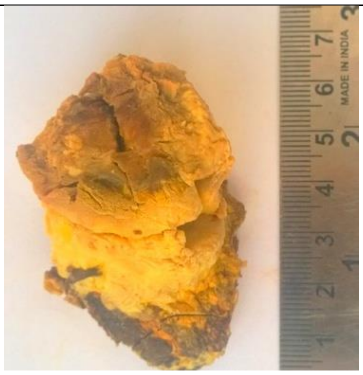

BAB 4822
Ganoderma colossus

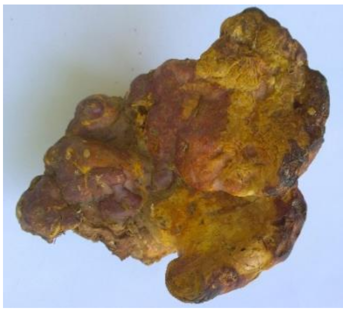

BAB 4981
Ganoderma lucidum

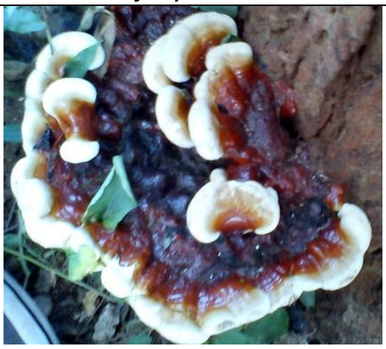

BAB 4922
Ganoderma carnosum

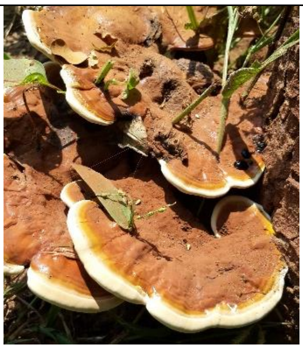

BAB 4929
Ganoderma applanatum

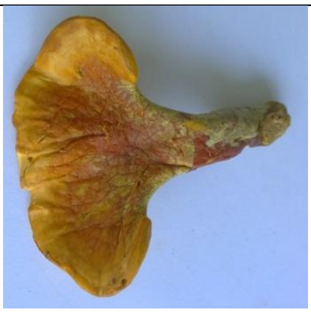

BAB 4923
Ganoderma lucidum

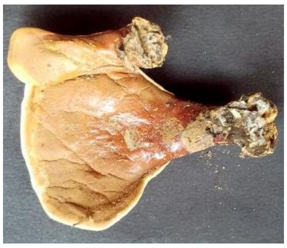

BAB 4986
Ganoderma multipileum

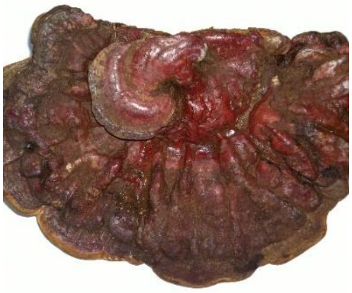

BAB 4987
Ganoderma multipileum

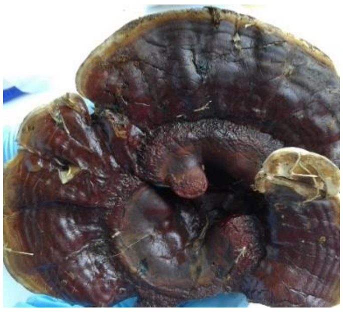

BAB 4988
Ganoderma tropicum

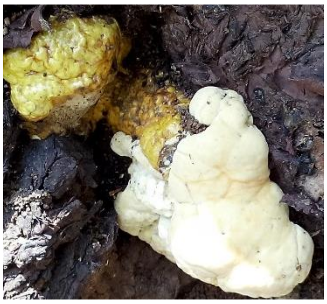

BAB 4989
Ganoderma colossus

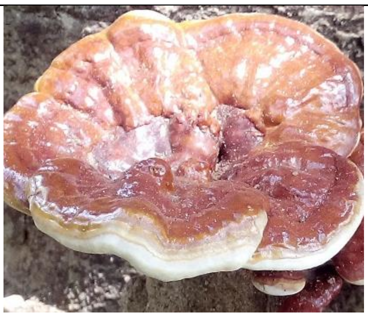

BAB 4926
Ganoderma multipileum

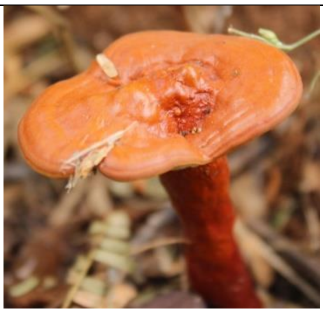<br>BAB 4924<br>*Ganoderma multipileum*	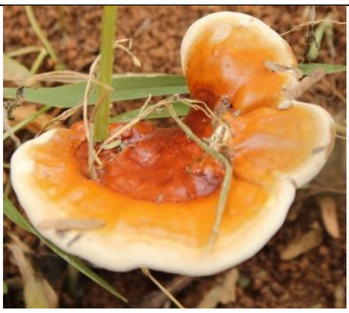<br>BAB 4925<br>*Ganoderma lucidum*	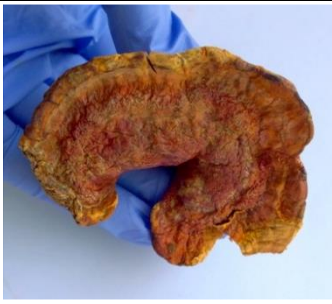<br>BAB 4927<br>*Ganoderma multipileum*
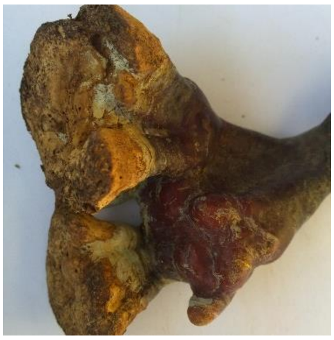<br>BAB 4984<br>*Ganoderma lucidum*	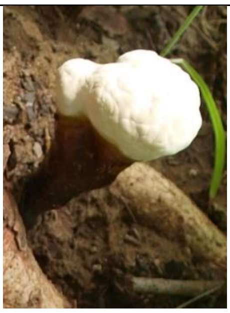<br>BAB 4928<br>*Ganoderma lucidum*	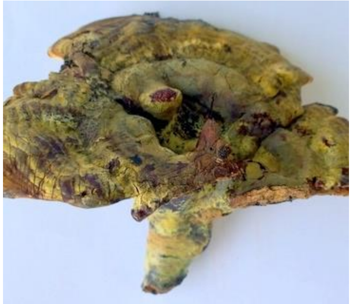<br>BAB 4990<br>*Ganoderma multipileum*
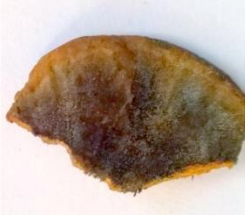<br>BAB 4982<br>*Ganoderma lucidum*	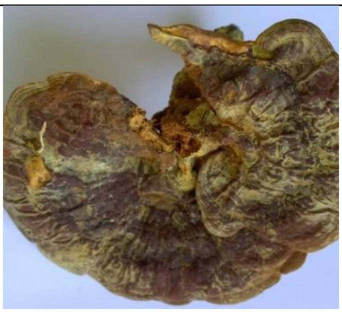<br>BAB 4983<br>*Ganoderma multipileum*	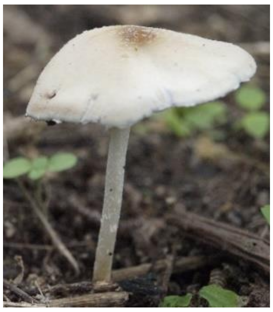<br>BAB 4913<br>*Psathyrella candolleana*
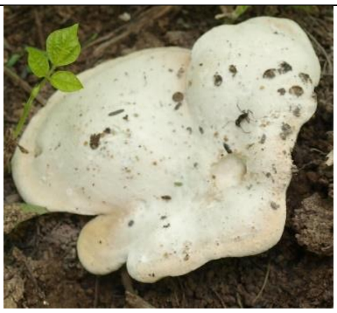<br>BAB 4914<br>*Clitopilus prunulus*	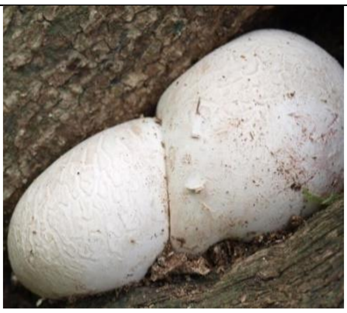<br>BAB 4917<br>*Itajahya rosea*	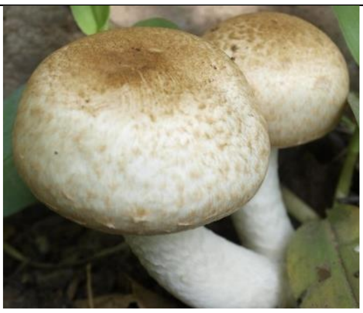<br>BAB 4970<br>*Agaricus blazei*

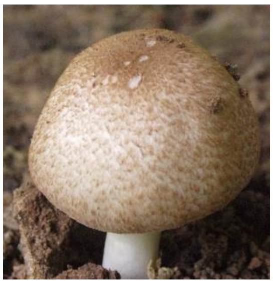<br>BAB 4971<br>*Agaricus hondensis*	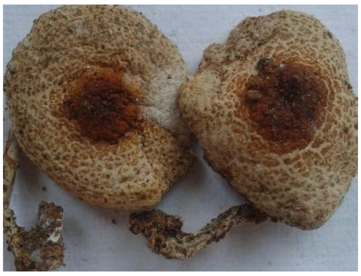<br>BAB 4972<br>*Lepiota subclypeolaria*	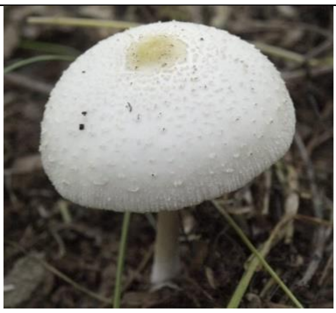<br>BAB 4973<br>*Leucoagaricus hortensis*
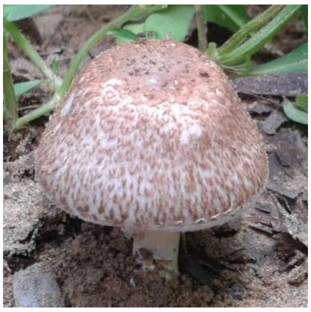<br>BAB 4974<br>*Agaricus langei*	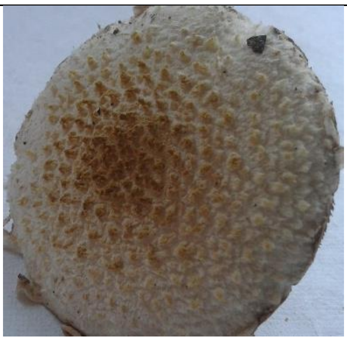<br>BAB 4975<br>*Agaricus augustus*	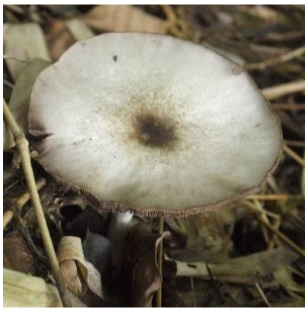<br>BAB 4976<br>*Agaricus pocillator*
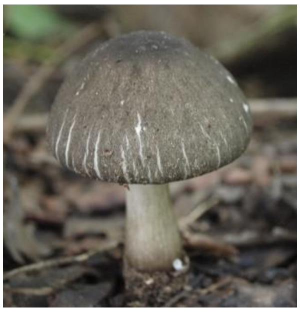<br>BAB 4978<br>*Agaricus rotalis*	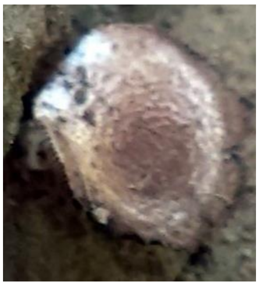<br>BAB 4979<br>*Lepiota subclypeolaria*	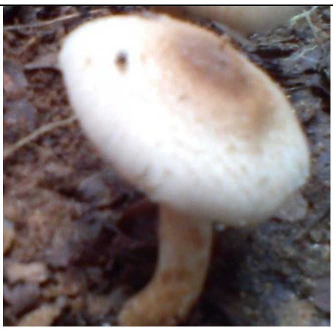<br>BAB 4980<br>*Lepiota subclypeolaria*
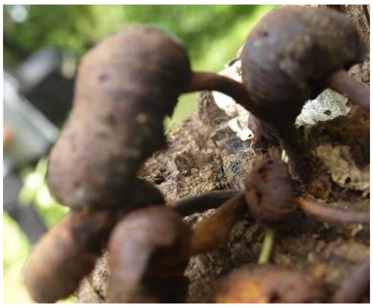<br>BAB 4937<br>*Entoloma flocculosum*	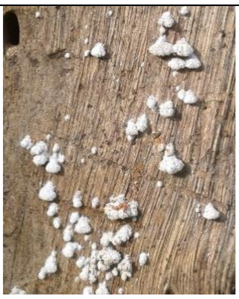<br>BAB 4938<br>*Schizophyllum commune*	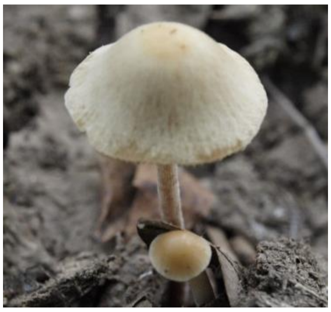<br>BAB 4939<br>*Psathyrella candolleana*

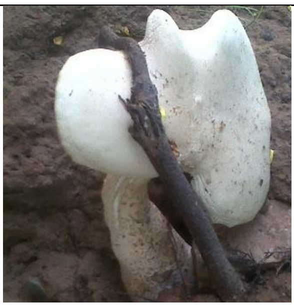<br>BAB 4940<br>*Tricholoma giganteum*	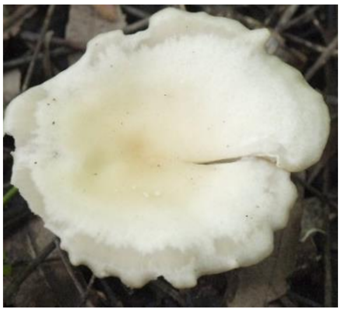<br>BAB 4941<br>*Clitocybe metachroa*	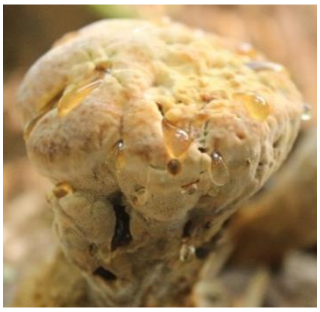<br>BAB 4942<br>*Inonotus porrectus*
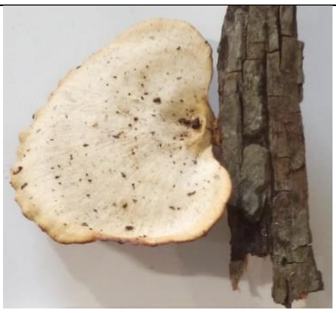<br>BAB 4943<br>*Polyporus grammocephalus*	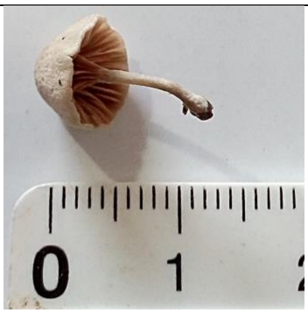<br>BAB 4944<br>*Collybia hariolorum*	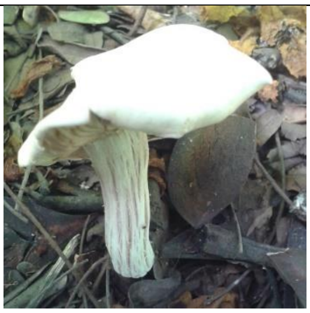<br>BAB 4945<br>*Clitopilus prunulus*
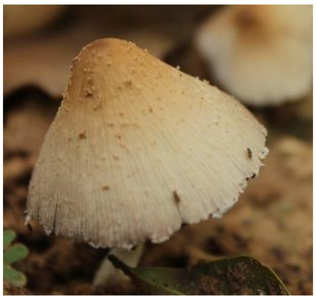<br>BAB 4946<br>*Psathyrella candolleana*	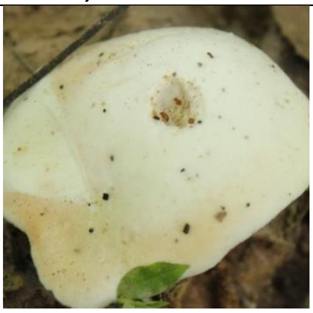<br>BAB 4947<br>*Tricholoma mongolicum*	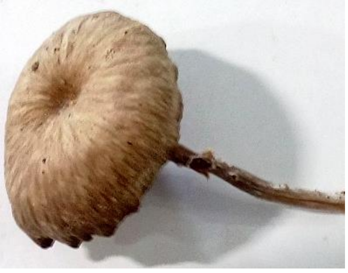<br>BAB 4948<br>*Omphalina rivulicola*
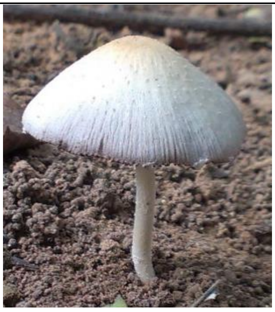<br>BAB 4949<br>*Psathyrella candolleana*	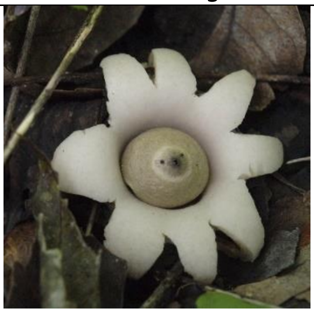<br>BAB 4951<br>*Geastrum striatum*	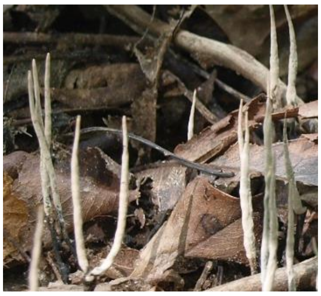<br>BAB 4952<br>*Xylaria psidii*

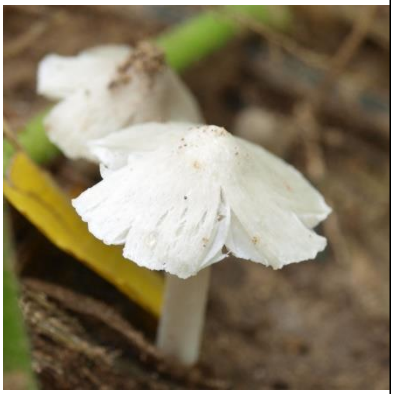<br>BAB 4953<br>*Termitomyces eurrhizus*	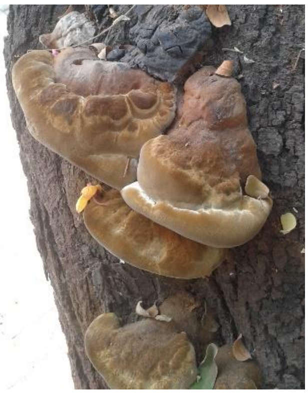<br>BAB 4954<br>*Phellinus merrillii*	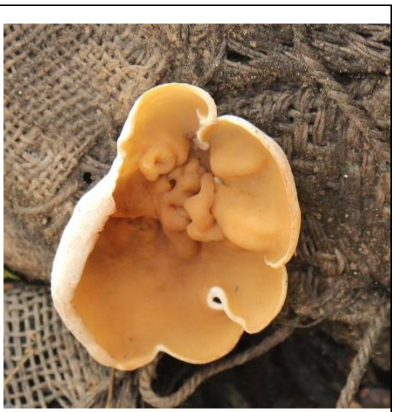<br>BAB 4955<br>*Peziza arvernensis*
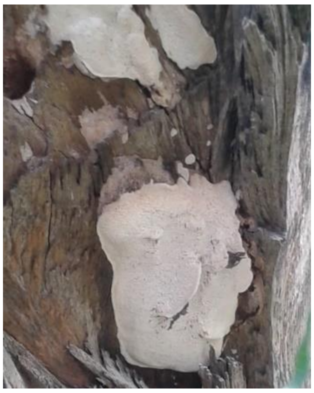<br>BAB 4956<br>*Perenniporia tephropora*	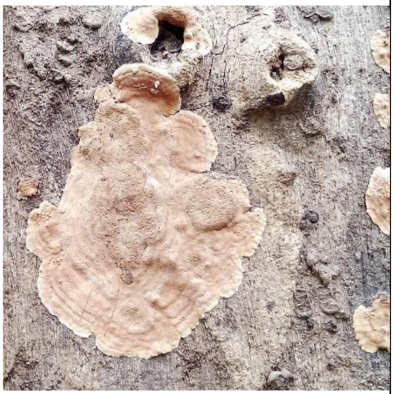<br>BAB 4957<br>*Hypoxylon rickii*	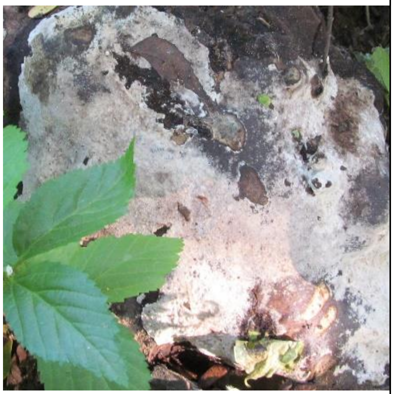<br>BAB 4958<br>*Perenniporia tephropora*
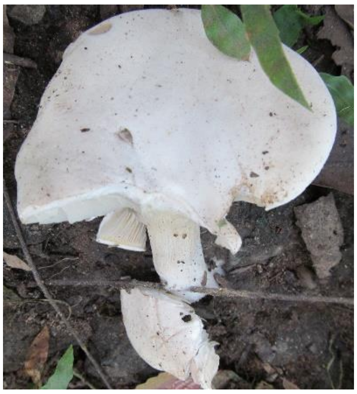<br>BAB 4959<br>*Clitopilus prunulus*	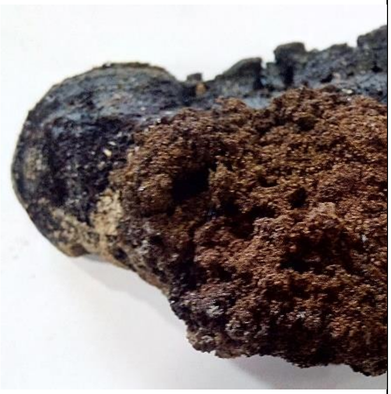<br>BAB 4960<br>*Phellinus bicuspidatus*	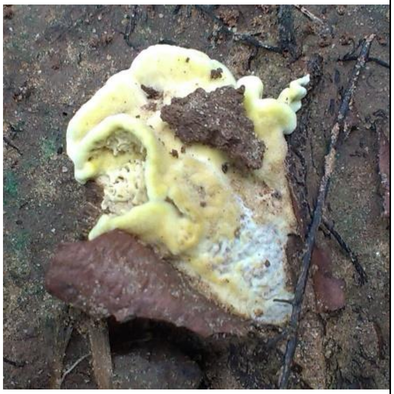<br>BAB 4961<br>*Flavodon flavus*

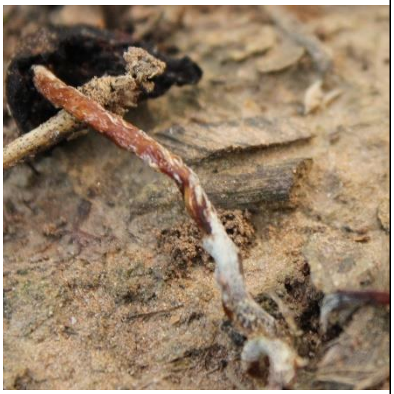
BAB 4963
Coprinus silvaticus

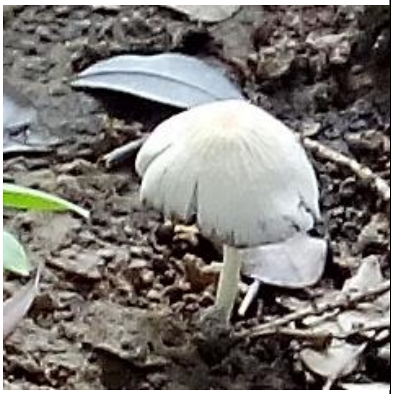
BAB 4964
Psathyrella candolleana

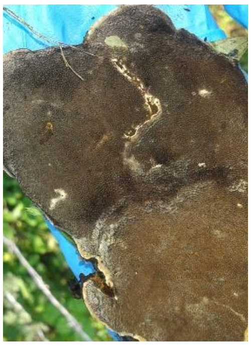
BAB 4965
Phellinus robiniae

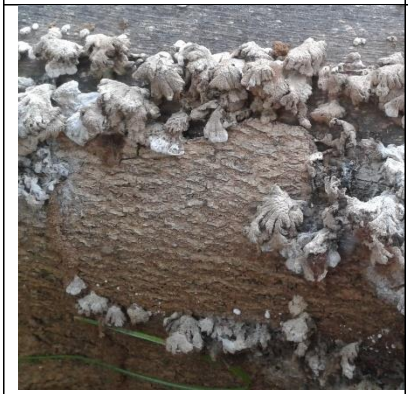
BAB 4966
Schizophyllum commune

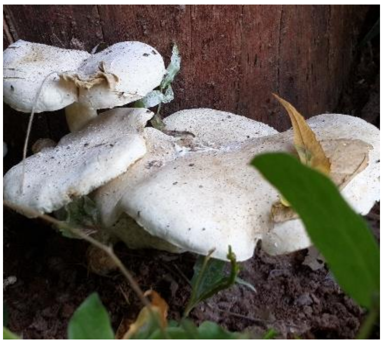
BAB 4967
Clitopilus prunulus

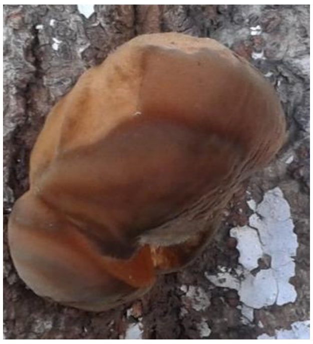
BAB 4968
Phellinus merrillii

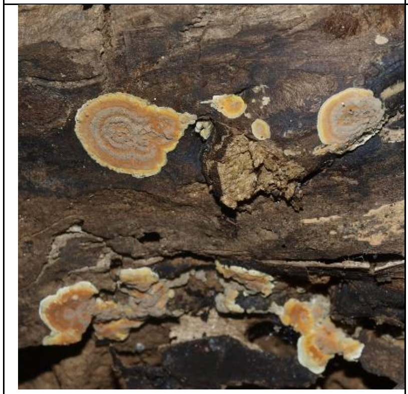
BAB 4969
Hypoxylon rickii

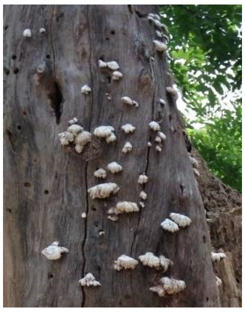
BAB 5051
Schizophyllum commune

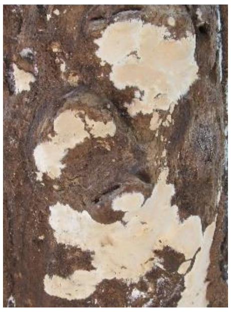
BAB 5052
Ceriporia lacerata

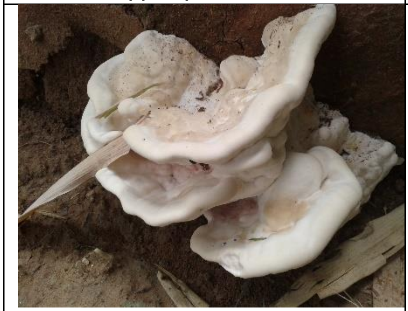
BAB 5055
Amylosporus campbellii

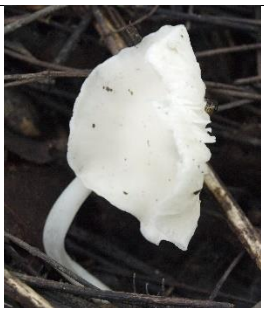
BAB 5056
Clitopilus scyphoides

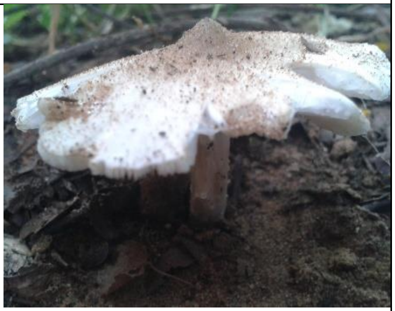
BAB 5063
Termitomyces eurrhizus

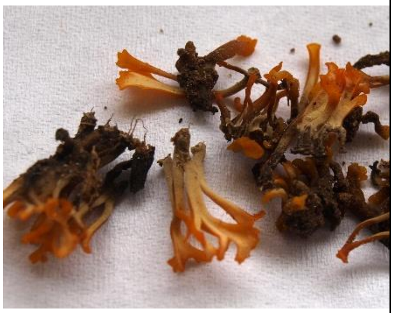<br>BAB 5064<br>*Dacryopinax spathularia*	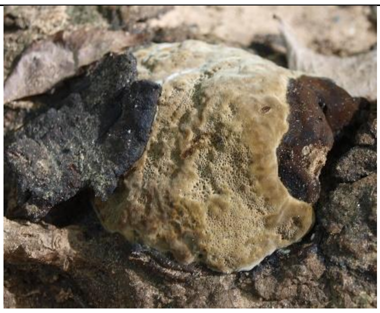<br>BAB 5065<br>*Fulvifomes fastuosus*	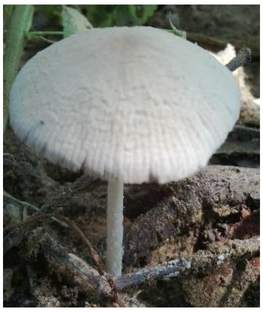<br>BAB 5067<br>*Psathyrella candolleana*
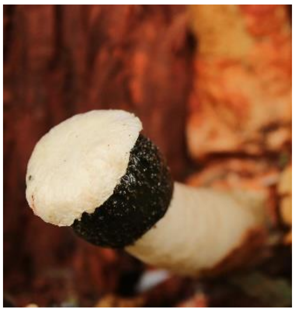<br>BAB 5069<br>*Itajahya rosea*	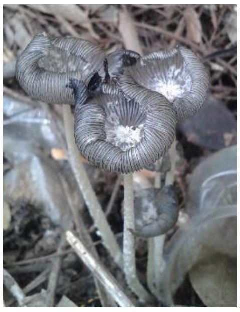<br>BAB 5070<br>*Coprinopsis cinerea*	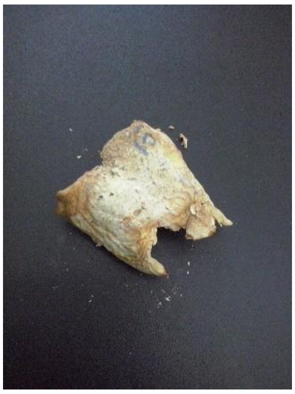<br>BAB 4962<br>*Clitopilus prunulus*
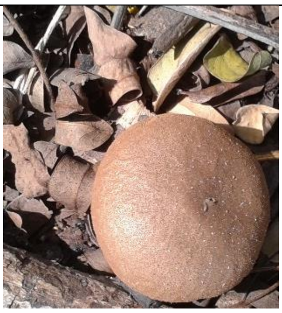<br>BAB 5048<br>*Lycoperdon pusillum*	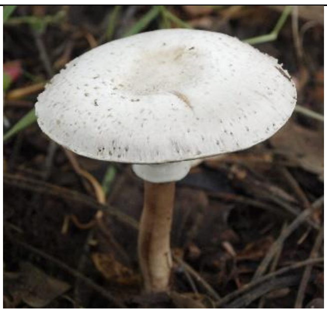<br>BAB 5050<br>*Leucoagaricus leucothites*	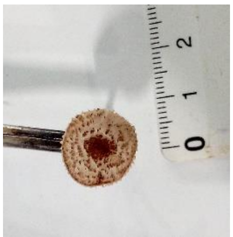<br>BAB 5053<br>*Lepiota flammeotincta*
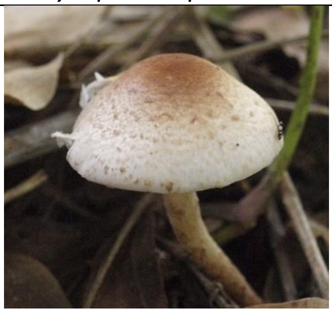<br>BAB 5057<br>*Agaricus diminutivus*	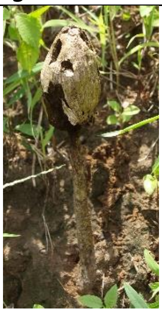<br>BAB 5058<br>*Podaxis pistillaris*	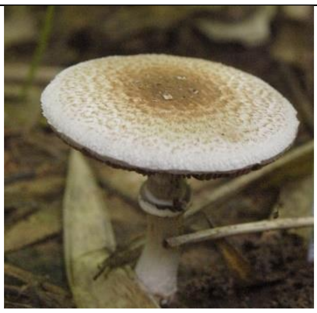<br>BAB 5059<br>*Agaricus romagnesii*

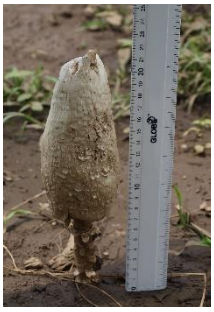
BAB 5071
Podaxis pistillaris

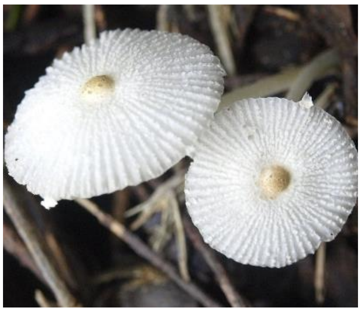
BAB 5104
Leucocoprinus fragilissimus

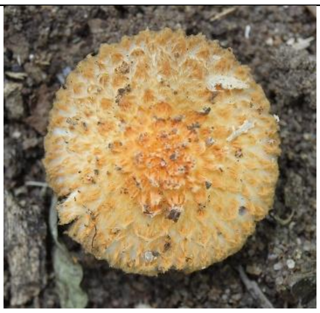
BAB 5054
Agaricus trisulphuratus

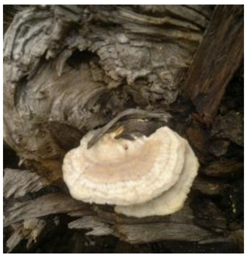
BAB 5072
Fomitopsis palustris

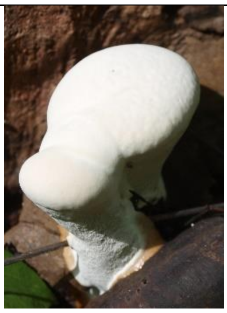
BAB 5105
Ganoderma lucidum

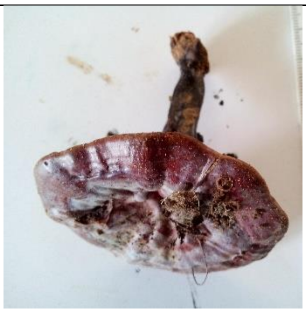
BAB 5106
Ganoderma multipileum

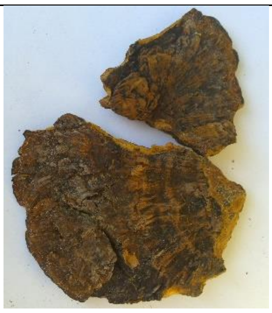
BAB 5118
Phellinus robiniae

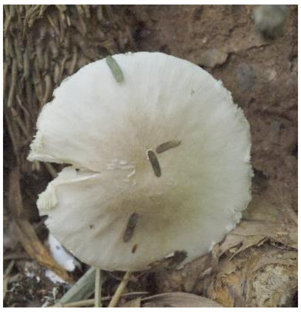
BAB 5119
Agaricus californicus

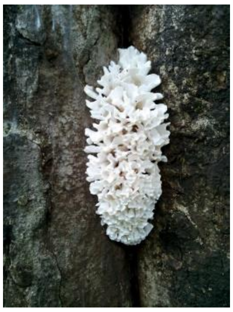
BAB 5120
Scytinopogon sp.

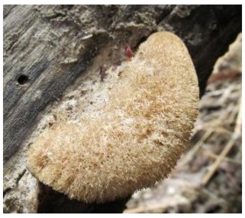
BAB 5121
Trametes trogii

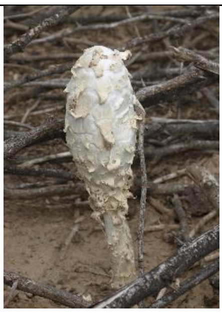
BAB 5122
Podaxis pistillaris

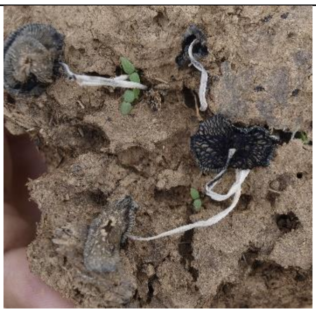
BAB 5123
Coprinopsis cinerea
