# Supplementary material for: Molecular survey of basidiomycetes and divergence time estimation: An Indian perspective
Source: PLoS One. 2018 May 17;13(5):e0197306. doi: 10.1371/journal.pone.0197306 (PMC5957343; doi:10.1371/journal.pone.0197306)

S2 Fig. Phylogenetic trees generated using Maximum likelihood and Maximum parsimony analysis for different families in the study

Figure S2 (a) – ITS phylogeny of the Agaricaceae family inferred by Maximum Likelihood analysis. The evolutionary history was inferred by using the Maximum Likelihood method based on the Tamura-Nei model. The bootstrap consensus tree inferred from 500 replicates is taken to represent the evolutionary history of the taxa analyzed. Branches corresponding to partitions reproduced in less than 50% bootstrap replicates are collapsed.

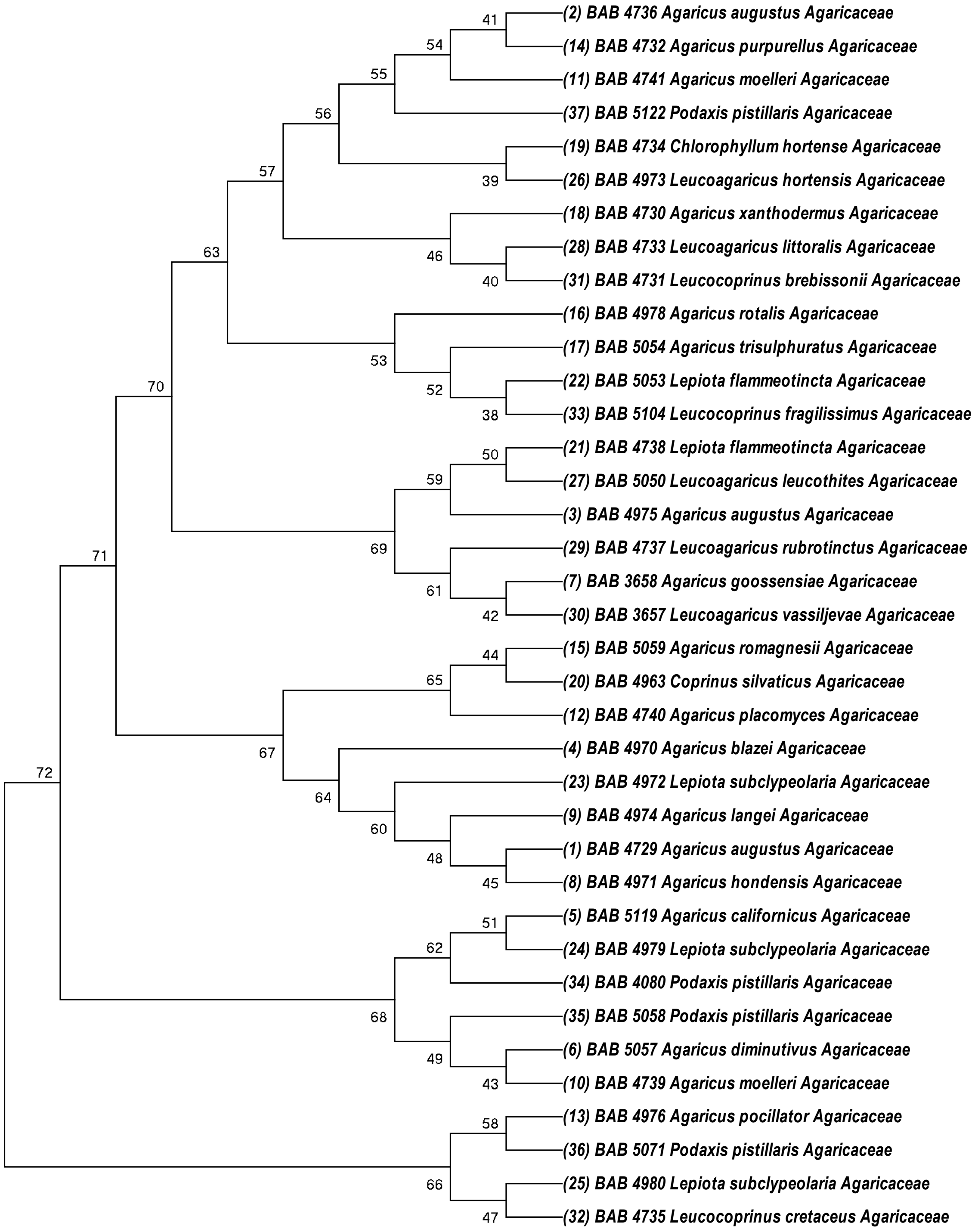

Figure S2 (b) – ITS phylogeny of the Agaricaceae family inferred by Maximum Parsimony analysis. The most parsimonious tree with length = 5916 is shown. The consistency index is (0.189148), the retention index is (0.350528), and the composite index is 0.066302 (0.066302) for all sites and parsimony-informative sites. The MP tree was obtained using the Subtree-Pruning-Regrafting (SPR) algorithm with search level 1 in which the initial trees were obtained by the random addition of sequences.

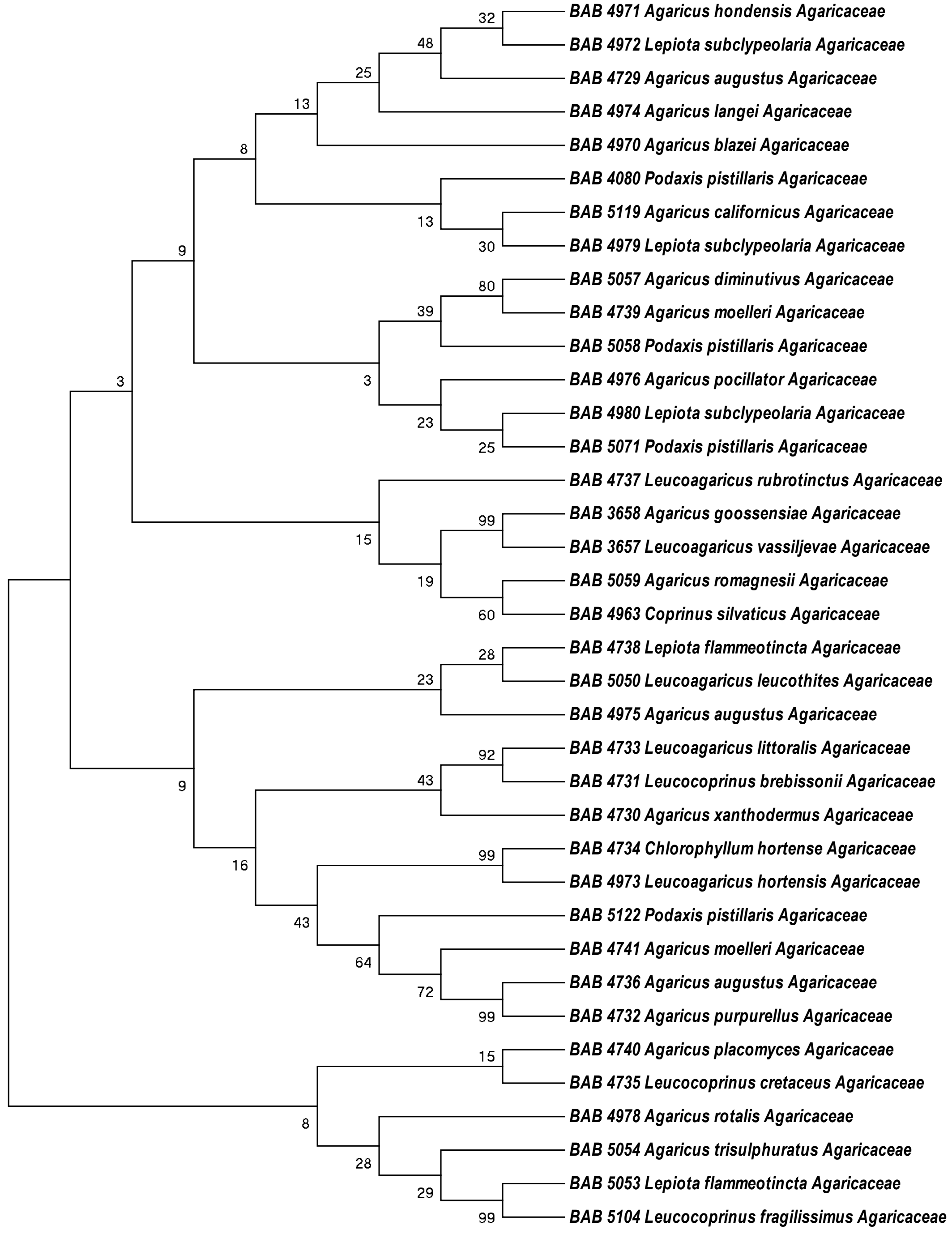

Figure S2 (C) - ITS phylogeny of the Polyporaceae family inferred by Maximum Likelihood analysis based on the Tamura-Nei model. The tree with the highest log likelihood (-14670.1530) is shown. Initial tree(s) for the heuristic search were obtained automatically by applying Neighbor-Join and BioNJ algorithms to a matrix of pairwise distances estimated using the Maximum Composite Likelihood (MCL) approach, and then selecting the topology with superior log likelihood value.

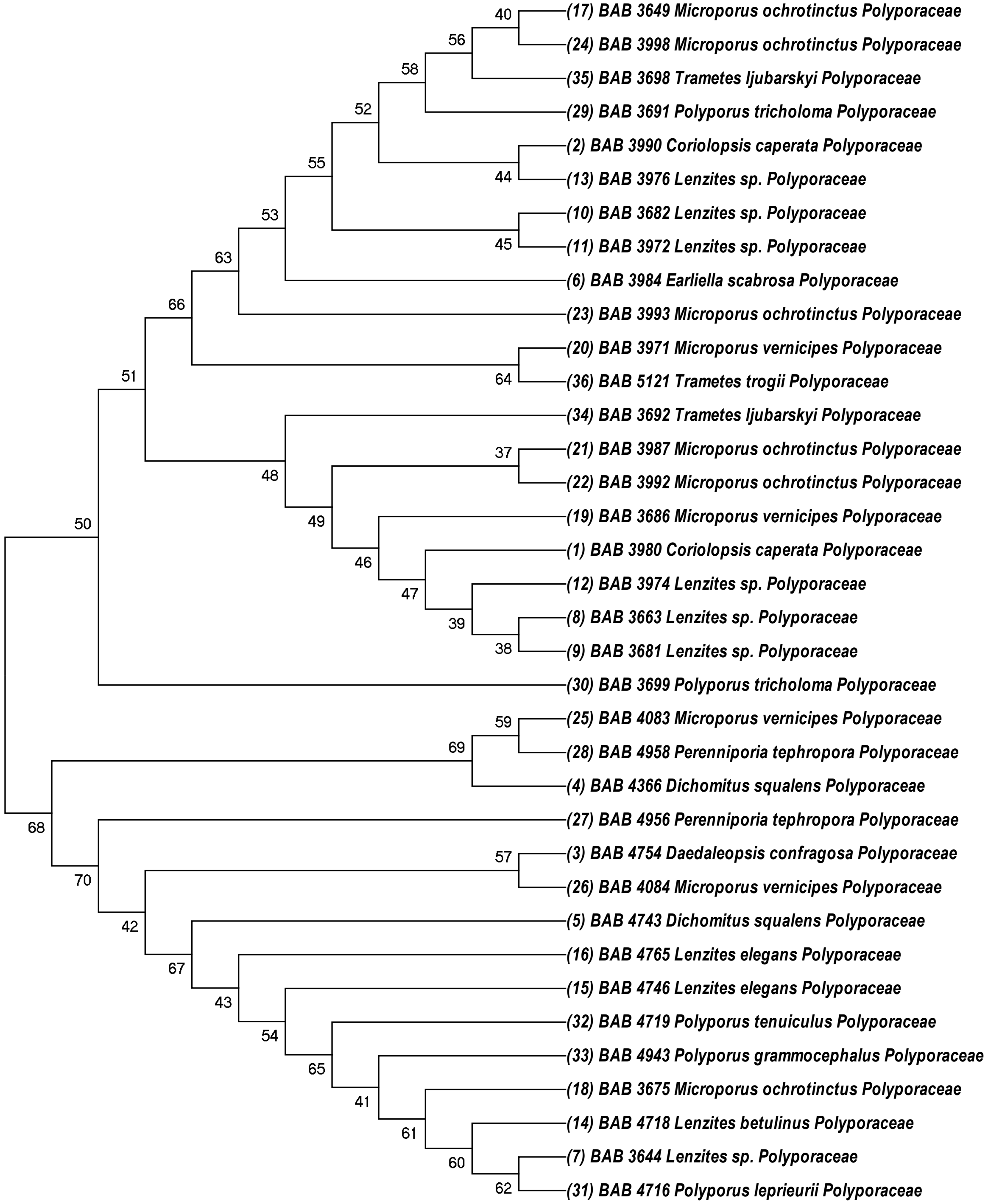

Figure S2 (D) - ITS phylogeny of the Polyporaceae family inferred by Maximum Parsimony method. The most parsimonious tree with length = 4887 is shown. The consistency index is (0.216493), the retention index is (0.422212), and the composite index is 0.091406 (0.091406) for all sites and parsimony-informative sites (in parentheses). The MP tree was obtained using the Subtree-Pruning-Regrafting (SPR) algorithm with search level 1 in which the initial trees were obtained by the random addition of sequences (10 replicates).

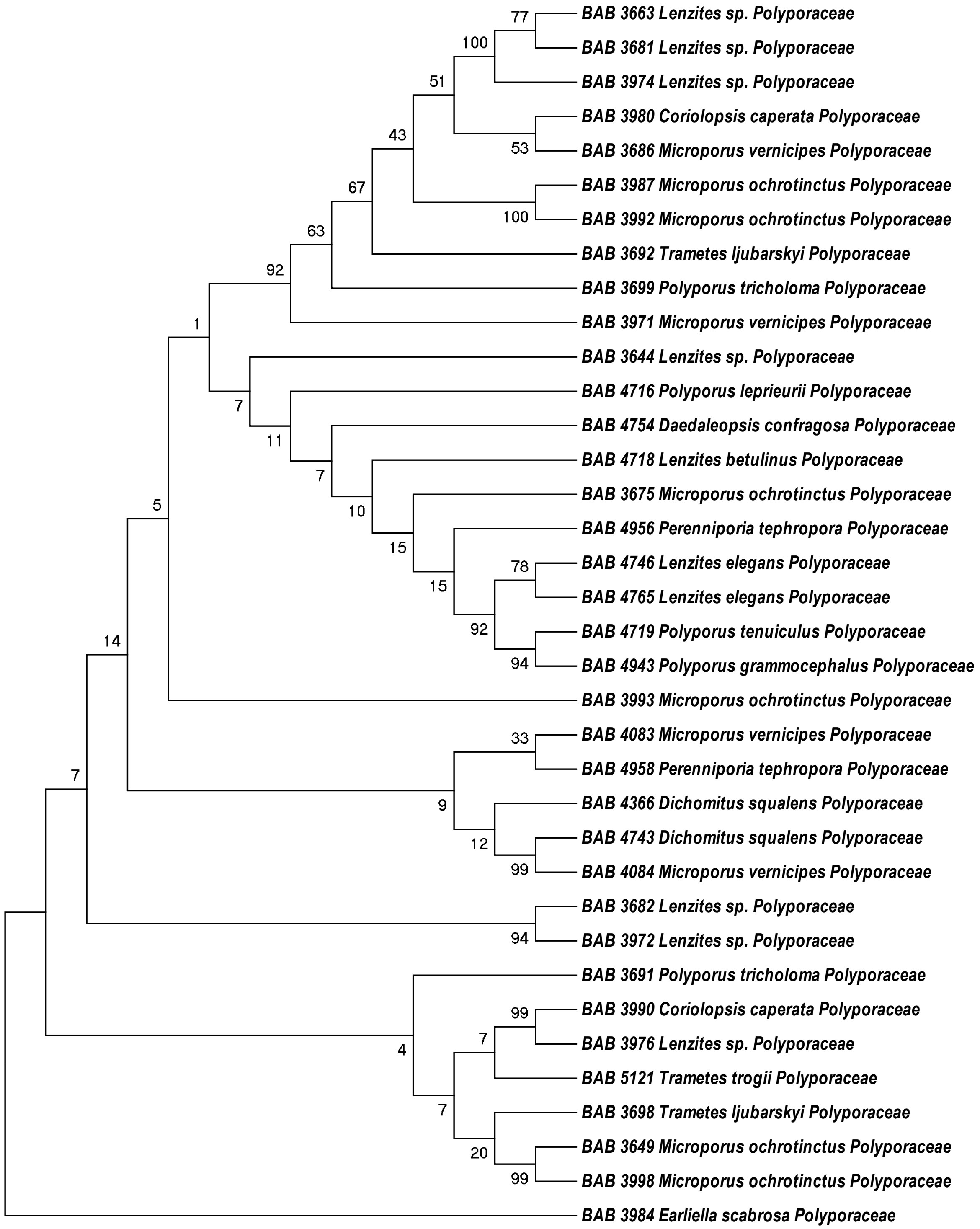

Figure S2 (E) - ITS phylogeny of the Tricholomatoid clade inferred by Maximum Likelihood analysis based on the Tamura-Nei model The tree with the highest log likelihood (-3513.0981) is shown. The percentage of trees in which the associated taxa clustered together is shown next to the branches.

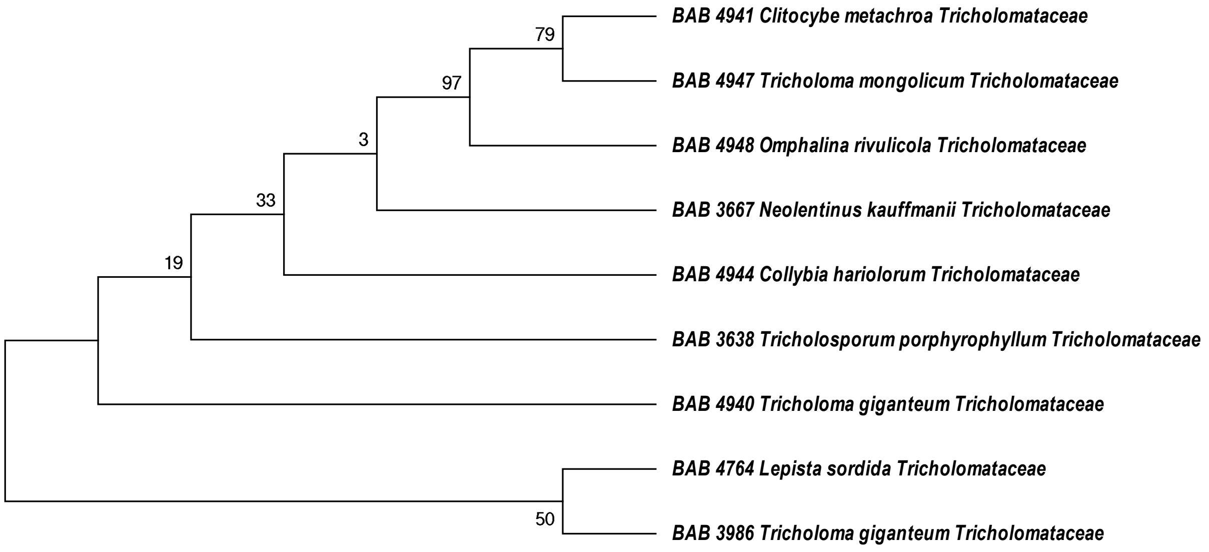

Figure S2 (F) - ITS phylogeny of the Tricholomatoid clade inferred by Maximum Parsimony analysis. The bootstrap consensus tree inferred from 500 replicates is taken to represent the evolutionary history of the taxa analyzed. Branches corresponding to partitions reproduced in less than 50% bootstrap replicates are collapsed. The MP tree was obtained using the Subtree-Pruning-Regrafting (SPR) algorithm with search level 1 in which the initial trees were obtained by the random addition of sequences (10 replicates).

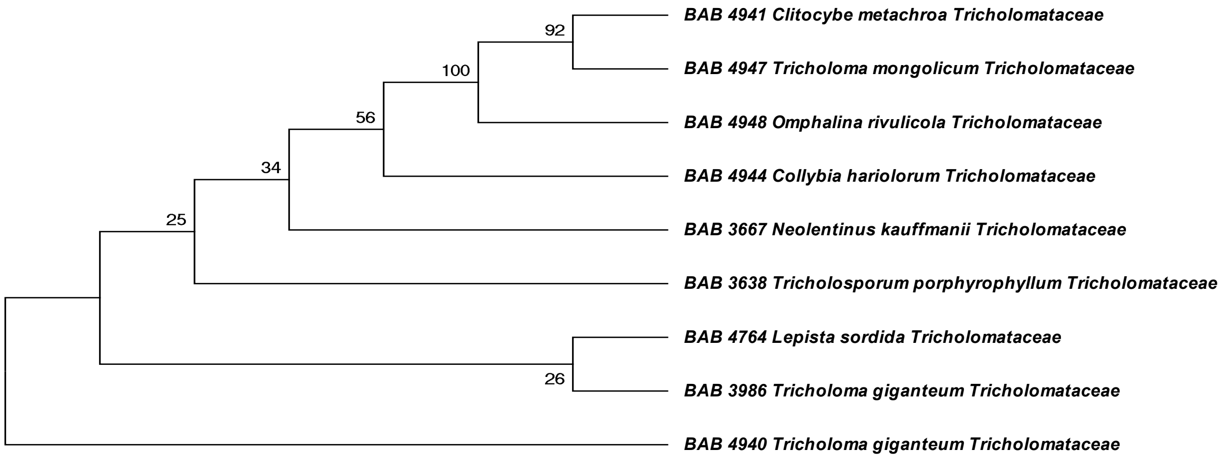

Figure S2 (E) - ITS phylogeny of the Xylaraceae clade inferred by Maximum Likelihood analysis based on the Tamura-Nei model The tree with the highest log likelihood (-3866.1108) is shown. The percentage of trees in which the associated taxa clustered together is shown next to the branches.

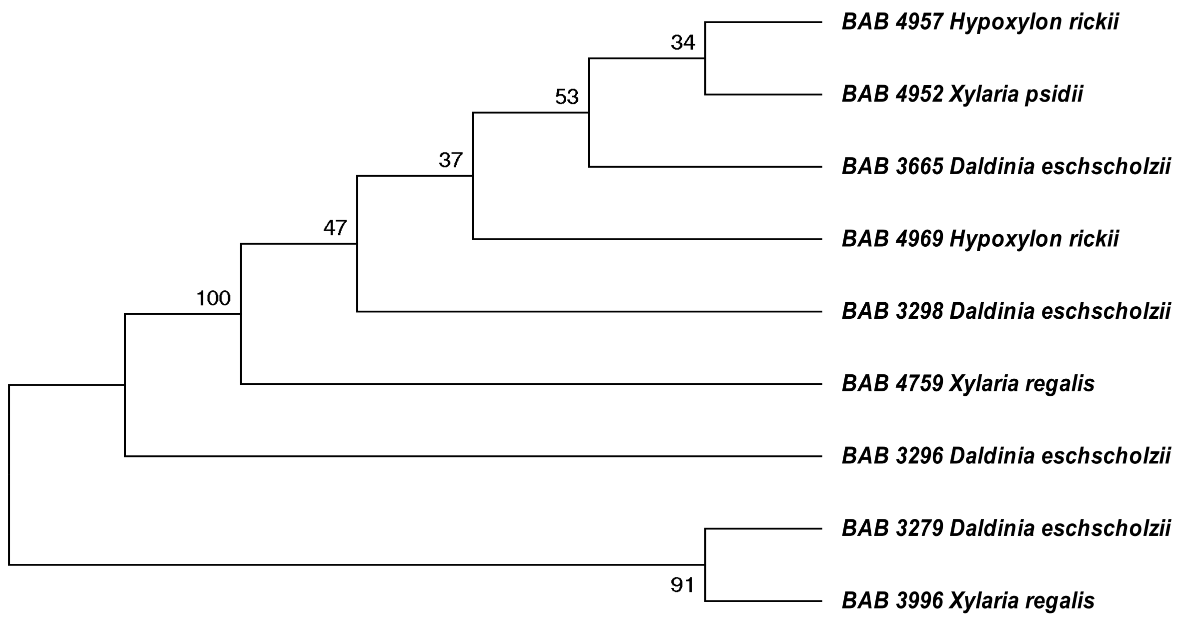

Figure S2 (F) - ITS phylogeny of the Xylaraceae clade inferred by Maximum Parsimony method. The most parsimonious tree with length = 1314 is shown. The consistency index is (0.641803), the retention index is (0.356145), and the composite index is 0.231196 (0.228575) for all sites and parsimony-informative sites. The MP tree was obtained using the Subtree-Pruning-Regrafting (SPR) algorithm with search level 1 in which the initial trees were obtained by the random addition of sequences (10 replicates).

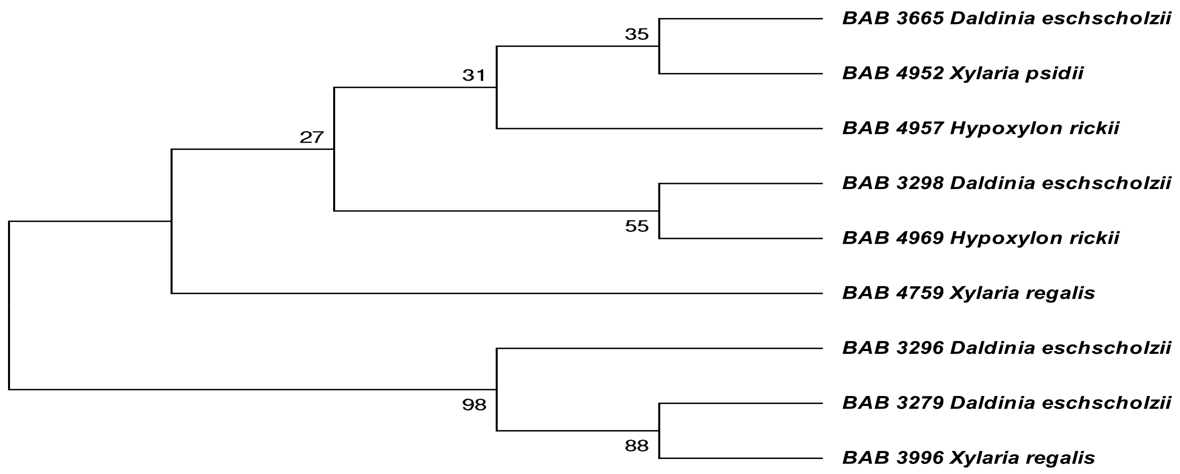

Figure S2 (G) – Phylogenetic trees for Psathyrellaceae generated by Maximum Likelihood method based on the Tamura-Nei model. Branches corresponding to partitions reproduced in less than 50% bootstrap replicates are collapsed. The percentage of replicate trees in which the associated taxa clustered together in the bootstrap test are shown next to the branches.

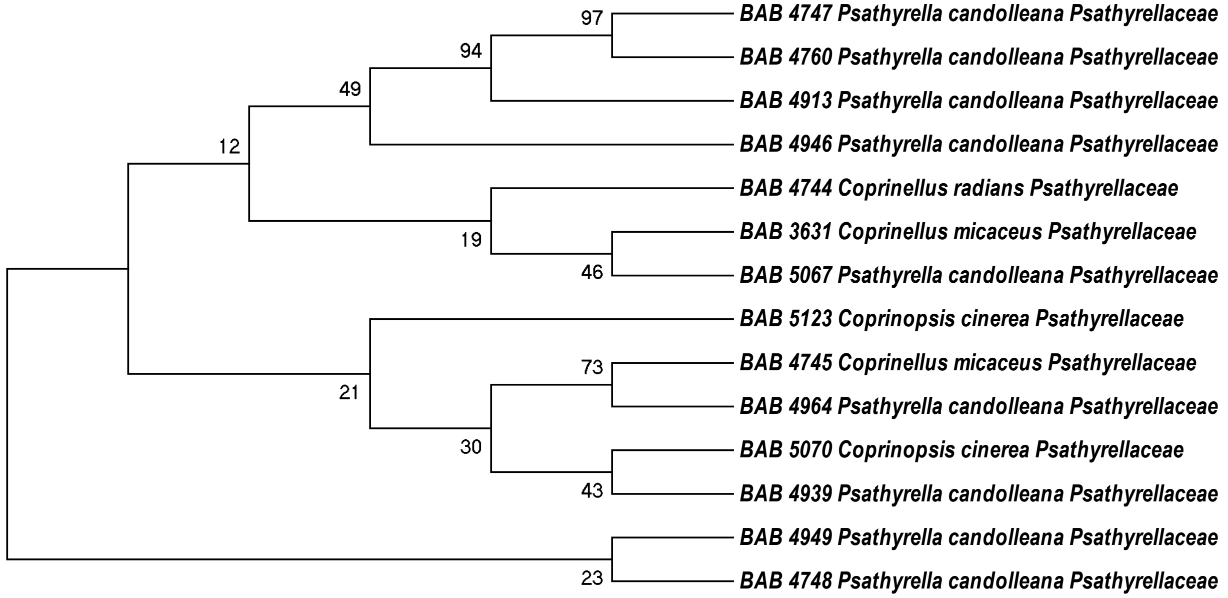

Figure S2 (H) - Phylogenetic trees for Psathyrellaceae generated by Maximum Parsimony method. The bootstrap consensus tree inferred from 500 replicates is taken to represent the evolutionary history of the taxa analyzed. Branches corresponding to partitions reproduced in less than 50% bootstrap replicates are collapsed. The MP tree was obtained using the Subtree-Pruning-Regrafting (SPR) algorithm

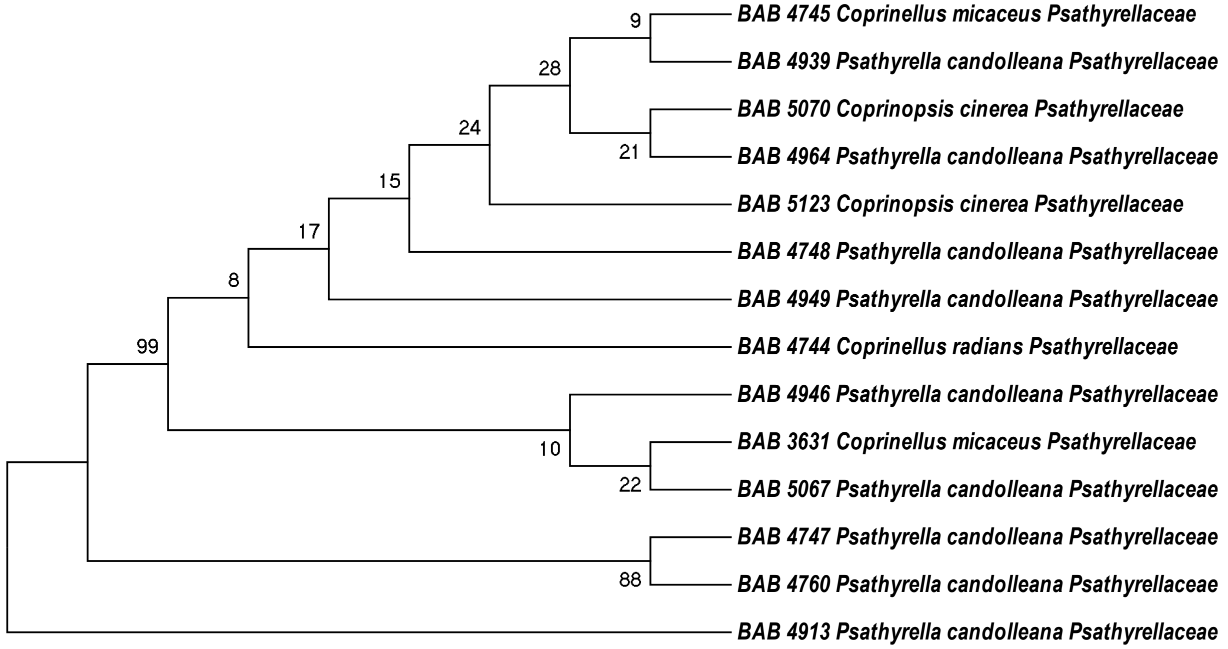

Figure S2 (I) – Phylogenetic trees generated by Maximum Likelihood method based on the Tamura-Nei model for Ganodermataceae. The tree with the highest log likelihood (-15102.0510) is shown. Initial tree(s) for the heuristic search were obtained automatically by applying Neighbor-Join and BioNJ algorithms to a matrix of pairwise distances estimated using the Maximum Composite Likelihood (MCL) approach, and then selecting the topology with superior log likelihood value.

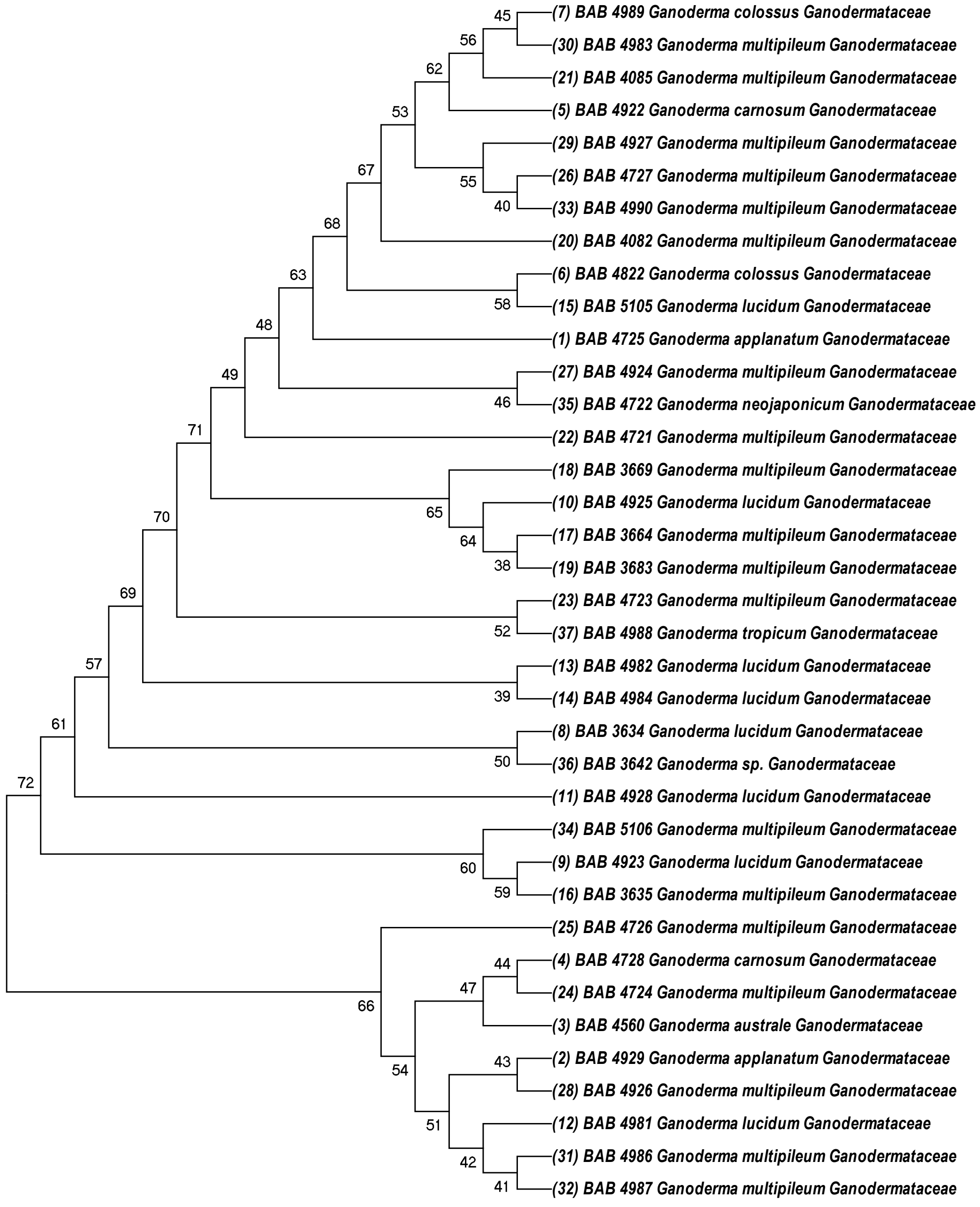

Figure S2 (J) – Phylogenetic trees generated by Maximum Parsimony method for Ganodermataceae. The bootstrap consensus tree inferred from 500 replicates is taken to represent the evolutionary history of the taxa analyzed. Branches corresponding to partitions reproduced in less than 50% bootstrap replicates are collapsed. The percentage of replicate trees in which the associated taxa clustered together in the bootstrap test (500 replicates) are shown next to the branches. The MP tree was obtained using the Subtree-Pruning-Regrafting (SPR) algorithm

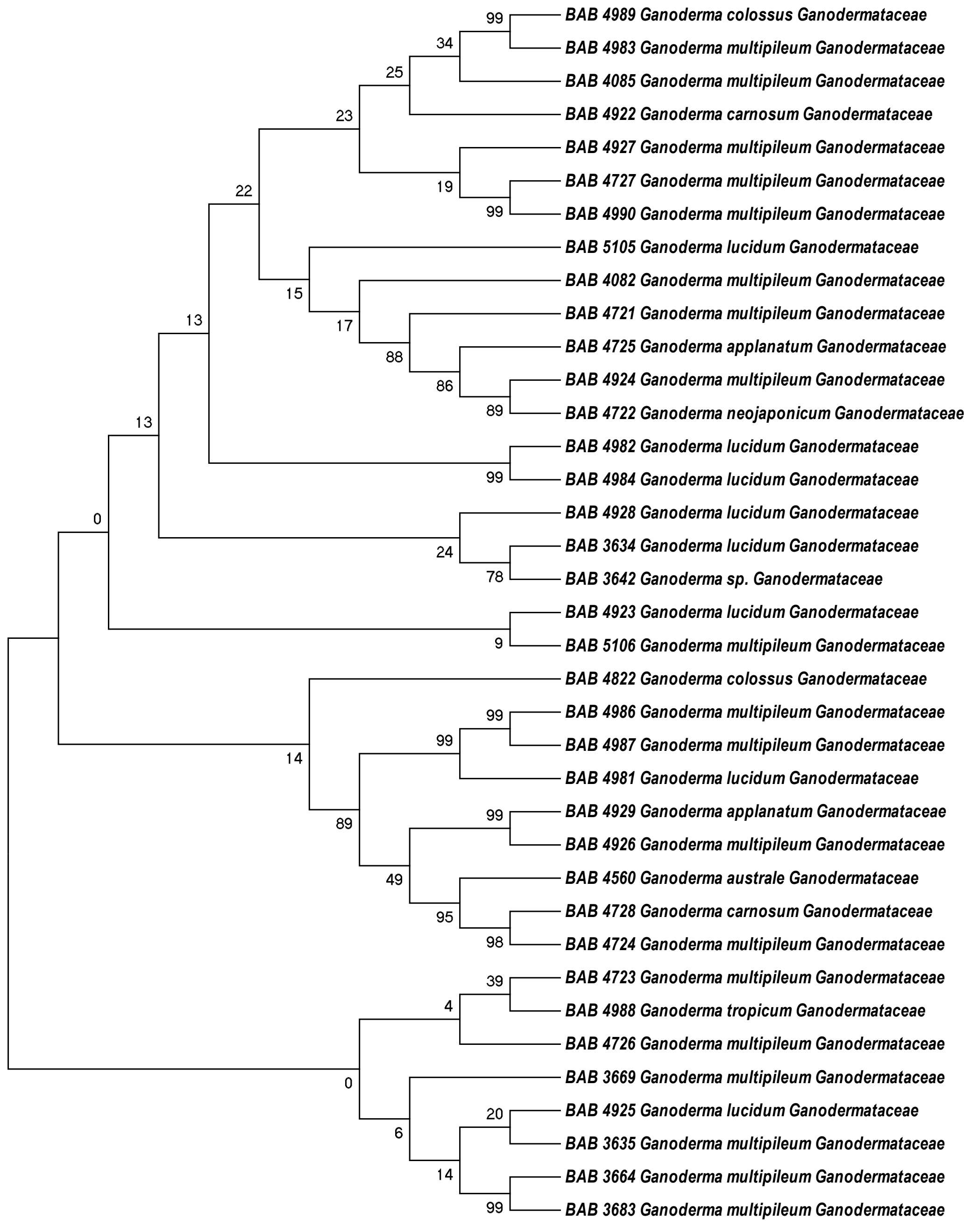

Figure S2 (I) – Phylogenetic trees generated by Maximum Likelihood method based on the Tamura-Nei model for Schizophyllaceae. The bootstrap consensus tree inferred from 500 replicates is taken to represent the evolutionary history of the taxa analyzed. Branches corresponding to partitions reproduced in less than 50% bootstrap replicates are collapsed.

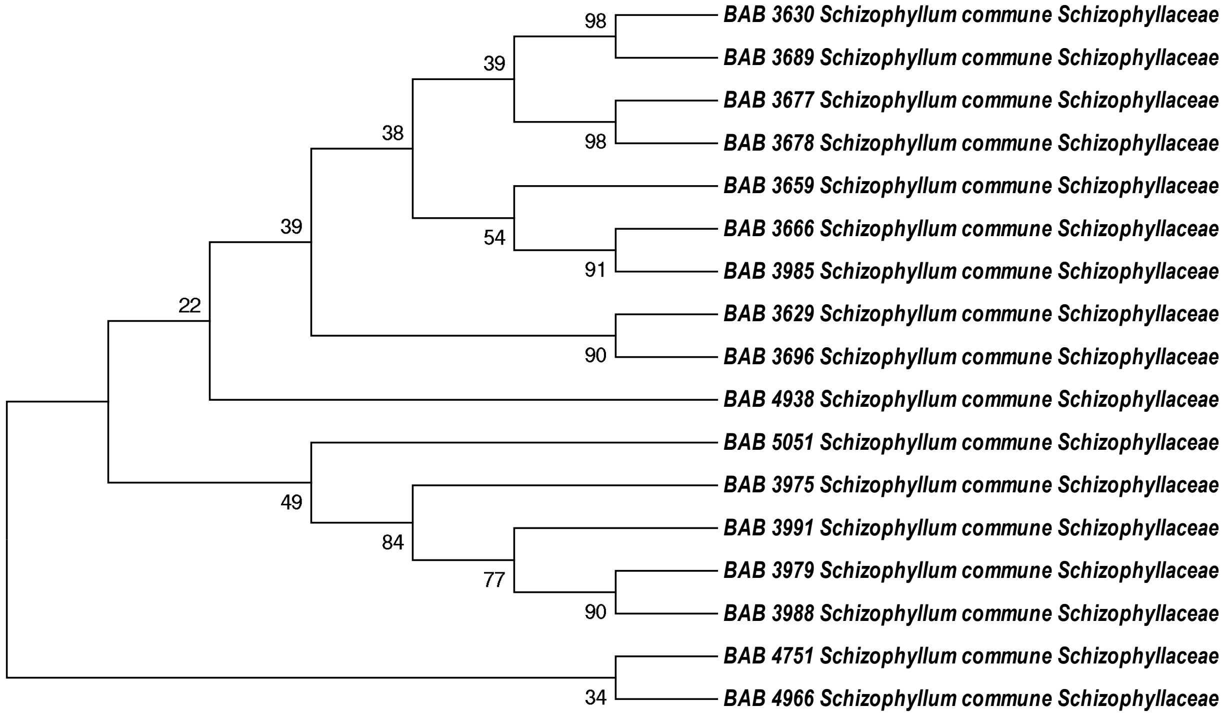

Figure S2 (J) – Phylogenetic trees generated by Maximum Parsimony method for Schizophyllaceae. The most parsimonious tree with length = 2593 is shown. The consistency index is (0.441959), the retention index is (0.471512), and the composite index is 0.208389 (0.208389) for all sites and parsimony-informative sites (in parentheses). The percentage of replicate trees in which the associated taxa clustered together in the bootstrap test (500 replicates) are shown next to the branches. The MP tree was obtained using the Subtree-Pruning-Regrafting (SPR) algorithm

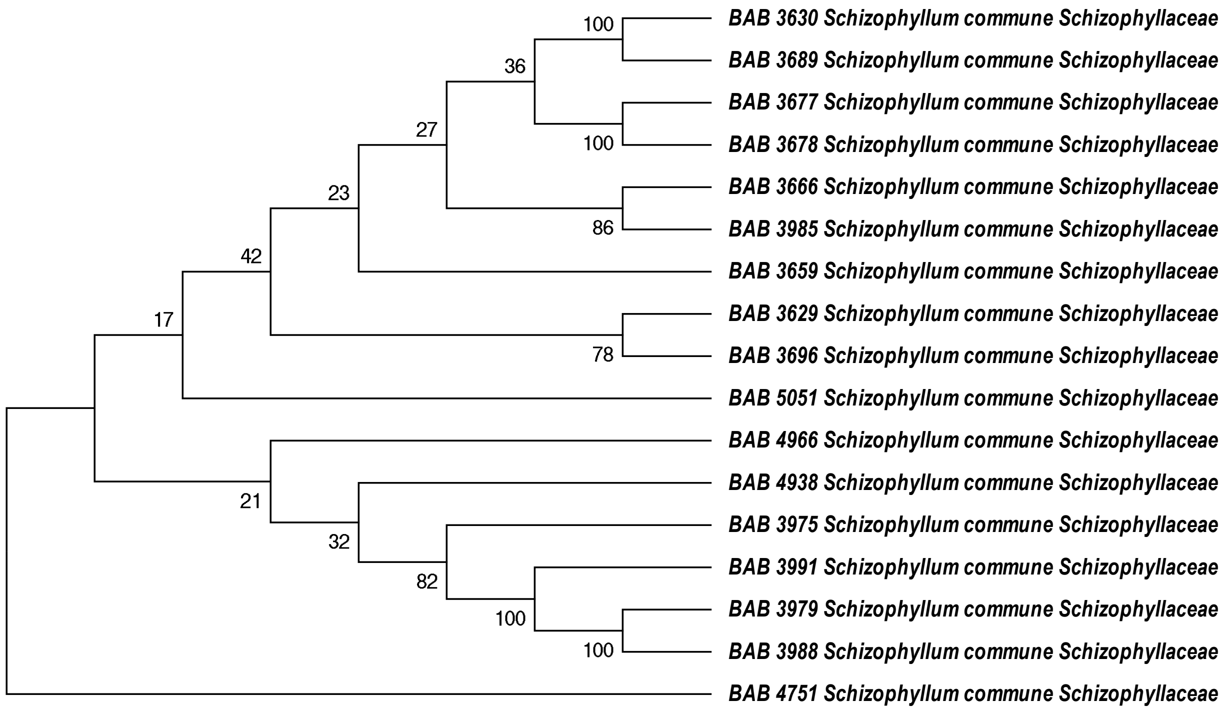

Figure S2 (K) – Phylogenetic trees generated by Maximum Likelihood method based on the Tamura-Nei model for Fomitopsidaceae. The bootstrap consensus tree inferred from 500 replicates is taken to represent the evolutionary history of the taxa analyzed. Branches corresponding to partitions reproduced in less than 50% bootstrap replicates are collapsed. The percentage of replicate trees in which the associated taxa clustered together in the bootstrap test (500 replicates) are shown next to the branches.

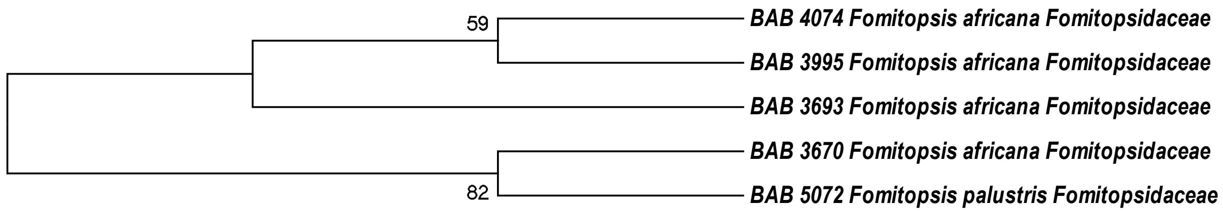

Figure S2 (L) – – Phylogenetic trees generated by Maximum Parsimony method for Fomitopsidaceae. The bootstrap consensus tree inferred from 500 replicates is taken to represent the evolutionary history of the taxa analyzed. Branches corresponding to partitions reproduced in less than 50% bootstrap replicates are collapsed. The MP tree was obtained using the Subtree-Pruning-Regrafting (SPR) algorithm

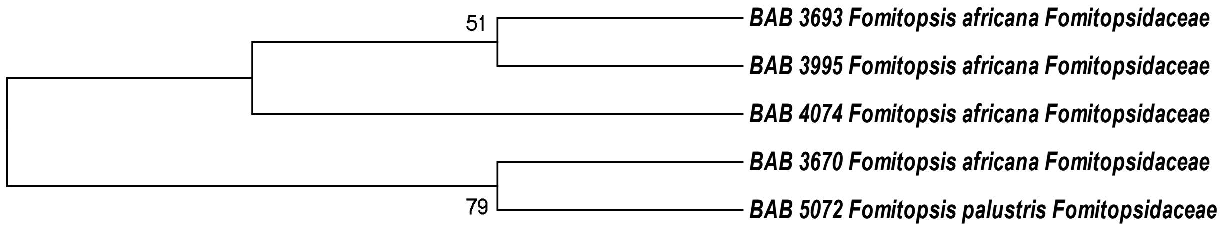

Figure S2 (M) – Phylogenetic trees generated by Maximum Likelihood method based on the Tamura-Nei model for Meruliaceae. The bootstrap consensus tree inferred from 500 replicates is taken to represent the evolutionary history of the taxa analyzed. Branches corresponding to partitions reproduced in less than 50% bootstrap replicates are collapsed. The percentage of replicate trees in which the associated taxa clustered together in the bootstrap test (500 replicates) are shown next to the branches.

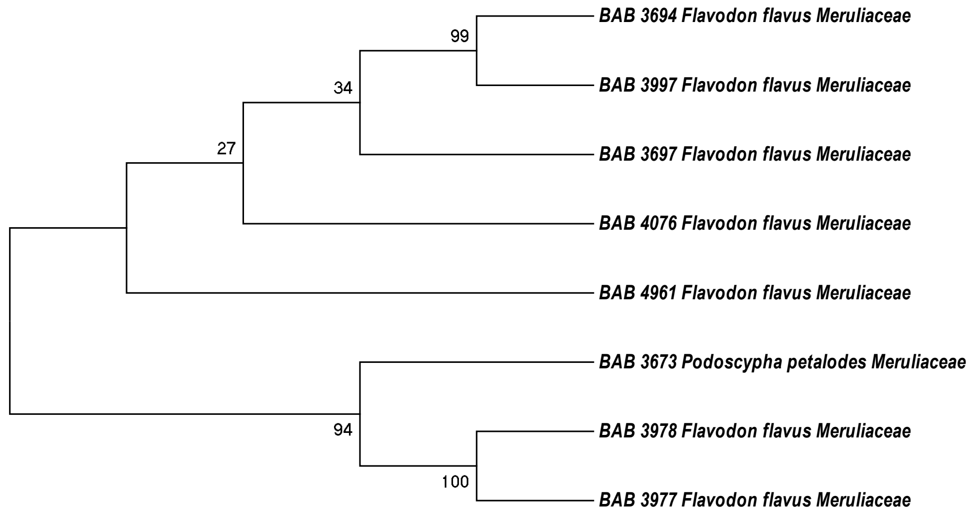

Figure S2 (N) – Phylogenetic trees generated by Maximum Likelihood method based on the Tamura-Nei model for Meruliaceae. The bootstrap consensus tree inferred from 500 replicates is taken to represent the evolutionary history of the taxa analyzed. Branches corresponding to partitions reproduced in less than 50% bootstrap replicates are collapsed. The MP tree was obtained using the Subtree-Pruning-Regrafting (SPR) algorithm

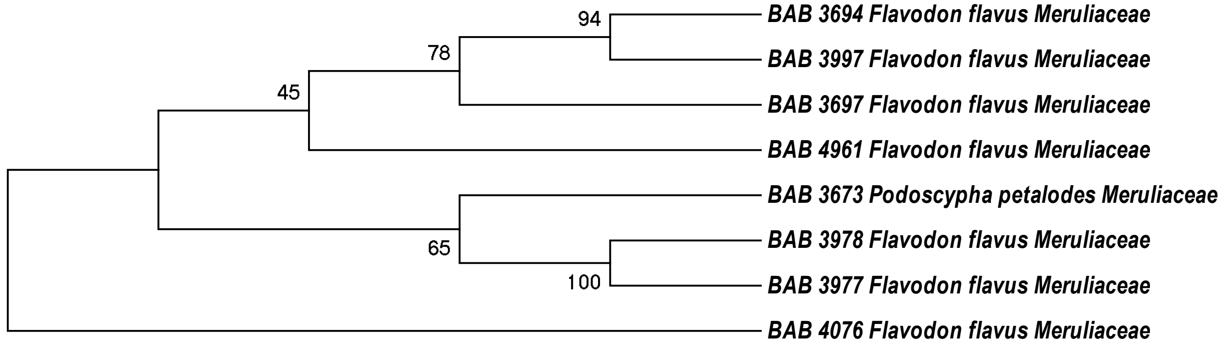

Supplement: S2 Fig — (PDF) [file pone.0197306.s002.pdf]
