# Supplementary material for: Molecular survey of basidiomycetes and divergence time estimation: An Indian perspective
Source: PLoS One. 2018 May 17;13(5):e0197306. doi: 10.1371/journal.pone.0197306 (PMC5957343; doi:10.1371/journal.pone.0197306)
Supplement: S1 Table — (DOCX) [file pone.0197306.s003.docx]

**Table S1:** Process IDs of specimen collected and accession numbers

| **Sr. No.** | **Sample ID** | **Identified mushroom** | **Process ID** | **Accession number** |
| --- | --- | --- | --- | --- |
| 1 | BAB 3629 | *Schizophyllum commune* | MGEN001-14 | KJ588789 |
| 2 | BAB 3630 | *Schizophyllum commune* | MGEN003-14 | KJ588791 |
| 3 | BAB 3631 | *Coprinellus micaceus* | MGEN004-14 | KJ588792 |
| 4 | BAB 3632 | *Calocybe indica* | MGEN005-14 | KJ588793 |
| 5 | BAB 3634 | *Ganoderma lucidum* | MGEN009-14 | KJ588794 |
| 6 | BAB 3635 | *Ganoderma multipileum* | MGEN010-14 | KJ588797 |
| 7 | BAB 3637 | *Phellinus senex* | MGEN013-14 | KJ588799 |
| 8 | BAB 3638 | *Tricholosporum porphyrophyllum* | MGEN014-14 | KJ588802 |
| 9 | BAB 3642 | *Ganoderma sp.* | MGEN020-14 | KJ588843 |
| 10 | BAB 3644 | *Lenzites sp.* | MGEN022-14 | KJ588845 |
| 11 | BAB 3645 | *Marasmius albimyceliosus* | MGEN023-14 | KJ588846 |
| 12 | BAB 3649 | *Microporus ochrotinctus* | MGEN027-14 | KJ588850 |
| 13 | BAB 3652 | *Inonotus porrectus* | MGEN031-14 | KJ588854 |
| 14 | BAB 3657 | *Leucoagaricus vassiljevae* | MGEN038-14 | KJ588822 |
| 15 | BAB 3658 | *Agaricus goossensiae* | MGEN039-14 | KJ588823 |
| 16 | BAB 3659 | *Schizophyllum commune* | MGEN040-14 | KJ588824 |
| 17 | BAB 3660 | *Colletotrichum gloeosporioides* | MGEN041-14 | KJ588825 |
| 18 | BAB 3661 | *Calocybe indica* | MGEN042-14 | KJ588826 |
| 19 | BAB 3662 | *Calocybe indica* | MGEN043-14 | KJ588827 |
| 20 | BAB 3663 | *Lenzites sp.* | MGEN044-14 | KJ588828 |
| 21 | BAB 3664 | *Ganoderma multipileum* | MGEN045-14 | KJ588829 |
| 22 | BAB 3665 | *Daldinia eschscholzii* | MGEN046-14 | KJ588830 |
| 23 | BAB 3666 | *Schizophyllum commune* | MGEN047-14 | KJ588831 |
| 24 | BAB 3667 | *Neolentinus kauffmanii* | MGEN048-14 | KJ588832 |
| 25 | BAB 3669 | *Ganoderma multipileum* | MGEN049-14 | KJ588833 |
| 26 | BAB 3670 | *Fomitopsis africana* | MGEN050-14 | KJ588834 |
| 27 | BAB 3673 | *Podoscypha petalodes* | MGEN052-14 | KJ588836 |
| 28 | BAB 3675 | *Microporus ochrotinctus* | MGEN054-14 | KJ588838 |
| 29 | BAB 3677 | *Schizophyllum commune* | MGEN056-14 | KJ588840 |
| 30 | BAB 3678 | *Schizophyllum commune* | MGEN057-14 | KJ588861 |
| 31 | BAB 3681 | *Lenzites sp.* | MGEN060-14 | KJ588864 |
| 32 | BAB 3682 | *Lenzites sp.* | MGEN061-14 | KJ588865 |
| 33 | BAB 3683 | *Ganoderma multipileum* | MGEN062-14 | KJ588866 |
| 34 | BAB 3686 | *Microporus vernicipes* | MGEN065-14 | KJ588807 |
| 35 | BAB 3689 | *Schizophyllum commune* | MGEN069-14 | KJ588821 |
| 36 | BAB 3691 | *Polyporus tricholoma* | MGEN071-14 | KJ588809 |
| 37 | BAB 3692 | *Trametes ljubarskyi* | MGEN072-14 | KJ588810 |
| 38 | BAB 3693 | *Fomitopsis africana* | MGEN073-14 | KJ588811 |
| 39 | BAB 3694 | *Flavodon flavus* | MGEN074-14 | KJ588812 |
| 40 | BAB 3696 | *Schizophyllum commune* | MGEN076-14 | KJ588814 |
| 41 | BAB 3697 | *Flavodon flavus* | MGEN077-14 | KJ588815 |
| 42 | BAB 3698 | *Trametes ljubarskyi* | MGEN078-14 | KJ588816 |
| 43 | BAB 3699 | *Polyporus tricholoma* | MGEN079-14 | KJ588817 |
| 44 | BAB 3975 | *Schizophyllum commune* | MGEN081-14 | KJ612014 |
| 45 | BAB 3976 | *Lenzites sp.* | MGEN082-14 | KJ612015 |
| 46 | BAB 3977 | *Flavodon flavus* | MGEN083-14 | KJ612016 |
| 47 | BAB 3978 | *Flavodon flavus* | MGEN084-14 | KJ612017 |
| 48 | BAB 3979 | *Schizophyllum commune* | MGEN085-14 | KJ612018 |
| 49 | BAB 3980 | *Coriolopsis caperata* | MGEN086-14 | KJ612019 |
| 50 | BAB 3983 | *Elmerina dimidiata* | MGEN089-14 | KJ612022 |
| 51 | BAB 3974 | *Lenzites sp.* | MGEN096-14 | KJ612029 |
| 52 | BAB 3971 | *Microporus cf. vernicipes* | MGEN105-14 | KJ612012 |
| 53 | BAB 3972 | *Lenzites sp.* | MGEN106-14 | KJ612013 |
| 54 | BAB 3984 | *Earliella scabrosa* | MGEN107-14 | KJ612035 |
| 55 | BAB 3985 | *Schizophyllum commune* | MGEN108-14 | KJ612036 |
| 56 | BAB 3986 | *Tricholoma giganteum* | MGEN109-14 | KJ612037 |
| 57 | BAB 3987 | *Microporus ochrotinctus* | MGEN110-14 | KJ612038 |
| 58 | BAB 3988 | *Schizophyllum commune* | MGEN111-14 | KJ612039 |
| 59 | BAB 3990 | *Coriolopsis caperata* | MGEN113-14 | KJ612041 |
| 60 | BAB 3991 | *Schizophyllum commune* | MGEN114-14 | KJ612042 |
| 61 | BAB 3992 | *Microporus ochrotinctus* | MGEN115-14 | KJ612043 |
| 62 | BAB 3993 | *Microporus ochrotinctus* | MGEN116-14 | KJ612044 |
| 63 | BAB 3995 | *Fomitopsis africana* | MGEN118-14 | KJ612046 |
| 64 | BAB 3996 | *Xylaria regalis* | MGEN119-14 | KJ612047 |
| 65 | BAB 3997 | *Flavodon flavus* | MGEN120-14 | KJ612048 |
| 66 | BAB 3998 | *Microporus ochrotinctus* | MGEN121-14 | KJ612049 |
| 67 | BAB 4073 | *Pleurotus ostreatus* | MGEN157-14 | KJ670293 |
| 68 | BAB 4074 | *Fomitopsis africana* | MGEN158-14 | KJ670294 |
| 69 | BAB 4076 | *Flavodon flavus* | MGEN159-14 | KJ670295 |
| 70 | BAB 4077 | *Amylosporus campbellii* | MGEN160-14 | KJ670296 |
| 71 | BAB 4079 | *Fulvifomes fastuosus* | MGEN162-14 | KJ670298 |
| 72 | BAB 4080 | *Podaxis pistillaris* | MGEN163-14 | KJ670299 |
| 73 | BAB 4081 | *Phellorinia herculeana* | MGEN164-14 | KJ670300 |
| 74 | BAB 4082 | *Ganoderma multipileum* | MGEN165-14 | KJ670301 |
| 75 | BAB 4083 | *Microporus vernicipes* | MGEN166-14 | KJ670302 |
| 76 | BAB 4084 | *Microporus vernicipes* | MGEN167-14 | KJ670303 |
| 77 | BAB 4085 | *Ganoderma multipileum* | MGEN168-14 | KJ670304 |
| 78 | BAB 3279 | *Daldinia eschscholzii* | MGEN176-14 | KJ612057 |
| 79 | BAB 3296 | *Daldinia eschscholzii* | MGEN181-14 | KJ612063 |
| 80 | BAB 3298 | *Daldinia eschscholzii* | MGEN182-14 | KJ612064 |
| 81 | BAB 4366 | *Dichomitus squalens* | MGEN366-14 | KM084859 |
| 82 | BAB 4560 | *Ganoderma australe* | MGEN381-14 | KR154930 |
| 83 | BAB 4561 | *Oxyporus corticola* | MGEN382-14 | KR154931 |
| 84 | BAB 4716 | *Polyporus leprieurii* | MGEN383-14 | KR154945 |
| 85 | BAB 4718 | *Lenzites betulinus* | MGEN385-14 | KR154947 |
| 86 | BAB 4719 | *Polyporus tenuiculus* | MGEN386-14 | KR154948 |
| 87 | BAB 4720 | *Auricularia polytricha* | MGEN387-14 | KR154949 |
| 88 | BAB 4721 | *Ganoderma multipileum* | MGEN388-14 | KR154950 |
| 89 | BAB 4722 | *Ganoderma neojaponicum* | MGEN389-14 | KR154951 |
| 90 | BAB 4723 | *Ganoderma multipileum* | MGEN390-14 | KR154952 |
| 91 | BAB 4724 | *Ganoderma multipileum* | MGEN391-14 | KR154953 |
| 92 | BAB 4725 | *Ganoderma applanatum* | MGEN392-14 | KR154954 |
| 93 | BAB 4726 | *Ganoderma multipileum* | MGEN393-14 | KR154955 |
| 94 | BAB 4727 | *Ganoderma multipileum* | MGEN394-14 | KR154956 |
| 95 | BAB 4728 | *Ganoderma carnosum* | MGEN395-14 | KR154957 |
| 96 | BAB 4729 | *Agaricus augustus* | MGEN396-14 | KR154958 |
| 97 | BAB 4730 | *Agaricus xanthodermus* | MGEN397-14 | KR154959 |
| 98 | BAB 4731 | *Leucocoprinus brebissonii* | MGEN398-14 | KR154960 |
| 99 | BAB 4732 | *Agaricus purpurellus* | MGEN399-14 | KR154961 |
| 100 | BAB 4733 | *Leucoagaricus littoralis* | MGEN400-14 | KR154962 |
| 101 | BAB 4734 | *Chlorophyllum hortense* | MGEN401-14 | KR154963 |
| 102 | BAB 4735 | *Leucocoprinus cretaceus* | MGEN402-14 | KR154964 |
| 103 | BAB 4736 | *Agaricus augustus* | MGEN403-14 | KR154965 |
| 104 | BAB 4737 | *Leucoagaricus rubrotinctus* | MGEN404-14 | KR154966 |
| 105 | BAB 4738 | *Lepiota flammeotincta* | MGEN405-14 | KR154967 |
| 106 | BAB 4739 | *Agaricus moelleri* | MGEN406-14 | KR154968 |
| 107 | BAB 4740 | *Agaricus placomyces* | MGEN407-14 | KR154969 |
| 108 | BAB 4741 | *Agaricus moelleri* | MGEN408-14 | KR154970 |
| 109 | BAB 4742 | *Termitomyces heimii* | MGEN409-14 | KR154971 |
| 110 | BAB 4743 | *Dichomitus squalens* | MGEN410-14 | KR154972 |
| 111 | BAB 4744 | *Coprinellus radians* | MGEN411-14 | KR154973 |
| 112 | BAB 4745 | *Coprinellus micaceus* | MGEN412-14 | KR154974 |
| 113 | BAB 4746 | *Lenzites elegans* | MGEN413-14 | KR154975 |
| 114 | BAB 4747 | *Psathyrella candolleana* | MGEN414-14 | KR154976 |
| 115 | BAB 4748 | *Psathyrella candolleana* | MGEN415-14 | KR154977 |
| 116 | BAB 4749 | *Inonotus porrectus* | MGEN416-14 | KR154978 |
| 117 | BAB 4750 | *Termitomyces microcarpus* | MGEN417-14 | KR154979 |
| 118 | BAB 4751 | *Schizophyllum commune* | MGEN418-14 | KR154980 |
| 119 | BAB 4752 | *Conocybe papillata* | MGEN419-14 | KR154981 |
| 120 | BAB 4753 | *Conocybe lactea* | MGEN420-14 | KR154982 |
| 121 | BAB 4754 | *Daedaleopsis confragosa* | MGEN421-14 | KR154983 |
| 122 | BAB 4755 | *Lyophyllum fumosum* | MGEN422-14 | KR154984 |
| 123 | BAB 4756 | *Pleurotus nebrodensis* | MGEN423-14 | KR154985 |
| 124 | BAB 4757 | *Inonotus porrectus* | MGEN424-14 | KR154986 |
| 125 | BAB 4758 | *Inonotus porrectus* | MGEN425-14 | KR154987 |
| 126 | BAB 4759 | *Xylaria regalis* | MGEN426-14 | KR154988 |
| 127 | BAB 4760 | *Psathyrella candolleana* | MGEN427-14 | KR154989 |
| 128 | BAB 4761 | *Clitopilus prunulus* | MGEN428-14 | KR154990 |
| 129 | BAB 4762 | *Termitomyces eurrhizus* | MGEN429-14 | KR154991 |
| 130 | BAB 4763 | *Mycena corynephora* | MGEN430-14 | KR154992 |
| 131 | BAB 4764 | *Lepista sordida* | MGEN431-14 | KR154993 |
| 132 | BAB 4765 | *Lenzites elegans* | MGEN432-14 | KR154994 |
| 133 | BAB 4818 | *Fulvifomes fastuosus* | MGEN485-15 | KR154999 |
| 134 | BAB 4821 | *Itajahya rosea* | MGEN488-15 | KR155002 |
| 135 | BAB 4822 | *Ganoderma colossus* | MGEN489-15 | KR155003 |
| 136 | BAB 4981 | *Ganoderma lucidum* | MGEN492-15 | KR155071 |
| 137 | BAB 4922 | *Ganoderma carnosum* | MGEN493-15 | KR155019 |
| 138 | BAB 4929 | *Ganoderma applanatum* | MGEN494-15 | KR155025 |
| 139 | BAB 4923 | *Ganoderma lucidum* | MGEN495-15 | KR155020 |
| 140 | BAB 4986 | *Ganoderma multipileum* | MGEN497-15 | KR155074 |
| 141 | BAB 4987 | *Ganoderma multipileum* | MGEN498-15 | KR155075 |
| 142 | BAB 4988 | *Ganoderma tropicum* | MGEN499-15 | KR155076 |
| 143 | BAB 4989 | *Ganoderma colossus* | MGEN500-15 | KR155077 |
| 144 | BAB 4926 | *Ganoderma multipileum* | MGEN501-15 | KR155023 |
| 145 | BAB 4924 | *Ganoderma multipileum* | MGEN502-15 | KR155021 |
| 146 | BAB 4925 | *Ganoderma lucidum* | MGEN503-15 | KR155022 |
| 147 | BAB 4927 | *Ganoderma multipileum* | MGEN504-15 | KR155024 |
| 148 | BAB 4984 | *Ganoderma lucidum* | MGEN505-15 | KR349639 |
| 149 | BAB 4928 | *Ganoderma lucidum* | MGEN506-15 | KR349640 |
| 150 | BAB 4990 | *Ganoderma multipileum* | MGEN507-15 | KR155078 |
| 151 | BAB 4982 | *Ganoderma lucidum* | MGEN508-15 | KR349638 |
| 152 | BAB 4983 | *Ganoderma multipileum* | MGEN509-15 | KR155072 |
| 153 | BAB 4913 | *Psathyrella candolleana* | MGEN511-15 | KR155010 |
| 154 | BAB 4914 | *Clitopilus prunulus* | MGEN512-15 | KR155011 |
| 155 | BAB 4917 | *Itajahya rosea* | MGEN515-15 | KR155014 |
| 156 | BAB 4970 | *Agaricus blazei* | MGEN590-15 | KR155060 |
| 157 | BAB 4971 | *Agaricus hondensis* | MGEN591-15 | KR155061 |
| 158 | BAB 4972 | *Lepiota subclypeolaria* | MGEN592-15 | KR155062 |
| 159 | BAB 4973 | *Leucoagaricus hortensis* | MGEN593-15 | KR155063 |
| 160 | BAB 4974 | *Agaricus langei* | MGEN594-15 | KR155064 |
| 161 | BAB 4975 | *Agaricus augustus* | MGEN595-15 | KR155065 |
| 162 | BAB 4976 | *Agaricus pocillator* | MGEN596-15 | KR155066 |
| 163 | BAB 4978 | *Agaricus rotalis* | MGEN598-15 | KR155068 |
| 164 | BAB 4979 | *Lepiota subclypeolaria* | MGEN599-15 | KR155069 |
| 165 | BAB 4980 | *Lepiota subclypeolaria* | MGEN600-15 | KR155070 |
| 166 | BAB 4937 | *Entoloma flocculosum* | MGEN601-15 | KR155027 |
| 167 | BAB 4938 | *Schizophyllum commune* | MGEN602-15 | KR155028 |
| 168 | BAB 4939 | *Psathyrella candolleana* | MGEN603-15 | KR155029 |
| 169 | BAB 4940 | *Tricholoma giganteum* | MGEN604-15 | KR155030 |
| 170 | BAB 4941 | *Clitocybe metachroa* | MGEN605-15 | KR155031 |
| 171 | BAB 4942 | *Inonotus porrectus* | MGEN606-15 | KR155032 |
| 172 | BAB 4943 | *Polyporus grammocephalus* | MGEN607-15 | KR155033 |
| 173 | BAB 4944 | *Collybia hariolorum* | MGEN608-15 | KR155034 |
| 174 | BAB 4945 | *Clitopilus prunulus* | MGEN609-15 | KR155035 |
| 175 | BAB 4946 | *Psathyrella candolleana* | MGEN610-15 | KR155036 |
| 176 | BAB 4947 | *Tricholoma mongolicum* | MGEN611-15 | KR155037 |
| 177 | BAB 4948 | *Omphalina rivulicola* | MGEN612-15 | KR155038 |
| 178 | BAB 4949 | *Psathyrella candolleana* | MGEN613-15 | KR155039 |
| 179 | BAB 4951 | *Geastrum striatum* | MGEN615-15 | KR155041 |
| 180 | BAB 4952 | *Xylaria psidii* | MGEN616-15 | KR155042 |
| 181 | BAB 4953 | *Termitomyces eurrhizus* | MGEN617-15 | KR155043 |
| 182 | BAB 4954 | *Phellinus merrillii* | MGEN618-15 | KR155044 |
| 183 | BAB 4955 | *Peziza arvernensis* | MGEN619-15 | KR155045 |
| 184 | BAB 4956 | *Perenniporia tephropora* | MGEN620-15 | KR155046 |
| 185 | BAB 4957 | *Hypoxylon rickii* | MGEN621-15 | KR155047 |
| 186 | BAB 4958 | *Perenniporia tephropora* | MGEN622-15 | KR155048 |
| 187 | BAB 4959 | *Clitopilus prunulus* | MGEN623-15 | KR155049 |
| 188 | BAB 4960 | *Phellinus bicuspidatus* | MGEN624-15 | KR155050 |
| 189 | BAB 4961 | *Flavodon flavus* | MGEN625-15 | KR155051 |
| 190 | BAB 4963 | *Coprinus silvaticus* | MGEN626-15 | KR155053 |
| 191 | BAB 4964 | *Psathyrella candolleana* | MGEN627-15 | KR155054 |
| 192 | BAB 4965 | *Phellinus robiniae* | MGEN628-15 | KR155055 |
| 193 | BAB 4966 | *Schizophyllum commune* | MGEN629-15 | KR155056 |
| 194 | BAB 4967 | *Clitopilus prunulus* | MGEN630-15 | KR155057 |
| 195 | BAB 4968 | *Phellinus merrillii* | MGEN631-15 | KR155058 |
| 196 | BAB 4969 | *Hypoxylon rickii* | MGEN632-15 | KR155059 |
| 197 | BAB 5051 | *Schizophyllum commune* | MGEN665-15 | KR155096 |
| 198 | BAB 5052 | *Ceriporia lacerata* | MGEN666-15 | KR155097 |
| 199 | BAB 5055 | *Amylosporus campbellii* | MGEN667-15 | KR155100 |
| 200 | BAB 5056 | *Clitopilus scyphoides* | MGEN668-15 | KR155101 |
| 201 | BAB 5063 | *Termitomyces eurrhizus* | MGEN670-15 | KR155108 |
| 202 | BAB 5064 | *Dacryopinax spathularia* | MGEN671-15 | KR155109 |
| 203 | BAB 5065 | *Fulvifomes fastuosus* | MGEN672-15 | KR155110 |
| 204 | BAB 5067 | *Psathyrella candolleana* | MGEN673-15 | KR155112 |
| 205 | BAB 5069 | *Itajahya rosea* | MGEN675-15 | KR155114 |
| 206 | BAB 5070 | *Coprinopsis cinerea* | MGEN676-15 | KR155115 |
| 207 | BAB 4962 | *Clitopilus prunulus* | MGEN678-15 | KR155052 |
| 208 | BAB 5048 | *Lycoperdon pusillum* | MGEN679-15 | KR155093 |
| 209 | BAB 5050 | *Leucoagaricus leucothites* | MGEN680-15 | KR155095 |
| 210 | BAB 5053 | *Lepiota flammeotincta* | MGEN681-15 | KR155098 |
| 211 | BAB 5057 | *Agaricus diminutivus* | MGEN682-15 | KR155102 |
| 212 | BAB 5058 | *Podaxis pistillaris* | MGEN683-15 | KR155103 |
| 213 | BAB 5059 | *Agaricus romagnesii* | MGEN684-15 | KR155104 |
| 214 | BAB 5071 | *Podaxis pistillaris* | MGEN685-15 | KR155116 |
| 215 | BAB 5104 | *Leucocoprinus fragilissimus* | MGEN686-15 | KR155122 |
| 216 | BAB 5054 | *Agaricus trisulphuratus* | MGEN687-15 | KR155099 |
| 217 | BAB 5072 | *Fomitopsis palustris* | MGEN690-15 | KR155117 |
| 218 | BAB 5105 | *Ganoderma lucidum* | MGEN694-15 | KR155123 |
| 219 | BAB 5106 | *Ganoderma multipileum* | MGEN695-15 | KR155124 |
| 220 | BAB 5118 | *Phellinus robiniae* | MGEN700-15 | KR349652 |
| 221 | BAB 5119 | *Agaricus californicus* | MGEN701-15 | KR349647 |
| 222 | BAB 5120 | *Scytinopogon sp.* | MGEN702-15 | KT804576 |
| 223 | BAB 5121 | *Trametes trogii* | MGEN703-15 | KT804577 |
